# A peroxisomal β-oxidative pathway contributes to the formation of C_6_–C_1_ aromatic volatiles in poplar

**DOI:** 10.1093/plphys/kiab111

**Published:** 2021-03-16

**Authors:** Nathalie D Lackus, Axel Schmidt, Jonathan Gershenzon, Tobias G Köllner

**Affiliations:** Department of Biochemistry, Max Planck Institute for Chemical Ecology, Hans-Knöll-Straße 8, D-07745 Jena, Germany

## Abstract

Benzenoids (C_6_–C_1_ aromatic compounds) play important roles in plant defense and are often produced upon herbivory. Black cottonwood (*Populus trichocarpa*) produces a variety of volatile and nonvolatile benzenoids involved in various defense responses. However, their biosynthesis in poplar is mainly unresolved. We showed feeding of the poplar leaf beetle (*Chrysomela populi*) on *P. trichocarpa* leaves led to increased emission of the benzenoid volatiles benzaldehyde, benzylalcohol, and benzyl benzoate. The accumulation of salicinoids, a group of nonvolatile phenolic defense glycosides composed in part of benzenoid units, was hardly affected by beetle herbivory. In planta labeling experiments revealed that volatile and nonvolatile poplar benzenoids are produced from cinnamic acid (C_6_–C_3_). The biosynthesis of C_6_–C_1_ aromatic compounds from cinnamic acid has been described in petunia (*Petunia hybrida*) flowers where the pathway includes a peroxisomal-localized chain shortening sequence, involving cinnamate-CoA ligase (CNL), cinnamoyl-CoA hydratase/dehydrogenase (CHD), and 3-ketoacyl-CoA thiolase (KAT). Sequence and phylogenetic analysis enabled the identification of small *CNL*, *CHD*, and *KAT* gene families in *P. trichocarpa*. Heterologous expression of the candidate genes in *Escherichia coli* and characterization of purified proteins in vitro revealed enzymatic activities similar to those described in petunia flowers. RNA interference-mediated knockdown of the *CNL* subfamily in gray poplar (*Populus* x *canescens*) resulted in decreased emission of C_6_–C_1_ aromatic volatiles upon herbivory, while constitutively accumulating salicinoids were not affected. This indicates the peroxisomal β-oxidative pathway participates in the formation of volatile benzenoids. The chain shortening steps for salicinoids, however, likely employ an alternative pathway.

## Teaser

A three-step peroxisomal β-oxidative pathway mediates the shortening of the propyl side chain of cinnamic acid and contributes to herbivore-induced aromatic volatile formation in poplar.

## Introduction

As sessile organisms, plants have to cope with a variety of biotic stressors, such as herbivores and pathogens. To defend themselves, plants produce a huge variety of specialized metabolites, which can either act as direct defense compounds against the herbivores or pathogens, as indirect defense, for instance through the attraction of parasitoids or predators, or as defense signals (Unsicker et al., 2009; Mithöfer and Boland, 2012; Fürstenberg-Hägg et al., 2013; Maag et al., 2015). Estimates assume that around 200,000 to 1 million structurally different compounds are produced as specialized metabolites by plants (Pichersky and Lewinsohn, 2011). These metabolites can be classified based on their biosynthetic origin or common chemical core structures, and the three major compound classes are terpenes, nitrogen-containing compounds, and aromatic compounds (Dudareva et al., 2013; Muhlemann et al., 2014; Widhalm and Dudareva, 2015).

Aromatic compounds form a highly diverse group of specialized metabolites that widely occur within the plant kingdom. Chemically, aromatic compounds are defined by their mostly planar and cyclic ring structures with conjugated double bonds (Vogt, 2010; Muhlemann et al., 2014; Widhalm and Dudareva, 2015). The further classification of aromatic compounds is based on the length of the side chain with the categories: phenylpropanoids (C_6_–C_3_), phenylpropanoid-related (C_6_–C_2_), and benzenoid (C_6_–C_1_) compounds (Vogt, 2010; Muhlemann et al., 2014; Widhalm and Dudareva, 2015). The biosynthesis of most aromatic compounds starts from the aromatic amino acid l-phenylalanine and its deamination to cinnamic acid (C_6_–C_3_), catalyzed by phenylalanine ammonia lyase (MacDonald and D'Cunha, 2007). Essential for the further formation of benzenoids from cinnamic acid is the shortening of the propyl side chain by two carbons (Vogt, 2010; Widhalm and Dudareva, 2015). Multiple chain shortening pathways have been postulated in the literature: two cytosolic nonoxidative pathways, either CoA-dependent or CoA-independent, and a β-oxidative pathway in the peroxisomes (Boatright et al., 2004; Widhalm and Dudareva, 2015). Whereas both cytosolic pathways are poorly understood, the β-oxidative pathway has been elucidated in petunia (*Petunia hybrida*) flowers and partially in Arabidopsis (*Arabidopsis thaliana*; Van Moerkercke et al., 2009; Colquhoun et al., 2012; Klempien et al., 2012; Lee et al., 2012; Qualley et al., 2012; Bussell et al., 2014). The β-oxidative pathway in the peroxisomes is analogous to fatty acid degradation and mediates the C_2_ shortening of the propyl side chain over three enzymatic steps (Widhalm and Dudareva, 2015). The first step in this pathway, the activation of cinnamic acid to cinnamoyl-CoA, is catalyzed by cinnamate-CoA ligase (CNL; Colquhoun et al., 2012; Gaid et al., 2012; Klempien et al., 2012). The cinnamoyl-CoA formed serves as substrate for the bifunctional CHD, which first mediates the hydration of the propyl side chain and then its further oxidation to 3-oxo-3-phenylpropanoyl-CoA (Qualley et al., 2012). The actual shortening of the propyl side chain is catalyzed by 3-ketoacyl-CoA thiolase (KAT), leading to the formation of the first C_6_–C_1_ intermediate, benzoyl-CoA (Van Moerkercke et al., 2009). The benzoyl-CoA produced acts as precursor for the formation of volatile and nonvolatile benzenoids, as benzyl benzoate or benzoic acid (-derivatives) (e.g. Boatright et al., 2004; Chedgy et al., 2015; Adebesin et al., 2018; Singh et al., 2020). So far, the peroxisomal β-oxidative pathway has been only investigated in two plant species. Thus, how widespread this pathway occurs within the plant kingdom and whether this pathway contributes to the formation of volatile and nonvolatile benzenoids, particularly in vegetative tissues, is still unresolved.

Aromatic compounds, especially benzenoids, are characteristic components of plant volatile bouquets and can be emitted from both floral and vegetative tissues (Knudsen et al., 2006; Schiestl, 2010; Dudareva et al., 2013; Muhlemann et al., 2014; Amrad et al., 2016; Sas et al., 2016). Floral volatiles are often involved in the attraction of pollinators, whereas the volatile emission by vegetative tissues is mainly induced upon herbivory and the released compounds contribute to direct and indirect plant defense responses (Knudsen et al., 2006; Unsicker et al., 2009; Amrad et al., 2016; Sas et al., 2016). Apart from their emission as volatiles, benzenoid specialized metabolites can also accumulate as nonvolatiles in the plant tissue, as benzoic acid or its derivatives (Widhalm and Dudareva, 2015). Moreover, nonvolatile benzenoids are often glycosylated, and thus can be stored as defense compounds in the vacuole (e.g. Jones and Vogt, 2001; Böckler et al., 2011; Gleadow and Møller, 2014).

The Salicaceae, comprising poplar, willow, and aspen trees, is a plant family known to produce a wide array of volatile and nonvolatile aromatic compounds. Poplars, for example, have been reported to emit vegetative volatile blends rich in benzenoids. Herbivory of gypsy moth (*Lymantria dispar*) caterpillars on poplar leaves led, among other compounds, to the induced emission of benzaldehyde, benzylalcohol, benzyl benzoate, and methyl salicylate (Danner et al., 2011; McCormick et al., 2014; Fabisch et al., 2019). Furthermore, behavioral assays revealed that benzaldehyde emitted from poplar deters the *L. dispar* caterpillars, indicating a biological function of the benzenoid volatiles in plant defense (Eberl et al., 2018). Although poplar benzenoids are characteristic components of the herbivore-induced volatile bouquet in these species and contribute to the plant defense, their biosynthesis is largely unknown.

Beside volatile benzenoids, poplars and willows form another group of nonvolatile C_6_–C_1_ phenolic glycosides called salicinoids. Salicinoids are known as effective defense compounds against various insect and mammalian herbivores, as *L. dispar* caterpillars or field voles (*Microtus agrestis*; e.g. Lindroth et al., 1988; Lindroth and Peterson, 1988; Osier et al. 2000; Bailey et al., 2006; Heiska et al., 2007; Feistel et al., 2017). Structurally, salicinoids are defined by a 2-(hydroxymethyl)phenyl-β-d-glucopyranoside core structure, which describes the simplest salicinoid salicin (Böckler et al., 2011). Derivatives of this core structure are referred to as complex salicinoids, as salicortin or tremulacin, which possess either one or two additional benzenoid or benzenoid-related moieties, respectively (Böckler et al., 2011; Feistel et al., 2015). Although salicinoids have long been investigated, knowledge of their biosynthesis remains elusive. Inhibition of the phenylalanine ammonia lyase in bay willow (*Salix pentandra*) shoot tips revealed that l-phenylalanine and its conversion to cinnamic acid is essential for biosynthesis of salicinoids in planta (Ruuhola and Julkunen-Tiitto, 2003). Feeding experiments with isotopically labeled compounds indicated that the benzenoids benzaldehyde, benzylalcohol, and salicylaldehyde might be intermediates in the biosynthesis of salicinoids (Zenk, 1967; Babst et al., 2010). More recently, two acyltransferases have been identified in poplar, catalyzing the formation of benzyl benzoate and salicyl benzoate in vitro (Chedgy et al., 2015). Both compounds are proposed as likely intermediates in the biosynthesis of complex salicinoids. Moreover, the current finding of a uridine diphosphate (UDP)-dependent glycosyltransferase able to glycosylate salicyl benzoate, and its CRISPR/Cas9-mediated knockout, supports this biosynthetic pathway (Fellenberg et al., 2019). However, detailed knowledge about the formation of the benzenoid core structure of salicinoids is not yet available.

The aim of this study was to investigate the biosynthesis of volatile and nonvolatile benzenoids in black cottonwood (*Populus trichocarpa*, Salicaceae). Herbivory of the poplar leaf beetle *Chrysomela populi* on *P. trichocarpa* leaves led to the induced emission of a complex volatile blend including benzaldehyde, benzylalcohol, and benzyl benzoate. However, salicinoids as nonvolatile benzenoids were only partially altered through the herbivore treatment. Using phylogenetic and transcriptome analysis, we identified candidate genes of a putative peroxisomal β-oxidative pathway in *P. trichocarpa*. Heterologous expression and biochemical characterization of the respective enzymes revealed their expected enzymatic activity in vitro. To evaluate a potential contribution of the β-oxidative pathway to the formation of salicinoids and herbivore-induced volatile benzenoids in vivo, we performed a RNA-interference (RNAi) mediated knockdown of the *CNL* subfamily in gray poplar (*Populus* x *canescens*).

## Results

### Herbivory of *C. populi* beetles induces the production of benzenoid volatiles, but nonvolatile benzenoid compounds are barely affected

**Figure 1:**
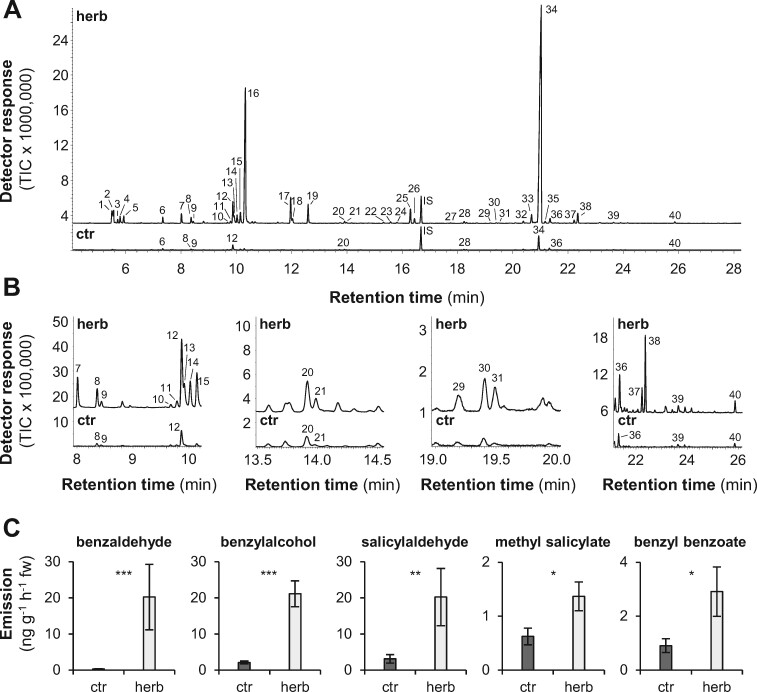
Volatiles emitted from undamaged (ctr) and *Chrysomela populi-*damaged (herb) *P. trichocarpa* leaves. Volatile profiles of control and herbivore-damaged *P. trichocarpa* leaves (A), and enlargements of the volatile profiles (B) measured and analyzed using GC-MS, and quantification of benzenoid volatiles (C). Means and se are shown (*n* = 8). 1, (*E*)-3-methylbutyraldoxime; 2, (*E*)-2-methylbutyraldoxime; 3, (*Z*)-2-methylbutyraldoxime; 4, 1-hexanol; 5, (*Z*)-3-methylbutyraldoxime; 6, α-pinene^#^; 7, benzaldehyde^#^; 8, sabinene; 9, β-pinene^#^; 10, *p*-cymene; 11, limonene^#^; 12, 1,8 cineole; 13, benzylalcohol^#^; 14, (*Z*)-β-ocimene; 15, salicylaldehyde^#^; 16, (*E*)-β-ocimene^#^; 17, 2-phenylethanol^#^; 18, (*E*)-4,8-dimethylnona-1,3,7-triene; 19, benzyl cyanide; 20, α-terpineol^#^; 21, methyl salicylate^#^; 22, (*E*)-phenylacetaldoxime; 23, salicylalcohol; 24, (*Z*)-phenylacetaldoxime; 25, indole; 26, 2-phenylnitroethane; IS, internal standard (nonyl acetate); 27, eugenol; 28, α-copaene; 29, calarene; 30, β-cubebene; 31, isoamyl benzoate; 32, α-amorphene; 33, (*Z*,*E*)-α-farnesene; 34, (*E*,*E*)-α-farnesene; 35, γ-cadinene; 36, δ-cadinene; 37, *cis*-3-hexenyl benzoate^#^; 38, (*E*,*E*)-4,8,12-trimethyltrideca-1,3,7,11-tetraene/hexenyl benzoate; 39, cadinol; 40, benzyl benzoate^#^. Compounds marked with^#^ were identified by comparison of retention time and mass spectrum to those of authentic standards. Other compounds were identified by database comparisons. fw, fresh weight; TIC, total ion chromatogram. Asterisks indicate statistical significance as assed by Student's *t* test (ST) or Mann-Whitney Rank Sum Test (MW) (*P <* *0.05; **P <* *0.01; ***P <* *0.001). benzaldehyde (*P *<* *0.001, *T* = 36.000, MW); benzylalcohol (*P *<* *0.001, *T* = 36.000, MW); salicylaldehyde (*P *=* *0.007, *T* = 43.000, MW); methyl salicylate (*P *=* *0.031, *t* = −2.398, ST); benzyl benzoate (*P *=* *0.036, *t* = −2.315, ST).

Herbivory often results in the induced formation of specialized metabolites. For example, feeding of the generalist herbivore *L. dispar* on poplar leaves has been reported to induce the formation of a wide array of defense compounds (Danner et al., 2011; Irmisch et al., 2013; McCormick et al., 2014; Fabisch et al., 2019). To test the influence of a specialist herbivore on poplar-induced defense compounds, we investigated the formation of volatile and nonvolatile specialized metabolites in leaves of *P. trichocarpa* upon herbivory of the poplar leaf beetle *C. populi*. Volatile collection and subsequent gas chromatography-mass spectrometry (GC-MS) analysis of trapped compounds revealed that herbivory of *C. populi* beetles led to the emission of a complex volatile bouquet comprising terpenes, nitrogen-containing compounds, and aromatic compounds (Figure 1; Supplemental Table S1). The most abundant herbivore-induced volatiles were identified as the monoterpene (*E*)-β-ocimene and the sesquiterpene (*E*,*E*)-α-farnesene (Figure 1; Supplemental Table S1). Moreover, the volatile bouquet contained a variety of benzenoid compounds such as benzaldehyde, benzylalcohol, salicylaldehyde, methyl salicylate, and benzyl benzoate (Figure 1; Supplemental Table S1) whose emission was significantly induced in response to the herbivore treatment (Figure 1; Supplemental Table S1). The accumulation of volatile benzenoids in the *P. trichocarpa* leaf tissue was also induced upon *C. populi* herbivory (Supplemental Table S2).

Additionally, we measured the concentration of nonvolatile specialized metabolites such as the highly abundant salicinoids and aromatic carboxylic acids in undamaged and beetle-damaged *P. trichocarpa* leaves using liquid chromatography/UV detection (HPLC/UV) and liquid chromatography/tandem mass spectrometry (LC-MS/MS), respectively. Analysis of the salicinoid content revealed that the herbivore treatment did not induce the accumulation of complex salicinoids, salirepin, and the sulfated salicinoids salicin-7-sulfate and salirepin-7-sulfate (Table 1). However, *C. populi* herbivory resulted in a significantly increased accumulation of salicin (Table 1). Further LC-MS/MS analysis revealed that the accumulation of the aromatic carboxylic acids 4-hydroxycinnamic acid, caffeic acid, ferulic acid, 4-hydroxybenzoic acid, 2,3-dihydroxybenzoic acid, and 2,5-dihydroxybenzoic acid was significantly induced in response to herbivory (Supplemental Table S3). The concentration of the most abundant aromatic carboxylic acid, cinnamic acid, however, was not influenced by the herbivore treatment. Moreover, the accumulation of salicylic acid, a phytohormone with benzenoid carboxylic acid structure, was not significantly influenced by the herbivore-treatment (Supplemental Table S4)

**Table 1:** Salicinoid content (mg/g dry weight) in undamaged (control) and *Chrysomela populi-*damaged (herbivory) *P. trichocarpa* leaves

Compound	Control	Herbivory	*P*-value	*t*-value
Salicin	0.91 ± 0.13	1.70 ± 0.12	<0.001	−4.711 (ST)
Salicin-7-sulfate	2.47 ± 0.09	2.61 ± 0.13	0.395	−0.877 (ST)
Salirepin	1.70 ± 0.11	1.78 ± 0.06	0.535	−0.637 (ST)
Salirepin-7-sulfate	1.39 ± 0.09	1.48 ± 0.07	0.440	−0.794 (ST)
Salicortin	122.81 ± 2.41	123.48 ± 1.87	0.817	−0.236 (ST)
Homaloside D	23.96 ± 0.77	23.99 ± 0.69	0.982	−0.0227 (ST)
Tremulacin	20.43 ± 0.83	19.87 ± 0.55	0.560	0.597 (ST)
6-*O′*-benzoylsalicortin	3.89 ± 0.22	3.99 ± 0.18	0.730	−0.352 (ST)

Compounds were extracted with methanol from freeze-dried plant material and analyzed using LC-MS/MS or HPLC-UV. Means and se (*n* = 8) are given. Differences between treatments were analyzed by Stdent's *t* test (ST).

### Volatile and nonvolatile benzenoids are produced from cinnamic acid

**Figure 2:**
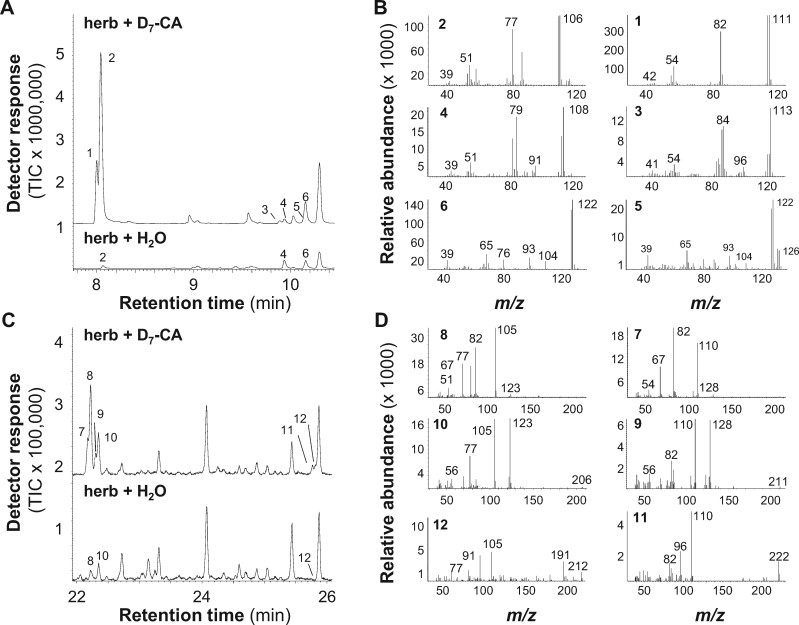
Benzenoid volatiles emitted from *Chrysomela populi-*damaged *P. trichocarpa* leaves (herb) incubated with water (H_2_O) or D_7_-cinnamic acid (D_7_-CA). Volatiles were measured and analyzed using GC-MS. Volatile profiles (A and C) and mass spectra (B and D) of unlabeled and deuterium-labeled benzenoid volatiles are displayed. 1, D_5_-benzaldehyde; 2, benzaldehyde^#^; 3, D_5_-benzylalcohol; 4, benzylalcohol^#^; 5, D_4_-salicylaldehyde; 6, salicylaldehyde^#^; 7, D_5_-*cis*-3-hexenyl benzoate; 8, *cis*-3-hexenyl benzoate^#^; 9, D_5_-hexenyl benzoate; 10, hexenyl benzoate; 11, D_10_-benzyl benzoate; 12, benzyl benzoate^#^. Compounds marked with ^#^ were identified by comparison of retention time and mass spectrum to those of authentic standards. Other compounds were identified by database comparisons. TIC, total ion chromatogram.

Cinnamic acid is a common precursor in the formation of aromatic specialized metabolites in plants (Vogt, 2010; Widhalm and Dudareva, 2015). To test whether cinnamic acid serves as a precursor in the biosynthesis of volatile and nonvolatile benzenoids in poplar, we fed deuterium-labeled D_7_-cinnamic acid to detached *P. trichocarpa* leaves that were damaged by *C. populi* beetles. Volatile collection was conducted and the collected volatiles were subsequently analyzed via GC-MS to identify mass shifts of the molecular ions and corresponding fragments. Mass shifts indicating the incorporation of the labeled precursor were observed for the molecular ions of benzaldehyde (*m/z* 106 → *m/z* 111), benzylalcohol (*m/z* 108 → *m/z* 113), salicylaldehyde (*m/z* 122 → *m/z* 126), benzyl benzoate (*m/z* 212 → *m/z* 222), hexenyl benzoate (*m/z* 206 → *m/z* 211), and the main fragment of *cis*-3-hexenyl benzoate (*m/z* 105 → *m/z* 110; Figure 2). Methyl salicylate, salicylalcohol, and isoamyl benzoate could not be detected in nonlabeled and labeled volatile bouquets, although they were found in the volatile blend released from nondetached herbivore-damaged poplar leaves in the experiment described above (Figure 1; Supplemental Table S1).

To investigate the uptake of D_7_-cinnamic acid and its incorporation into nonvolatile salicinoids and aromatic carboxylic acids, we prepared methanol extracts of the leaf material and conducted LC-MS/MS measurements in multiple reaction monitoring mode. The uptake of D_7_-cinnamic acid was proven by the appearance of D_7_-cinnamic acid in the leaf tissue itself, and we further showed its metabolism into labeled forms of different aromatic carboxylic acids, such as 4-hydroxycinnamic acid or caffeic acid (Supplemental Table S5). Moreover, feeding of D_7_-cinnamic acid resulted in label incorporation into the 2-(hydroxymethyl)phenol moiety of all analyzed salicinoids (Table 2). Further analysis of the mass spectra of the complex salicinoids revealed label incorporation into the benzenoid-derived 1-hydroxy-6-oxo-cyclohex-2-en-1-carboxylic acid moiety and in the case of homaloside D and tremulacin/6-*O*′-benzoylsalicortin into the benzoic acid moiety as well (Table 2). Altogether, these results indicate that cinnamic acid acts as a common precursor in the formation of volatile and nonvolatile benzenoids in *P. trichocarpa* leaves.

**Table 2:** Incorporation of D_7_-cinnamic acid (D_7_-CA) into salicinoids of *Chrysomela populi-*damaged *P. trichocarpa* leaves

Compound	H_2_O control (%)	D_7_-CA feeding (%)
Salicin	100 ± 0.0	94.8 ± 1.1
D_4_-salicin	ND	5.2 ± 1.1
Salicin-7-sulfate	100 ± 0.0	98.3 ± 0.3
D_4_-salicin-7-sulfate	ND	1.7 ± 0.3
Salirepin	100 ± 0.0	98.6 ± 0.3
D_3_-salirepin	ND	1.4 ± 0.3
Salirepin-7-sulfate	99.8 ± 0.005	99.4 ± 0.1
D_3_-salirepin-7-sulfate	0.2 ± 0.005	0.6 ± 0.1
Salicortin	100 ± 0.0	96.8 ± 0.7
D_8_-salicortin	ND	3.2 ± 0.7
Homaloside D	100 ± 0.0	99.7 ± 0.1
D_12_-homaloside D	ND	0.3 ± 0.1
Tremulacin/6-*O*′-benzoylsalicortin	100 ± 0.0	98.7 ± 0.3
D_13_-tremulacin/6-*O*′-benzoylsalicortin	ND	1.3 ± 0.3

Compounds were extracted with methanol from freeze-dried plant material and analyzed using LC-MS/MS. The amount of each compound, labeled and unlabeled, is given as the percentage of the total amount of that compound. Means and SE (*n* = 3–4) are given.

### Identification of putative β-oxidative pathway genes for conversion of cinnamic acid to benzoyl-CoA

Essential for the formation of benzenoid specialized metabolites from cinnamic acid are the shortening of the propyl side chain by two carbons (Widhalm and Dudareva, 2015). Such chain shortening has been described in petunia flowers and found to be catalyzed by a three-step enzymatic β-oxidative pathway, which is localized in the peroxisomes (Van Moerkercke et al., 2009; Klempien et al., 2012; Qualley et al., 2012). These three enzymatic steps are catalyzed by cinnamate-CoA ligase (CNL), cinnamoyl-CoA hydratase/dehydrogenase (CHD), and 3-ketoacyl-CoA thiolase (KAT), respectively (Van Moerkercke et al., 2009; Klempien et al., 2012; Qualley et al., 2012). Based on our in planta labeling results, we hypothesized that a similar chain shortening pathway might also exist in poplars. Thus, we aimed to identify putative candidate genes by performing Basic Local Alignment Search Tool (BLAST) analyses with the already described *PhCNL*, *PhCHD*, and *PhKAT* genes from petunia (Van Moerkercke et al., 2009; Klempien et al., 2012; Qualley et al., 2012) and the *P. trichocarpa* genome as template. Using *PhCNL* as BLAST query, we identified seven putative *P. trichocarpa CNL* (*PtCNL*) candidate genes *Potri.017G138400*, *Potri.T116500*, *Potri.T116700*, *Potri.004G082000*, *Potri.016G034800*, *Potri.006G036200*, and *Potri.006G036300*, which were designated as *PtCNL1*–*7*, respectively (Supplemental Figure S1; Shi et al., 2010). Detailed sequence comparisons showed that *PtCNL1* and *PtCNL2* were identical, and thus we further refer only to *PtCNL1*. An amino acid alignment of PtCNL1–7 with the characterized CNLs from petunia (PhCNL; Klempien et al., 2012) and Arabidopsis (AtBZO1, *Arabidopsis thaliana*; Lee et al., 2012) revealed the existence of an AMP-binding site, a characteristic motif for acyl-activating enzymes, in the poplar CNL candidates (Supplemental Figure S2; Shockey and Browse, 2011). Moreover, all poplar CNLs except for PtCNL3 contain a C-terminal peroxisomal targeting sequence I (Supplemental Figure S2 and Table S6). A BLAST search of the *P. trichocarpa* genome with *PhCHD* revealed three putative *P. trichocarpa CHD* candidate genes, which were designated as *PtCHD1* (*Potri.018G082900*), *PtCHD2* (*Potri.010G011900*), and *PtCHD3* (*Potri.008G220400*). Further sequence analysis and amino acid alignments enabled the identification of conserved motifs responsible for the isomerase/hydratase and the dehydrogenase activity, which are essential for bifunctional CHD enzymes (Supplemental Figure S3; Arent et al., 2010). In analogy to the CNLs, all putative poplar CHD enzymes possess a peroxisomal targeting sequence I motif (Supplemental Figure S3 and Table S7). Finally, by using *PhKAT* as BLAST template, we identified three putative *KAT* candidate genes *PtKAT1* (*Potri.002G216400*), *PtKAT2* (*Potri.001G051900*), and *PtKAT3* (*Potri.001G051800*) in the *P. trichocarpa* genome. Sequence analysis and amino acid alignments showed that the three candidate enzymes contain conserved thiolase sequence motifs and a peroxisomal targeting sequence II (Supplemental Figure S4 and Table S7; Reumann, 2004; Van Moerkercke et al., 2009).

### Heterologous expression of poplar CNL, CHD, and KAT enzymes in *E. coli*

**Figure 3:**
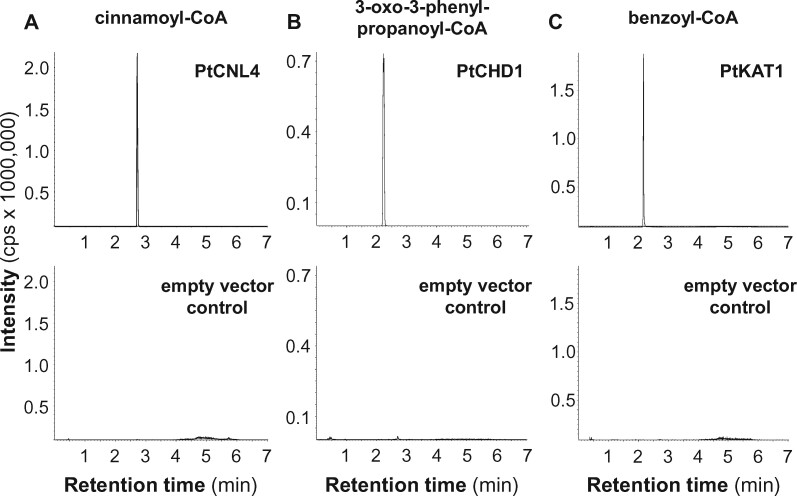
Enzymatic activity of *Populus trichocarpa* cinnamate-CoA ligase 4 (PtCNL4), cinnamoyl-CoA hydratase/dehydrogenase 1 (PtCHD1), and 3-ketoacyl-CoA thiolase 1 (PtKAT1). *PtCNL4* (A), *PtCHD1* (B), and *PtKAT1* (C) were heterologously expressed in *E. coli* as His_6_-tag fusion proteins and recombinant proteins were purified using affinity chromatography. Purified proteins were incubated with the potential substrates *trans*-cinnamic acid (PtCNL4), cinnamoyl-CoA (PtCHD1), cinnamoyl-CoA (+ PtCHD1; PtKAT1) and the respective cosubstrates ATP, CoA (PtCNL4); NAD^+^ (PtCHD1); CoA, NAD^+^ (PtKAT1). Reaction products were analyzed using LC–MS/MS. cps, counts per second.

To test the enzymatic activity of the putative PtCNL, PtCHD, and PtKAT candidate enzymes, we amplified their complete open reading frames from cDNA of beetle-treated *P. trichocarpa* leaves and inserted them into an expression vector. Notably, the amplification of *PtCNL3* only yielded truncated versions of *PtCNL1*, indicating that *PtCNL3* is not expressed in beetle-damaged *P. trichocarpa* leaves. The cloned genes were heterologously expressed in *Escherichia coli* and purified N-terminal His_6_-Tag fusion proteins were assayed with different substrates. The formation of enzyme products was analyzed using LC-MS/MS. For testing the enzyme activity of putative CNL candidates, different aromatic carboxylic acids were chosen as potential substrates. The most efficient substrates used by all PtCNL enzymes except PtCNL6 were *trans*-cinnamic acid, 2-hydroxycinnamic acid, and 4-hydroxycinnamic acid, whereas caffeic acid, ferulic acid, and benzoic acid were converted in substantially lower amounts (Figure 3; Supplemental Table S8 and Figure S5). The highest enzymatic activities were measurable for PtCNL1 and PtCNL4, which activated almost all of the supplied *trans*-cinnamic acid and 2-hydroxycinnamic acid into the respective CoA esters (Supplemental Table S8). In contrast, PtCNL6 possessed only trace activity with all of the tested substrates (Supplemental Table S8).

The enzymatic activities of the putative PtCHD candidate enzymes were tested with cinnamoyl-CoA, 2-hydroxycinnamoyl-CoA, and 4-hydroxycinnamoyl-CoA as substrates. Although all three CHD candidates accepted these aromatic CoA esters as substrates in vitro, they showed different catalytic efficiencies (Figure 3; Supplemental Table S9 and Figure S5). While PtCHD1 showed the highest enzymatic activity, PtCHD2 and PtCHD3 possessed only minor enzymatic activities with the tested substrates (Supplemental Table S9 and Figure S5). LC-MS/MS analysis of enzyme assays containing the putative poplar KAT enzymes, PtCHD1, and different aromatic CoA esters as CHD substrates revealed that PtKAT1 and PtKAT2 efficiently catalyzed the shortening of the propyl side chain of 3-oxo-3-phenylpropanoyl-CoA and 3-oxo-3-(4-hydroxy)-phenylpropanoyl-CoA in vitro (Figure 3; Supplemental Tables S10 and S11 and Figure S5). PtKAT3, however, showed only trace activity with 3-oxo-3-phenylpropanoyl-CoA as substrate, and no activity with other tested substrates in vitro (Supplemental Tables S10 and S11 and Figure S5).

### Genes encoding the β-oxidative pathway are upregulated upon herbivory

**Figure 4:**
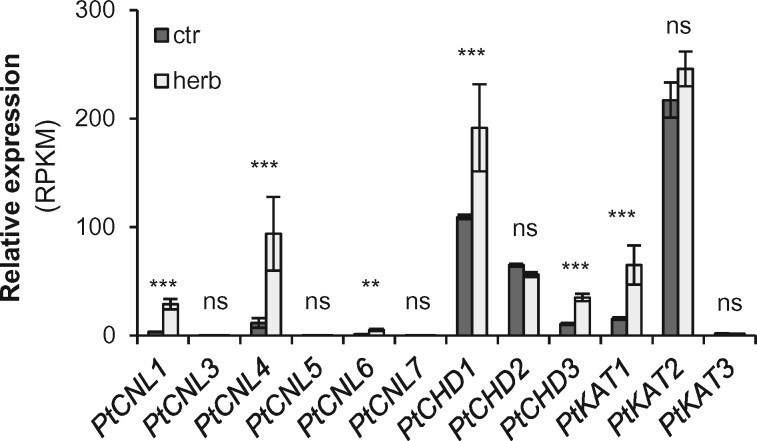
Expression of putative poplar *CNL*, *CHD*, and *KAT* genes in *Chrysomela populi*-damaged (herb) and undamaged (ctr) *Populus trichocarpa* leaves*.* Absolute normalized expression values were obtained from transcriptomes (*n* = 4 biological replicates). RPKM, reads per kilo base per million mapped reads. Significant differences in EDGE tests are visualized by asterisks. Means ± se are shown. *PtCNL1* (*P *=* *2.38E−07, WD = 1.71E−05); *PtCNL3* (*P *=* *1.0, WD = 1.2E−07); *PtCNL4* (*P *=* *1.53E−11, WD = 1.31E−04); *PtCNL5* (*P *=* *1.0, WD = −1.36E−08); *PtCNL6* (*P *=* *2.5E−03, WD = 6.5E−06); *PtCNL7* (*P *=* *1.0, WD = 1.53E−07); *PtCHD1* (*P *=* *1.33E−04, WD = 1.30E−04); *PtCHD2* (*P *=* *1.60E−01, WD = −1.47E−05); *PtCHD3* (*P *=* *3.04E−11, WD = 3.89E−05); *PtKAT1* (*P *=* *2.76E−11, WD = 7.94E−05); *PtKAT2* (*P *=* *1.88E−01, WD = 4.38E−05); *PtKAT3* (*P *=* *1.0, WD = −3.67E−07).

To investigate the expression of putative β-oxidative pathway genes, we sequenced and compared the transcriptomes of *C. populi*-damaged and undamaged *P. trichocarpa* leaves. While the expression of *PtCNL1*, *PtCNL4*, *PtCNL6*, *PtCHD1*, *PtCHD3*, and *PtKAT1* was significantly upregulated in response to herbivory, the expression of all other genes was not influenced by the treatment (Figure 4). Comparing the fold changes, *PtCNL1* and *PtCNL4* showed the highest induction with a 7.5- and 7.8-fold increase upon herbivory, respectively. The induction of *PtCHD1*, *PtCHD3*, and *PtKAT1* gene expression was less pronounced with a 1.7-, 3.2-, and 4.2-fold increase in herbivore-treated leaves versus undamaged leaves, respectively. To verify the obtained RNAseq data, we performed reverse transcription-quantitative PCR (RT-qPCR) analysis, which confirmed the observed expression pattern (Supplemental Figure S6). However, we could not obtain primers specific for *PtCNL3*, but used instead a primer pair amplifying both *PtCNL1* and *PtCNL3*, covering a region with high sequence polymorphisms. Sequencing of the cloned *PtCNL1/PtCNL3* RT-qPCR products revealed no transcripts of *PtCNL3*, confirming that this gene is not expressed in poplar leaves.

### RNAi-mediated knockdown of *CNL1 and 4* led to a decreased emission of benzenoid volatiles upon herbivory, but did not alter the accumulation of salicinoids

**Figure 5:**
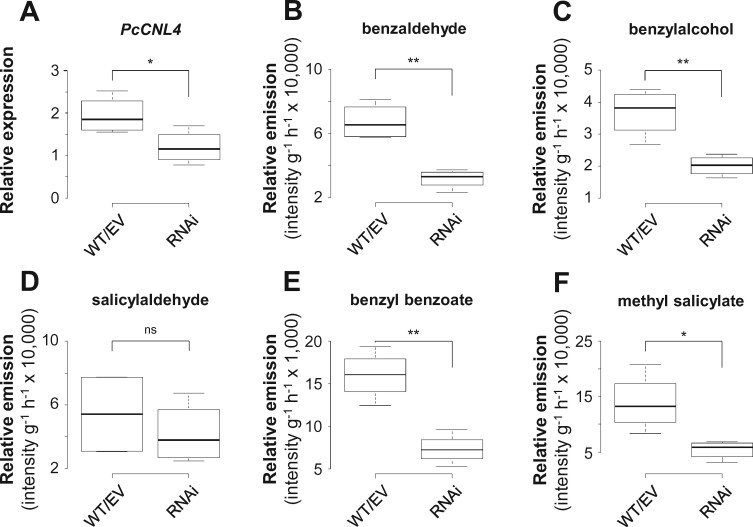
Effect of RNAi-mediated knockdown of the *CNL1* and *4* in *Populus* x *canescens* on the emission of aromatic compounds. Transcript accumulation of *P.* x *canescens CNL4* (A), and the relative emission of benzaldehyde (B), benzylalcohol (C), salicylaldehyde (D), benzyl benzoate (E), and methyl salicylate (F) of *Chrysomela populi*-damaged wild-type (WT) and transgenic *P.* x *canescens* trees are shown. Gene expression was measured using RT-qPCR, with *ubiquitin* (Ramírez-Carvajal et al., 2008) as housekeeping gene. Volatiles were analyzed using GC-MS. Medians ± quartiles, and outliers are shown (*n* = 4 biological replicates). EV, empty vector; ns, not significant. Asterisks indicate statistical significance as assed by Student's *t* test (ST); *P <* *0.05; **P <* *0.01). *PcCNL4* (*P *=* *0.045, *t* = −2.519, ST); benzaldehyde (*P *=* *0.002, *t* = −5.491, ST); benzylalcohol (*P *=* *0.007, *t* = −4.080, ST); salicylaldehyde (*P *=* *0.492, *t* = −0.731, ST); benzyl benzoate (*P *=* *0.002, *t* = −5.123, ST); methyl salicylate (*P *=* *0.020, *t* = −3.131, ST).

**Figure 6:**
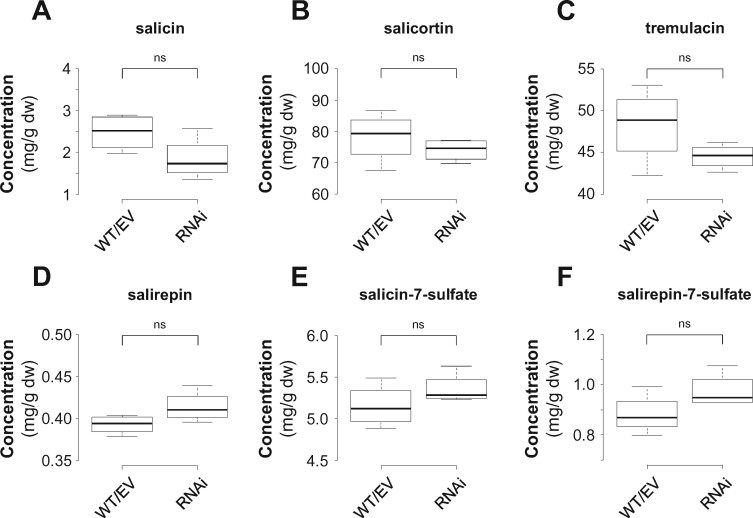
Effect of RNAi-mediated knockdown of the *CNL1* and *4* in *Populus* x *canescens* on the formation of salicinoids. Concentration of salicin (A), salicortin (B), tremulacin (C), salirepin (D), salicin-7-sulfate (E), and salirepin-7-sulfate (F) in leaves of *Chrysomela populi*-damaged wild-type (WT) and transgenic *P.* x *canescens* trees are shown. Compounds were extracted with methanol from freeze-dried leaf material and analyzed using LC–MS/MS and HPLC-UV. Medians ± quartiles, and outliers are shown (*n* = 4 biological replicates). EV, empty vector; dw, dry weight; ns, not significant. Differences between WT/EV and RNAi knockdown lines were analyzed by Student's *t* test (ST). Salicin (*P *=* *0.111, *t* = −1.865, ST); salicortin (*P *=* *0.385, *t* = −0.936, ST); tremulacin (*P *=* *0.168, *t* = −1.566, ST); salirepin (*P *=* *0.098, *t* = 1.961, ST); salicin-7-sulfate (*P *=* *0.242, *t* = 1.298, ST); salirepin-7-sulfate (*P *=* *0.131, *t* = 1.748, ST).

Biochemical characterization of candidate enzymes and gene expression analysis suggested that the β-oxidative pathway might contribute to the formation of herbivore-induced aromatic compounds in poplar (Figures 3 and 4). To test this hypothesis in planta, we performed RNA interference (RNAi)-mediated knockdown of *CNL* genes in gray poplar (*P*. x *canescens*), the poplar species usually used for *Agrobacterium*-mediated transformation (Leple et al., 1992). Since only *PtCNL1* and *PtCNL4* showed considerable gene expression in beetle-damaged *P. trichocarpa* leaves (Figure 4), we aimed to generate RNAi lines with reduced *CNL1* and *CNL4* gene expression by transforming *P.* x *canescens* with a DNA fragment complementary to *CNL1* and *4*. Four independent *CNL1* and *4* knockdown lines (RNAi-1, RNAi-2, RNAi-3, and RNAi-4) were generated and compared to empty vector (EV) trees and wild-type (WT) trees. Gene expression analysis by RT-qPCR showed that *CNL4* and *CNL1* were significantly less expressed in *C. populi*-damaged leaves of the knockdown lines compared to WT trees and EV trees (Figure 5; Supplemental Figures S7 and S8). The expression of *CNL6* was not altered, and expression of *CNL5* and *CNL7* was not detectable (Supplemental Figures S7 and S8). GC-MS analysis of volatiles collected from *C. populi*-damaged leaves revealed that the emission of benzaldehyde, benzylalcohol, benzyl benzoate, and methyl salicylate was significantly reduced in the *CNL1* and *4* knockdown trees in comparison to EV trees and WT plants (Figure 5; Supplemental Figure S9). Notably, the herbivore-induced emission of salicylaldehyde, the N-containing compounds, and most of the volatile terpenoids was not affected by the downregulation of *CNL1* and *4* (Figure 5; Supplemental Figure S9 and Tables S12 and S13). In addition, the accumulation of volatile benzenoids in leaf tissue was not significantly reduced in the RNAi knockdown lines compared with WT and EV trees, with the exception of benzyl benzoate (Supplemental Tables S14 and S15). In addition to volatile benzenoids, we measured the concentration of nonvolatile salicinoids and aromatic carboxylic acids in the *P.* x *canescens* leaf tissue by HPLC-UV and LC-MS/MS, respectively. Analysis of the salicinoid content revealed no significant differences between *P.* x *canescens CNL* knockdown trees, EV trees, and WT trees (Figure 6; Supplemental Figure S10). Moreover, the accumulation of benzoic acid derivatives and phenylpropanoid carboxylic acids, except 2-hydroxycinnamic acid, was also not influenced by the downregulation of *CNL1* and *4* (Supplemental Tables S16–S19).

## Discussion

Benzenoids (C_6_–C_1_ aromatic compounds) are an important class of aromatic specialized metabolites that occur widely in the plant kingdom. Poplars and willows, for instance, are known to produce a variety of volatile and nonvolatile benzenoids that contribute to their defense. However, the biosynthesis of benzenoid compounds is largely unknown. Thus, we aimed to investigate the pathway of benzenoid formation in *P. trichocarpa* and here report that a peroxisomal β-oxidative pathway mediates the formation of benzoyl-CoA, a universal C_6_–C_1_ building block of volatile and nonvolatile benzenoids.

### Volatile benzenoids are produced via the β-oxidative pathway

**Figure 7:**
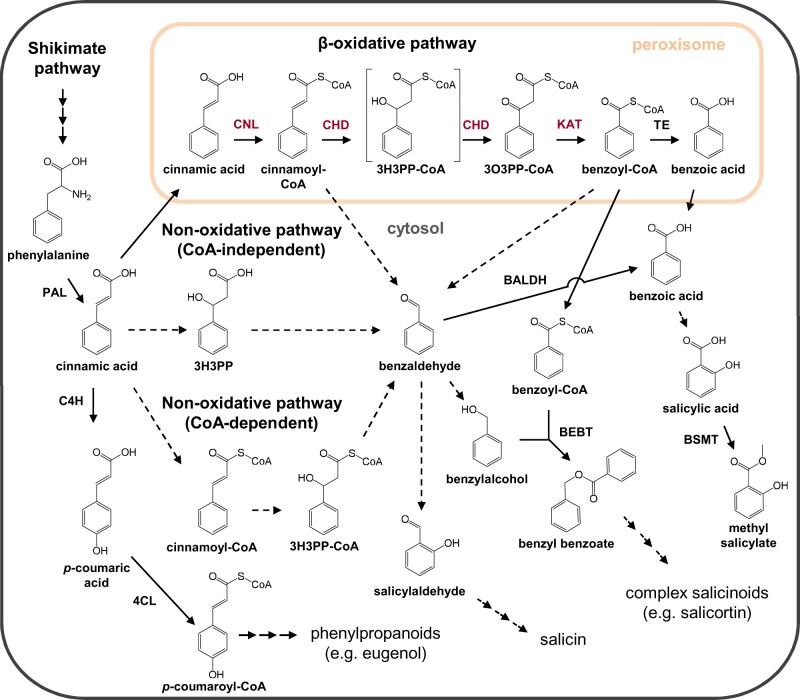
Schematic overview of benzenoid (C_6_–C_1_) biosynthetic pathways in plants. Displayed are the peroxisomal β-oxidative pathway investigated and two proposed nonoxidative cytosolic pathways in the biosynthesis of benzenoid compounds from the common precursor cinnamic acid. Already known enzymatic steps from plants are displayed with solid arrows, unknown enzymatic steps with dashed arrows. Enzymes investigated in this study are highlighted in red, already characterized enzymes from other plants are displayed in black. BALDH, benzaldehyde dehydrogenase; BEBT, benzoyl-CoA:benzyl alcohol O-benzoyltransferase; BSMT, benzoic acid/salicylic acid methyltransferase; 4CL, 4-coumarate CoA-ligase; CoA, Coenzyme A; 3H3PP, 3-hydroxy-3-phenylpropionic acid; 3H3PP-CoA, 3-hydroxy-3-phenylpropanoyl-CoA; 3O3PP, 3-oxo-3-phenylpropanoyl-CoA; PAL, phenylalanine ammonia lyase; TE, thioesterase.

Volatile benzenoids are typical components of floral and vegetative plant volatile bouquets (Knudsen et al., 2006; Dudareva et al., 2013; Widhalm and Dudareva, 2015; Amrad et al., 2016; Sas et al., 2016). We showed that feeding of the poplar leaf beetle *C. populi* on *P. trichocarpa* leaves resulted in a significantly induced emission of different benzenoids, for example, benzaldehyde, benzylalcohol, salicylaldehyde, and benzyl benzoate (Figure 1; Supplemental Table S1). Although volatile benzenoids are typically emitted from herbivore-treated poplar leaves (Figure 1; Danner et al., 2011; McCormick et al., 2014; Fabisch et al., 2019), their biosynthesis has remained elusive. In general, cinnamic acid is assumed as the common biosynthetic precursor of most benzenoid compounds (Figure 7; Widhalm and Dudareva, 2015). Indeed, feeding experiments with deuterium-labeled D_7_-cinnamic acid revealed the incorporation of cinnamic acid into volatile benzenoids in poplar (Figure 2). Essential for the further formation of benzenoids from cinnamic acid is the shortening of the propyl side chain by two carbons (Widhalm and Dudareva, 2015). A peroxisome-localized three-step β-oxidative pathway that contributes to the formation of benzenoid specialized metabolites has been described in petunia and in Arabidopsis (Van Moerkercke et al., 2009; Colquhoun et al., 2012; Klempien et al., 2012; Lee et al., 2012; Qualley et al., 2012; Bussell et al., 2014). This pathway involves the sequential action of a CNL, a CHD, and a KAT; Van Moerkercke et al., 2009; Klempien et al., 2012; Qualley et al., 2012). By performing sequence and transcriptome analysis, we identified genes in the *P. trichocarpa* genome encoding CNL, CHD, and KAT enzymes that are likely localized in the peroxisomes. Heterologous expression and biochemical characterization revealed activities and substrate specificities consistent with a peroxisomal β-oxidative pathway (Figures 3 and 7; Supplemental Tables S8–S11). Gene expression analysis showed that the seven identified *CNL* genes were barely expressed in undamaged leaves and only two of them (*PtCNL1* and *PtCNL4*) showed considerable expression levels after herbivore feeding (Figure 4). In contrast, two out of the three identified *CHDs* (*PtCHD1* and *PtCHD2*) and one out of the three identified *KATs* (*PtKAT2*) were highly expressed in undamaged leaves and only moderately induced (*PtCHD1*) or even not induced (*PtCHD2*, *PtKAT2*) upon herbivory (Figure 4). These data indicate that the β-oxidative pathway in poplar peroxisomes is mainly controlled by the expression of *PtCNL4* and *PtCNL1* and thus mainly contributes to the formation of benzenoids upon herbivory. Indeed, RNAi-mediated knockdown of *CNL1* and *4* in *P.* x *canescens*, a poplar species closely related to *P. trichocarpa*, resulted in the reduced emission of herbivore-induced benzenoid volatiles, whereas the formation of constitutively accumulating salicinoids was not influenced (Figures 5–7).

The end product of the β-oxidative pathway is the first C_6_–C_1_ intermediate, benzoyl-CoA, which can serve as precursor for different metabolic pathways. In petunia, a peroxisomal-localized thioesterase has recently been described to catalyze the hydrolysis of benzoyl-CoA to benzoic acid (Adebesin et al., 2018). Moreover, it has been proposed that benzoyl-CoA might be transported out of the peroxisomes into the cytosol via so far unknown transporters (Figure 7; Adebesin et al., 2018). In the cytosol, benzoyl-CoA can act as substrate for BAHD-acyltransferases that produce aromatic esters (D'Auria, 2006). Recently, an acyltransferase has been identified in poplar that is able to catalyze the formation of benzyl benzoate using benzoyl-CoA as substrate (Figure 7; Chedgy et al., 2015). It is also feasible that, as in the lignin biosynthesis, benzoyl-CoA is directly converted to benzaldehyde by the activity of a cinnamoyl-CoA reductase (Chao et al., 2017; Liu et al., 2018). The benzaldehyde produced might function as a key intermediate in the formation of other benzenoids (Figure 7). Short-chain dehydrogenases could for instance catalyze its reduction to benzylalcohol (Kavanagh et al., 2008), while benzaldehyde dehydrogenases as for example described in snapdragon (*Antirrhinum majus*) convert it to benzoic acid (Figure 7; Long et al., 2009). Benzoic acid, either produced through putative hydrolases or by oxidation of benzaldehyde, might act as substrate for a 2-hydroxylase catalyzing the formation of salicylic acid, which can then be further methylated to form volatile methyl salicylate (Figure 7; e.g. Negre et al., 2002; Widhalm and Dudareva, 2015).

### Alternative chain shortening pathways likely contribute to the formation of nonvolatile benzenoids in poplar

Poplar trees are known to produce a variety of nonvolatile benzenoids, including the highly abundant and constitutively produced salicinoids (Böckler et al., 2011, 2013; Feistel et al., 2015; Fabisch et al., 2019). Although the formation of salicinoids is poorly understood, inhibition and labeling studies proposed cinnamic acid as common precursor for the benzenoid core structure (Zenk, 1967; Ruuhola and Julkunen-Tiitto, 2003; Babst et al., 2010). Our in planta feeding experiments with deuterium-labeled D_7_-cinnamic acid support this hypothesis, as we confirmed its incorporation into the core benzenoid 2-(hydroxymethyl)phenol moiety as well as into the benzenoid-related 1-hydroxy-6-oxo-cyclohex-2-en-1-carboxylic acid moiety of complex salicinoids (Table 2). Although shortening of the propyl side chain of cinnamic acid is essential for the formation of salicinoids, none of the identified *CNL* candidates showed a constitutive and high expression pattern consistent with salicinoid accumulation. Moreover, the RNAi-mediated knockdown of *CNL1* and *4* resulted in a significantly reduced emission of herbivore-induced benzenoid volatiles, but had no effect on the accumulation of salicinoids or other nonvolatile benzenoids (Figure 6; Supplemental Tables S12–S19). This indicates that, apart from the β-oxidative pathway, another chain shortening pathway should exist in poplar that contributes to the formation of nonvolatile benzenoids. Two cytosolic nonoxidative chain shortening pathways have been postulated in the literature, which are either CoA-dependent or CoA-independent (Widhalm and Dudareva, 2015). As part of the CoA-dependent nonoxidative pathway, cinnamic acid could be first activated to cinnamoyl-CoA and further hydrated to 3-hydroxy-3-phenylpropanoyl-CoA (Figure 7; Widhalm and Dudareva, 2015). An enoyl-CoA hydratase/lyase might then catalyze the chain shortening of 3-hydroxy-3-phenylpropanoyl-CoA, leading to the formation of benzaldehyde (Figure 7; Widhalm and Dudareva, 2015). Although an enoyl-CoA hydratase/lyase has been described in *Pseudomonas fluorescens* (Gasson et al., 1998), comparable evidence in plants is scarce, as the expected enzymatic activity has been only observed in crude protein extracts of sweet amber (*Hypericum androsaemum*) cell cultures (Abd El-Mawla and Beerhues, 2002). The CoA-independent pathway first comprises the hydration of cinnamic acid to 3-hydroxy-3-phenylpropionic acid and its further reverse aldol cleavage to benzaldehyde (Figure 7; Widhalm and Dudareva, 2015). This enzymatic activity has been mainly observed in crude protein extracts from different plant species such as tobacco (*Nicotiana tabacum*), Asian pear (*Pyrus pyrifolia*), and apple (*Malus domestica*; Malinowski et al., 2007; Sarkate et al., 2018; Saini et al., 2019). Molecular and biochemical evidences for a responsible gene have been published by Gallage et al. (2014), who described a vanilin synthase that converts ferulic acid to vanilin. However, the actual activity of this enzyme is controversial (Yang et al., 2017). Metabolic flux analysis and isotope labeling experiments revealed that both the nonoxidative CoA-independent pathway and the β-oxidative pathway contribute to the formation of benzenoids in petunia flowers (Boatright et al., 2004; Orlova et al., 2006). Thus, it is conceivable that at least one of the cytosolic pathways might also exist in poplar and contribute to the formation of nonvolatile benzenoids.

### Benzenoid specialized metabolites might have multiple functions in poplar defense against herbivores

The emission of volatiles is an important trait in the communication of sessile plants with their environment (Unsicker et al., 2009; Dudareva et al., 2013; Muhlemann et al., 2014; Amrad et al., 2016; Sas et al., 2016). Herbivory, for instance, is often described to induce the emission of volatiles, which contributes to the direct and indirect defense responses of the plant (Unsicker et al., 2009). In the current study, we revealed that herbivory of the specialist *C. populi* on *P. trichocarpa* leaves results in the induced formation of benzenoid volatiles for example benzaldehyde or benzyl benzoate (Figure 1; Supplemental Table S1). Although benzenoid volatiles are in general characteristic components of the herbivore-induced volatile blend of poplar trees (Figure 1; Supplemental Table S1; Danner et al., 2011; McCormick et al., 2014; Fabisch et al., 2019), their ecological functions in the plant defense response are poorly understood. Only a few studies have been conducted so far revealing the importance of benzenoid volatiles in plant–environment interactions. Behavioral assays with generalist-feeding *L. dispar* caterpillars, for example, showed that volatile benzaldehyde acts as repellent (Eberl et al., 2018). Moreover, benzyl benzoate emitted from petunia flowers has been shown to deter multiple florivores, including the cucumber beetle *Diabrotica undecimpunctata* and the tree cricket *Oecanthus fultoni* (Kessler et al., 2013). Thus, it is tempting to speculate that benzenoids emitted from poplar might also influence the feeding behavior of *C. populi* beetles.

Salicinoids are well-known as effective defense compounds against various herbivores, including coleopteran insects (e.g. Tahvanainen et al., 1985; Lindroth et al., 1988; Lindroth and Peterson, 1988; Kelly and Curry, 1991; Bailey et al., 2006). They are mostly constitutively accumulating defense metabolites and their formation is assumed to be more dependent on the genotype, the developmental stage, or the season, then on induction by herbivore feeding (Böckler et al., 2011; 2013; Fabisch et al., 2019). Our study showed that the concentrations of the complex salicinoids salicortin, homaloside D, tremulacin, and 6-*O*′-benzoylsalicortin were not influenced by *C. populi* herbivory (Table 1). However, the concentration of the simplest salicinoid salicin significantly increased in response to the herbivore treatment (Table 1). Whereas the effectiveness of complex salicinoids as defense compounds is well-known (Böckler et al., 2011), the available data about the effectiveness of salicin are contradictory (Lindroth et al., 1988; Lindroth and Peterson, 1988; Böckler et al., 2016). Only a slight negative impact of salicin has been described in defense against the tiger swallowtail (*Papilio glaucus glaucus*) and the southern armyworm (*Spodoptera* *eridania*; Lindroth et al., 1988; Lindroth and Peterson, 1988). Moreover, food choice assays with artificially applied salicin revealed only a marginal influence on the feeding preference of *L. dispar* caterpillars (Lackus et al., 2020). Similarly, the increased salicin content might not serve as a defense against the specialist leaf herbivore *C. populi*. On the contrary, *C. populi* larvae use salicin for their own defense against predators and microbial infection by sequestration and further metabolism to salicylaldehyde (Pasteels et al., 1983; Gross et al., 2002; Michalski et al., 2008; Burse et al., 2009) and adult beetles might sequester salicin as well (Pasteels et al., 1982).

## Materials and methods

### Plant and insect material

Black cottonwood (*P. trichocarpa*, clone Muhle-Larsen, P&P Baumschule, Eitelborn, Germany) trees were propagated from monoclonal stem cuttings and grown under summer conditions in the greenhouse (day, 23–25°C; night, 19–23°C; 50%–60% rel. humidity; 16-h/8-h light/dark cycle) in a 1:1 mixture of sand and clay granules (Klasmann-Deilmann, Geeste, Germany), until they reached about 80–100 cm in height. Gray poplar (*Populus* x *canescens* clone INRA 717-1B4) trees were propagated and cultivated as described in the section “Vector construction and transformation of poplar”. *P.* x *canescens* trees were used at an age of 7 weeks, corresponding to a height of ∼60 cm. Poplar leaf beetles (*Chrysomela populi*) originated from egg clutches from old-growth black poplar (*Populus nigra*) trees collected in the field (Fabisch et al., 2019) and were further propagated and cultivated on *P. nigra* trees in the laboratory.

### Herbivore treatment

Leaves were numbered according to the leaf plastochron index (LPI; Irmisch et al., 2013). Before the onset of the experiment, *C. populi* beetles were starved for 13 h. For the herbivore treatment, 12 *C. populi* beetles were released on leaves LPI3–7 and were allowed to feed for 24 h (8 am–8 am). On the second day, beetles were removed from the plants to avoid volatile contamination and leaves were enclosed in new PET bags (Bratschlauch, Toppits, Minden, Germany). Volatile collection was then performed from 10 a.m. to 4 p.m. as described in the section “Volatile collection and analysis”. For analysis of *P.* x *canescens* WT trees, EV trees, and *CNL* knockdown lines, LPI4 was enclosed in a PET bag and four *C. populi* beetles (starved for 13 h) were allowed to feed for 24 h (8 a.m.–8 a.m.). After removal of the beetles and the enclosing of the leaf in a new PET bag, the volatile collection was performed on the second day, from 10 a.m. to 4 p.m. as described in the section “Volatile collection and analysis”. After the volatile collection, leaf material was harvested, flash-frozen in liquid nitrogen, ground, and one part stored at −80°C until further processing, while the other part was lyophilized and stored at 4°C.

### Feeding experiment with D_7_-cinnamic acid

Single leaves (LPI3) from *P. trichocarpa* trees were cut and incubated overnight (4 p.m.–8 a.m.) in tap water or a solution containing 2 mM D_7_-cinnamic acid (Sigma Aldrich, St. Louis, MO, USA). Two *C. populi* beetles per leaf, starved for 13 h before the onset of the experiment, were then allowed to feed for 24 h (8 a.m.–8 a.m.) on the *P. trichocarpa* leaves. On the second day, beetles were removed, leaves were enclosed in new PET bags (Bratschlauch, Toppits), and volatile collection was performed from 10 a.m. to 4 p.m. as described in the section “Volatile collection and analysis”. Afterward, leaf material was harvested, flash-frozen in liquid nitrogen, ground, and one part stored at −80°C until further processing, while the other part was lyophilized and stored at 4°C.

### Volatile collection and analysis

Volatiles were collected using a dynamic push–pull system. Air flow was maintained in the system through Teflon tubes. Charcoal filtered air was pumped into the bags at a flow of 1 L min^−1^. A portion of the aspirated air (flow: 0.6 L min^−1^) was withdrawn with a second pump and passed through a filter packed with 30 mg Poropak (ARS, Inc., Gainesville, FL, USA) to adsorb the volatile compounds. Volatiles were collected for 6 h during the middle of the light period of Day 2 after herbivore release (10 a.m.–4 p.m.). After the collection, the volatile compounds were desorbed by eluting the filter with 200 µL dichloromethane containing nonyl acetate as an internal standard (10 ng µL^−1^). Samples were stored at −20°C until gas chromatography analysis. Volatile collection of *P.* x *canescens* WT trees, EV trees, and *CNL* knockdown plants was conducted with an air supply of 0.8 L min^−1^ and an air withdrawal of 0.4 L min^−1^.

Qualitative and quantitative analyses of leaf volatiles were conducted using an Agilent 6890 Series gas chromatograph coupled to an Agilent 5973 quadrupole mass selective detector (Agilent Technologies, Santa Clara, CA, USA; interface temperature, 250°C; quadrupole temperature, 150°C; source temperature, 230°C; electron energy, 70 eV) or a flame ionization detector (FID) operated at 300°C, respectively. The constituents of the volatile bouquet were separated using a ZB5 column (Phenomenex, Aschaffenburg, Germany; 30 m × 0.25 mm × 0.25 µm) and He (MS) or H_2_ (FID) as carrier gas. The sample (1 µL or 2 µL) was injected without split at an initial oven temperature of 45°C. The temperature was held for 2 min and then increased to 180°C with a gradient of 6°C min^−1^, and then further increased to 300°C with a gradient of 60°C min^−1^ and hold of 2 min. Compounds were identified by comparison of retention times and mass spectra to those of authentic standards, or by comparison with reference spectra in the Wiley and National Institute of Standards and Technology libraries.

### Hexane extraction of plant material

To determine the accumulation of nonpolar compounds in poplar leaf tissue, 100 mg of ground leaf material was extracted in a GC glass vial with 400-µL hexane including 10-ng/µL nonyl acetate as an internal standard. The extracts were shaken for 1 h at 900 rpm and incubated overnight at room temperature. After centrifugation for 10 min at 5,000 *g*, the supernatant was taken and subsequently analyzed via GC-MS and GC-FID as described in the section “Volatile collection and analysis”.

### Methanol extraction of plant material

Metabolites were extracted from 10 mg freeze-dried plant material by adding 1 mL 100% methanol (MeOH) containing 0.8 mg/mL phenyl-β-d-glucopyranoside (Sigma Aldrich), 40 ng/mL D_6_-abscisic acid (D_6_-ABA), 40 ng/mL D_6_-jasmonic acid (D_6_-JA), D_4_-salicylic acid, and 8 ng/mL D_6_-jasmonic-acid isoleucine (D_6_-JA-Ile; Santa Cruz Biotechnology, Dallas, TX, USA) as internal standards. Samples were shaken for 30 s in a paint shaker (Scandex, Büdelsdorf, Germany) and afterward for 30 min at 200 rpm on a horizontal shaker (IKA Labortechnik, Staufen, Germany). After centrifugation, the supernatants were split for HPLC-UV and LC-MS/MS measurements and were subsequently analyzed. Extraction of nonlabeled and labeled leaf material was conducted as described above, except that pure 100% MeOH without internal standards was used.

### Liquid chromatography mass-spectrometry analysis of plant methanol extracts and enzyme products

*Targeted analysis for quantification.* Chromatographic separation was achieved using an Agilent 1260 infinity II LC system (Agilent Technologies, Santa Clara, CA, USA) equipped with a Zorbax Eclipse XDB-C18 column (50 × 4.6 mm, 1.8 μm, Agilent Technologies), using aqueous formic acid (0.05% (v/v)) and acetonitrile as mobile phases A and B, respectively. The mobile phase flow rate was 1.1 mL/min. The elution profile is listed in Supplemental Table S20 as gradient A. The column temperature was maintained at 20°C. The LC system was coupled to a QTRAP 6500 tandem mass spectrometer (Sciex, Darmstadt, Germany) equipped with a turbospray ion source, operated in negative ionization mode. The ion spray voltage was maintained at −4,500 eV and the turbo gas temperature was set at 700°C. Nebulizing gas was set at 60 psi, curtain gas at 40 psi, heating gas at 60 psi, and collision gas at medium level. Multiple reaction monitoring (MRM) was used to monitor analyte parent ion → product ion formation for each analyte as displayed in Supplemental Table S21. The analysis of phenolic acids in *P.* x *canescens* leaf material and enzyme assays with PtCNL1/4–7 was conducted using the same analytical parameters and equipment as listed above, but using aqueous acetic acid (0.1% (v/v)) and acetonitrile as mobile phases A and B, respectively. The mobile phase flow rate was 1.1 mL min^−1^. The elution profile used is listed in Supplemental Table S20 as gradient B. Identification and quantification of compounds were performed using standard curves made from authentic standards. Quantification of sulfated salicinoids and salirepin was conducted as described previously in Lackus et al. (2020) and phytohormone quantification was conducted as described previously in Irmisch et al. (2014). In brief, an Agilent 1260 infinity II LC system (Agilent Technologies) coupled to a QTRAP 6500 tandem mass spectrometer (Sciex) was used for the analysis. Chromatographic separation was achieved using a Zorbax Eclipse XDB-C18 column (50 × 4.6 mm, 1.8 μm, Agilent Technologies), and aqueous formic acid (0.05% (v/v)) and acetonitrile as mobile phases A and B, respectively. The mobile phase flow rate was 1.1 mL/min. The elution profile is listed in Supplemental Table S20 as gradient C. The tandem mass spectrometer was equipped with a turbospray ion source, operated in negative ionization mode. The ion spray voltage was maintained at −4,500 eV and the turbo gas temperature was set at 650°C. Nebulizing gas was set at 70 psi, curtain gas at 40 psi, heating gas at 70 psi, and collision gas at medium level. MRM was used to monitor analyte parent ion → product ion formation for each analyte as displayed in Supplemental Table S21. Data acquisition and processing was performed using Analyst 1.6.3 (Sciex) and MultiQuant 3.0.3 (Sciex) software.

*Targeted analysis for label incorporation.* The analysis of *P. trichocarpa* leaf material concerning the incorporation of D_7_-cinnamic acid was conducted using the same analytical parameters and equipment as described in the previous section “Targeted analysis for quantification”, but MRM for labeled compounds were added as displayed in Supplemental Table S21. MRM for nonlabeled compounds were used as described in the section “Targeted analysis for quantification” (Supplemental Table S21), except that for each compound the DP value was set at −60 V.

*Analysis of enzyme products.* Products of CNL, CHD, and KAT enzymes were measured using an Agilent 1260 infinity II LC system (Agilent Technologies, Santa Clara, CA, USA), coupled to a QTRAP 6500 tandem mass spectrometer (Sciex, Darmstadt, Germany) equipped with a turbospray ion source, operated in negative ionization mode. The ion spray voltage was maintained at −4,500 eV and the turbo gas temperature was set at 650°C. Nebulizing gas was set at 60 psi, curtain gas at 40 psi, heating gas at 60 psi, and collision gas at medium level. The chromatographic separation was conducted using a Zorbax Eclipse XDB-C18 column (50 × 4.6 mm, 1.8 μm, Agilent Technologies), and aqueous ammonium acetate (20 mM (v/v) (A)) and acetonitrile (B) as mobile phases. The elution profile is displayed in Supplemental Table S20 as gradient D, and gradient E. The flow rate was 1.1 ml min^−1^, and the column temperature was maintained at 20°C. The MRM mode was used to monitor precursor ion → product ion reactions for each analyte as listed in Supplemental Table S21. Data acquisition and processing were performed using Analyst 1.6.3 (Sciex) and MultiQuant 3.0.3 (Sciex) software.

### HPLC-UV analysis for salicinoid quantification

Salicinoid content was analyzed and quantified by HPLC-UV (200 nm) as described previously in Böckler et al. (2013) for the compounds salicin, salicortin, tremulacin, and homaloside D. The 6′-*O*-benzoylsalicortin content was analyzed and quantified by HPLC-UV (200 nm) as described in Lackner et al. (2019).

### RNA extraction and reverse transcription

Total RNA was isolated from frozen and ground plant material using the InviTrap Spin Plant RNA Kit (Invitek, Berlin, Germany) according to manufacturer’s instructions. RNA concentration was assessed using a spectrophotometer (NanoDrop 2000c, Thermo Fisher Scientific, Waltham, MA, USA). RNA was treated with DNaseI (Thermo Fisher Scientific) before cDNA synthesis. Single-stranded cDNA was prepared from 1 µg of DNase-treated RNA using SuperScript III reverse transcriptase and oligo (dT_12–18_) primers (Invitrogen, Carlsbad, CA, USA).

### Identification and cloning of putative poplar *CNL*, *CHD*, and *KAT* genes

Putative poplar *CNL*, *CHD*, and *KAT* genes were identified by BLAST analysis using *PhCNL* (Klempien et al., 2012), *PhCHD* (Qualley et al., 2012), and *PhKAT1* (Van Moerkercke et al., 2009) from petunia (*Petunia hybrida*) as the respective queries, and the poplar genome as reference (Tuskan et al., 2006; http://www.phytozome.net/poplar). The complete open reading frames of the candidate genes, *Potri.017G138400* (*PtCNL1*), *Potri.004G082000* (*PtCNL4*), *Potri.016G034800* (*PtCNL5*), *Potri.006G036200* (*PtCNL6*), *Potri.006G036300* (*PtCNL7*), *Potri.018G082900* (*PtCHD1*), *Potri.010G011900* (*PtCHD2*), *Potri.008G220400* (*PtCHD3*), *Potri.002G216400* (*PtKAT1*), *Potri.001G051900* (*PtKAT2*), and *Potri.001G051800* (*PtKAT3*), were amplified from leaf cDNA, inserted into the *E. coli* expression vector pET100/D-TOPO (Thermo Fisher Scientific), and cloned genes were fully sequenced (Supplemental Table S22). *Potri.T116700* (*PtCNL3*) could not be amplified and cloned.

### Phylogenetic analysis and amino acid alignment

An alignment of putative *P. trichocarpa CNL* genes and characterized *CNL* genes from Arabidopsis (*A. thaliana*) and petunia was constructed using the MUSCLE (codon) algorithm (gap open, −2.9; gap extend, 0; hydrophobicity multiplier, 1.5; clustering method, UPGMB) implemented in MEGA6 (Tamura et al., 2013). Tree reconstruction was done with MEGA6 using a Maximum Likelihood algorithm (model/method, Tamura 3-parameter model; substitutions type, nucleotide; rates among sites, uniform rates; gaps/missing data treatment, partial deletion; site coverage cutoff, 80%). A bootstrap resampling analysis with 1,000 replicates was performed to evaluate the tree topology. Amino acid alignments of putative poplar CNL, CHD, and KAT enzymes, together with the already characterized Arabidopsis and petunia enzymes were constructed with MEGA6 and visualized with BioEdit (http://www.mbio.ncsu.edu/bioedit/bioedit.html).

### RNAseq and RT-qPCR analysis

Total RNA was extracted from leaf material as described above, TruSeqRNA-compatible libraries were prepared, and PolyA enrichment was performed before sequencing the transcriptomes of four control and four herbivore-treated *P. trichocarpa* leaves on an IlluminaHiSeq 2500 sequencer (Max Planck Genome Centre, Cologne, Germany) with 18 Mio reads per library, 100 bp, single end. Trimming of the obtained Illumina reads and mapping to the poplar gene model version 3.0 (https://phytozome.jgi.doe.gov/pz/portal.html) were performed with the program CLC Genomics Workbench (Qiagen Bioinformatics, Venlo, Netherlands; mapping parameter: length fraction, 0.7; similarity fraction, 0.9; maximum number of hits, 25). Empirical analysis of digital gene expression (EDGE) implemented in the program CLC Genomics Workbench was used for gene expression analysis.

For RT-qPCR analysis, cDNA was prepared as described above and diluted 1:10 with water. For the amplification of *CNL*, *CHD*, and *KAT* gene fragments, primers were designed having a *T*_m_ ≥ 60°C, a GC content between 50% and 60%, and a primer length of ∼20 nucleotides (Supplemental Table S23). The specificity of the primers was confirmed by agarose gel electrophoresis, melting curve analysis, standard curve analysis, and sequence verification of cloned PCR amplicons. *Ubiquitin (UBQ)*, *actin*, *elongation factor 1 alpha* (*EF1α*), *histone superfamily protein H3 (HIS)*, and *tubulin* (*TUB*) were tested as reference genes (Ramírez-Carvajal et al., 2008; Xu et al., 2011; Wang et al., 2014). Comparison of ΔCq values and the corresponding standard deviation revealed *TUB* as most valid reference gene for expression analysis in *P. trichocarpa* samples (Supplemental Table S24). Based on previous studies from Irmisch et al. (2013) and Günther et al. (2019), *UBQ* was used as reference gene for expression analysis in transgenic and nontransgenic *P.* x *canescens* leaves. Gene expression analysis was performed with an initial incubation at 95°C for 3 min followed by 40 cycles of amplification (95°C for 10 s, 60°C for 10 s). For all measurements, plate reads were taken at the end of the extension step of each cycle, and data for the melting curves were recorded at the end of cycling from 60°C to 95°C. All samples were run on the same PCR machine (Bio-Rad CFX Connect Real-Time PCR Detection System (Bio-Rad Laboratory, Hercules, CA, USA)) in an optical 96-well plate, using Brilliant III SYBR Green QPCR Master Mix (Stratagene, San Diego, CA, USA). For analyzing gene expression in *P. trichocarpa*, four biological replicates were analyzed, each as technical triplicates. Expression analysis of the *P.* x *canescens* knockdown trees, WT trees, and EV trees was conducted for all biological replicates in technical triplicates.

### Heterologous expression of *CNL, CHD*, and *KAT* genes

The *E. coli* strain BL21 Star™ (DE3; Thermo Fisher Scientific) was used for heterologous expression of *Potri.017G138400* (*PtCNL1*), *Potri.004G082000* (*PtCNL4*), *Potri.016G034800* (*PtCNL5*), *Potri.006G036200* (*PtCNL6*), *Potri.006G036300* (*PtCNL7*), *Potri.018G082900* (*PtCHD1*), *Potri.010G011900* (*PtCHD2*), *Potri.008G220400* (*PtCHD3*), *Potri.002G216400* (*PtKAT1*), *Potri.001G051900* (*PtKAT2*), and *Potri.001G051800* (*PtKAT3*). Cultures were grown at 37°C, induced at an OD_600_ = 0.6 with 1 mM IPTG, subsequently placed at 18°C, and grown for another 18 h. The cells were collected by centrifugation and resuspended in chilled extraction buffer (100 mM potassium phosphate, 150 mM KCl, 10% glycerol (v/v), 20 mM imidazole, 300 mM NaCl, 0.5 mg/mL lysozyme, 10 U/mL Benzonase Nuclease (Merck), 7 mM β-mercaptoethanol, protease-inhibitor mix HP (according to manufactures instruction, Serva Electrophoresis GmbH, Heidelberg, Germany), pH 7.5). After 30 min incubation on ice, the cells were disrupted by five cycles of freezing the samples in liquid N and thawing them in a water bath at 25°C. Afterward, the samples were centrifuged for 30 min at 4°C with 15,000*g* (2 times), and the supernatant was used for further affinity-based purification with the HisPur Cobalt Resin (Thermo Fisher Scientific), following the manufacturer’s instructions. Purified proteins were desalted and concentrated via Amicon Ultra-0.5 Centrifugal Filter Devices (Merck Millipore, Merck KGaA, Darmstadt, Germany), according to manufacturer’s instruction, into a storage buffer (pH 7.5) containing 20 mM potassium phosphate and 10% glycerol (v/v). Protein concentration was determined by the Bradford method (Bradford, 1976), using the Quick Start^TM^ Bradford 1× Dye Reagent (Bio-Rad Laboratory, Hercules, CA, USA). To determine the catalytic activity of the recombinant enzymes, the following enzyme assays were performed; CNL: 1.5 µg purified protein, 0.2 mM substrate (*trans*-cinnamic acid, 2-hydroxycinnamic acid, 4-hydroxycinnamic acid, caffeic acid, ferulic acid, benzoic acid), 2.5 mM ATP, 2.5 mM MgCl_2_, 50 mM KCl, 2 mM CoA in a total volume of 100 µL; CHD: 3 µg purified protein, 0.2 mM substrate (cinnamoyl-CoA (TransMIT GmbH, Gießen, Germany); 2-hydroxycinnamoyl-CoA (TransMIT GmbH); 4-hydroxycinnamoyl-CoA (MicroCombiChem, Wiesbaden, Germany), 2.5 mM MgCl_2_, 1 mM pyruvic acid, 1 mM NAD^+^, 2 mM CoA, 2 U lactate dehydrogenase in a total volume of 100 µl. All assays were incubated for 1 h at 25°C under shaking at 300 rpm, and stopped by the addition of equal volumes of methanol. The determination of the relative enzymatic activity was conducted in technical triplicates. The enzymatic activity of poplar KAT enzymes was determined in combined assays with PtCHD1 as follows: 1 µg purified protein PtKAT, 5 µg purified protein PtCHD1, 0.2 mM substrate (cinnamoyl-CoA; 2-hydroxycinnamoyl-CoA; 4-hydroxycinnamoyl-CoA), 25 mM MgCl_2_, 1 mM pyruvic acid, 1 mM NAD^+^, 2 mM CoA, 2 U lactate dehydrogenase, 50 mM KCl in a final volume of 150 µL. All assays were incubated for 1 h at 25°C under shaking at 300 rpm, only containing the purified PtCHD1 protein. After the first incubation, the respective PtKAT enzyme was added to the enzyme assays and incubated again for 1 h at 25°C under shaking at 300 rpm. The reactions were stopped by the addition of equal volumes of methanol and all assays were conducted in technical triplicates. Enzyme assays were analyzed via LC-MS/MS as described in the sections “Analysis of enzyme products” and “Targeted analysis for quantification”.

### Vector construction and transformation of poplar

The construction of the binary vector was performed as described by Levée et al. (2009). The transformation of the *P.* × *canescens* clone INRA 7171-B4 followed a protocol published by Meilan and Ma (2007). To target *CNL1* and *4* mRNA together, a fragment between position 1286 and 1412 of the coding sequence of *PtCNL1* and *4* was selected. Transgenic RNAi plants were amplified by micropropagation as described by Behnke et al. (2007). Saplings of ∼10 cm high were repotted to soil (Klasmann potting substrate) and propagated in a controlled environment chamber for around six weeks (day, 22°C; night, 18°C; 65% relative humidity; 16-h/8-h light/dark cycle) before they were transferred to the greenhouse. To test the level of gene silencing, RT-qPCR analysis was done on WT plants, EV control plants, and RNAi plants.

### Statistical analyses

Throughout the article, data are presented as means ± standard errors (se). Statistical analysis was done with SigmaPlot 11.0 for Windows (Systat Software Inc.). The statistical analyses performed are mentioned in the figure and table legends for the respective experiments. Student’s *t* tests (STs) were conducted to determine statistical significant differences between control and herbivore-treated *P. trichocarpa* leaves. Whenever necessary, the data were log-transformed to meet statistical assumptions such as normality and homogeneity of variances. In case that the statistical assumptions could not be fulfilled, nonparametric Mann–Whitney (MW) Rank Sum Test analyses were conducted. The statistical analyses performed are displayed for each compound in the respective figures and tables in parentheses (ST or MW). Four independent RNAi-knockdown lines (RNAi 1–4), three independent EV lines (EV 1–3), and one WT line were generated (biological replicates). Through the propagation of monoclonal stem cuttings, 3–6 technical replicates of each line have been generated and analyzed. The collected data for the technical replicates have been averaged to obtain the data for each biological replicate. Data are displayed as means ± se for the technical and biological replicates. ST analyses, or in case that statistical assumptions could not be fulfilled, nonparametric MW Rank Sum Test analyses have been performed as two-group comparison between the RNAi and the WT/EV trees (biological replicates, *n* = 4). The statistical analyses performed are given for each compound in the respective figures, and tables in parentheses (ST or MW). For analysis and quantification of volatile and nonvolatile herbivore-induced compounds from *P.* x *canescens* trees, the level of herbivory was included. Transcriptome analysis has been conducted by using the EDGE implemented in the program CLC Genomics Workbench (QIAGEN). The EDGE test was performed as a two-group comparison (Robinson and Smyth, 2007) as part of the edgeR Bioconductor package (Robinson et al. 2010). The default settings of the EDGE test have been used for the transcriptome analysis as implemented in the CLC Genomics Workbench program (https://digitalinsights.qiagen.com/).

### Accession numbers

Sequence data for *PtCNL1* (MT952989), *PtCNL4* (MT952990), *PtCNL5* (MT952991), *PtCNL6* (MT952992), *PtCNL7* (MT952982), *PtCHD1* (MT952986), *PtCHD2* (MT952987), *PtCHD3* (MT952988), *PtKAT1* (MT952983), *PtKAT2* (MT952984), and *PtKAT3* (MT952985) can be found in the NCBI GenBank (https://www.ncbi.nlm.nih.gov/genbank/) under the corresponding identifiers. Raw reads of the RNAseq experiment were deposited in the NCBI Sequence Read Archive (SRA) under the BioProject accession PRJNA660618.

### Data availability

All supporting data are included as additional files. Constructs described in this work and datasets analyzed during the current study are available from the corresponding author upon request. Sequences were deposited in GenBank (see “Materials and Methods” section).

## Supplemental Data

The following materials are available in the online version of this article.

**Su** **pplemental Figure S1**. Cladogram analysis of putative poplar *CNL* genes, characterized poplar *4-coumarate-CoA ligase* (*4CL*) genes, and characterized *CNL* genes from other plants.

**Supplemental Figure S2**. Amino acid sequence comparison of putative *Populus trichocarpa* CNL Potri.017G138400 (PtCNL1), Potri.T116700 (PtCNL3), Potri.004G082000 (PtCNL4), Potri.016G034800 (PtCNL5), Potri.006G036200 (PtCNL6), and Potri.006G036300 (PtCNL7) with the characterized CNL from *Petunia hybrida* (Ph) and *A. thaliana* (AtBZO1).

**Supplemental Figure S3**. Amino acid sequence comparison of putative *Populus trichocarpa* cinnamoyl-CoA hydratases/dehydrogenases (CHD) Potri.018G082900 (PtCHD1), Potri.010G011900 (PtCHD2), and Potri.008G220400 (PtCHD3) with the characterized CHD from *Petunia hybrida* (Ph).

**Supplemental Figure S4**. Amino acid sequence comparison of putative *Populus trichocarpa* 3-ketoacyl-CoA thiolases (KAT) Potri.002G216400 (PtKAT1), Potri.001G051900 (PtKAT2), and Potri.001G051800 (PtKAT3) with the characterized KAT1 from *Petunia hybrida* (Ph).

**Supplemental Figure S5**. Enzymatic activity of *Populus trichocarpa* cinnamate-CoA ligases (PtCNL), cinnamoyl-CoA hydratase/dehydrogenases (PtCHD), and 3-ketoacyl-CoA thiolases (PtKAT).

**Supplemental Figure S6**. Gene expression analysis of poplar *CNL*, *CHD*, and *KAT*.

**Supplemental Figure S7**. RNAi-mediated knockdown of the *CNL1* and *4* in *Populus* x *canescens*.

**Supplemental Figure S8**. RNAi-mediated knockdown of the *CNL1* and *4* in *Populus* x *canescens*.

**Supplemental Figure S9**. Effect of RNAi-mediated knockdown of the *CNL1* and *4* in *Populus* x *canescens* on the emission of aromatic compounds.

**Supplemental Figure S10**. Effect of RNAi-mediated knockdown of the *CNL1* and *4* in *Populus* x *canescens* on the formation of salicinoids.

**Supplemental Table S1**. Volatiles (ng g^−1^ h^−1^ fw) emitted from undamaged (control) and *Chrysomela populi-*damaged (herbivory) *Populus trichocarpa* leaves.

**Supplemental Table S2**. Content of nonpolar compounds (µg/g fresh weight) in undamaged (control) and *Chrysomela populi-*damaged (herbivory) *Populus trichocarpa* leaves.

**Supplemental Table S3**. Aromatic amino acid and aromatic carboxylic acid content (µg/g dry weight) in undamaged (control) and *Chrysomela populi-*damaged (herbivory) *Populus trichocarpa* leaves.

**Supplemental Table S4**. Phytohormone content (ng/g dry weight) in undamaged (control) and *Chrysomela populi-*damaged (herbivory) *Populus trichocarpa* leaves.

**Supplemental Table S5**. Incorporation of D_7_-cinnamic acid (D_7_-CA) into aromatic carboxylic acids in *Chrysomela populi-*damaged *Populus trichocarpa* leaves.

**Supplemental Table S6**. Predicted subcellular localization of poplar cinnamate-CoA ligases (PtCNL).

**Supplemental Table S7**. Predicted subcellular localization of poplar cinnamoyl-CoA hydratases/dehydrogenases (PtCHD) and 3-ketoacyl-CoA thiolases (PtKAT).

**Supplemental Table S8**. Enzymatic activity of *Populus trichocarpa* cinnamate-CoA ligases (PtCNL) assayed with different substrates.

**Supplemental Table S9**. Enzymatic activity of *Populus trichocarpa* cinnamoyl-CoA hydratases/dehydrogenases (PtCHD) assayed with different substrates.

**Supplemental Table S10**. Enzymatic activity of *Populus trichocarpa* 3-ketoacyl-CoA thiolases (PtKAT) assayed with different substrates.

**Supplemental Table S11**. Enzymatic activity of *Populus trichocarpa* 3-ketoacyl-CoA thiolases (PtKAT) assayed with different substrates.

**Supplemental Table S12**. Volatiles (ng g^−1^ h^−1^ fresh weight) emitted from *Chrysomela populi-*damaged *Populus* x *canescens* leaves of *CNL1* and *4* knockdown (RNAi), EV, and WT trees

**Supplemental Table S13**. Volatiles (ng g^−1^ h^−1^ fresh weight) emitted from *Chrysomela populi-*damaged *Populus* x *canescens* leaves of *CNL1* and *4* knockdown (RNAi), EV, and WT trees.

**Supplemental Table S14**. Content of nonpolar benzenoid compounds (µg/g fresh weight) in *Chrysomela populi-*damaged *Populus* x *canescens* leaves of *CNL1* and *4* knockdown (RNAi), EV, and WT trees.

**Supplemental Table S15**. Content of nonpolar benzenoid compounds (µg/g fresh weight) in *Chrysomela populi-*damaged *Populus* x *canescens* leaves of *CNL1* and *4* knockdown (RNAi), EV, and WT trees.

**Supplemental Table S16**. Benzenoid carboxylic acid content (µg/g dry weight) in *Chrysomela populi-*damaged *Populus* x *canescens* leaves of *CNL1* and *4* knockdown (RNAi), EV, and WT trees.

**Supplemental Table S17**. Benzenoid carboxylic acid content (µg/g dry weight) in *Chrysomela populi-*damaged *Populus* x *canescens* leaves of *CNL1* and *4* knockdown (RNAi), EV, and WT trees.

**Supplemental Table S18**. Phenylpropanoid carboxylic acid content (µg/g dry weight) in *Chrysomela populi-*damaged *Populus* x *canescens* leaves of *CNL1* and *4* knockdown (RNAi), EV, and WT trees.

**Supplemental Table S19**. Phenylpropanoid carboxylic acid content (µg/g dry weight) in *Chrysomela populi-*damaged *Populus* x *canescens* leaves of *CNL1* and *4* knockdown (RNAi), EV, and WT trees.

**Supplemental Table S20**. HPLC gradients used for separation and analysis of metabolites.

**Supplemental Table S21**. Parameters used for LC-MS/MS analysis.

**Supplemental Table S22**. Oligonucleotides used for the amplification of full-length *Populus trichocarpa* (Pt) *CNL*, *CHD*, and *KAT*.

**Supplemental Table S23**. Oligonucleotides used for gene expression analysis of *Populus trichocarpa* (Pt) *CNL*, *CHD*, and *KAT* by RT-qPCR.

**Supplemental Table S24**. Expression levels of potential housekeeping genes in undamaged (control) and *Chrysomela populi-damaged* (herbivory) *Populus trichocarpa* leaves.

## Supplementary Material

kiab111_Supplementary_Data

## Notes

N.D.L., J.G., and T.G.K. designed the research. N.D.L. and A.S. carried out the experimental work. N.D.L., A.S., and T.G.K. analyzed data. N.D.L. and T.G.K. wrote the manuscript. All authors read and approved the final manuscript.

The author responsible for distribution of materials integral to the findings presented in this article in accordance with the policy described in the Instructions for Authors (https://academic.oup.com/plphys/pages/general-instructions) is: T.G.K. (koellner@ice.mpg.de).

## References

[kiab111-B1] Abd El-Mawla AM , Beerhues L (2002) Benzoic acid biosynthesis in cell cultures of *Hypericum androsaemum*. Planta 214: 727–733 11882941 10.1007/s004250100657

[kiab111-B2] Adebesin F , Widhalm JR, Lynch JH, McCoy RM, Dudareva N (2018) A peroxisomal thioesterase plays auxiliary roles in plant β-oxidative benzoic acid metabolism. Plant J 93: 905–916 29315918 10.1111/tpj.13818

[kiab111-B3] Amrad A , Moser M, Mandel T, de Vries M, Schuurink RC, Freitas L, Kuhlemeier C (2016) Gain and loss of floral scent production through changes in structural genes during pollinator-mediated speciation. Curr Biol 26: 3303–3312 27916524 10.1016/j.cub.2016.10.023

[kiab111-B4] Arent S , Christensen CE, Pye VE, Norgaard A, Henriksen A (2010) The multifunctional protein in peroxisomal β-oxidation: structure and substrate specificity of the *Arabidopsis thaliana* protein MFP2. J Biol Chem 285: 24066–24077 20463021 10.1074/jbc.M110.106005 PMC2911295

[kiab111-B5] Babst BA , Harding SA, Tsai C-J (2010) Biosynthesis of phenolic glycosides from phenylpropanoid and benzenoid precursors in *Populus*. J Chem Ecol 36: 286–297 20177744 10.1007/s10886-010-9757-7

[kiab111-B6] Bailey JK , Schweitzer JA, Rehill BJ, Irschick DJ, Whitham TG, Lindroth RL (2006) Rapid shifts in the chemical composition of aspen forests: an introduced herbivore as an agent of natural selection. Biol Invasions 9: 715–722

[kiab111-B7] Behnke K , Ehlting B, Teuber M, Bauerfeind M, Louis S, Hansch R, Polle A, Bohlmann J, Schnitzler JP (2007) Transgenic, non-isoprene emitting poplars don't like it hot. Plant J 51: 485–499 17587235 10.1111/j.1365-313X.2007.03157.x

[kiab111-B8] Boatright J , Negre F, Chen X, Kish CM, Wood B, Peel G, Orlova I, Gang D, Rhodes D, Dudareva N (2004) Understanding *in vivo* benzenoid metabolism in petunia petal tissue. Plant Physiol 135: 1993–2011 15286288 10.1104/pp.104.045468 PMC520771

[kiab111-B9] Böckler GA , Gershenzon J, Unsicker SB (2011) Phenolic glycosides of the Salicaceae and their role as anti-herbivore defenses. Phytochemistry 72: 1497–1509 21376356 10.1016/j.phytochem.2011.01.038

[kiab111-B10] Böckler GA , Gershenzon J, Unsicker SB (2013) Gypsy moth caterpillar feeding has only a marginal impact on phenolic compounds in old-growth black poplar. J Chem Ecol 39: 1301–1312 24154955 10.1007/s10886-013-0350-8

[kiab111-B11] Böckler GA , Paetz C, Feibicke P, Gershenzon J, Unsicker SB (2016) Metabolism of poplar salicinoids by the generalist herbivore *Lymantria dispar* (Lepidoptera). Insect Biochem Mol Biol 78: 39–49 27503687 10.1016/j.ibmb.2016.08.001

[kiab111-B12] Bradford MM (1976) A rapid and sensitive method for the quantitation of microgram quantities of protein utilizing the principle of protein-dye binding. Anal Biochem 72: 248–254 942051 10.1016/0003-2697(76)90527-3

[kiab111-B13] Burse A , Frick S, Discher S, Tolzin-Banasch K, Kirsch R, Strauss A, Kunert M, Boland W (2009) Always being well prepared for defense: the production of deterrents by juvenile *Chrysomelina* beetles (Chrysomelidae). Phytochemistry 70: 1899–1909 19733867 10.1016/j.phytochem.2009.08.002

[kiab111-B14] Bussell JD , Reichelt M, Wiszniewski AA, Gershenzon J, Smith SM (2014) Peroxisomal ATP-binding cassette transporter COMATOSE and the multifunctional protein ABNORMAL INFLORESCENCE MERISTEM are required for the production of benzoylated metabolites in Arabidopsis seeds. Plant Physiol 164: 48–54 24254312 10.1104/pp.113.229807 PMC3875823

[kiab111-B15] Chao N , Li N, Qi Q, Li S, Lv T, Jiang X-N, Gai Y (2017) Characterization of the cinnamoyl-CoA reductase (CCR) gene family in *Populus tomentosa* reveals the enzymatic active sites and evolution of CCR. Planta 245: 61–75 27580618 10.1007/s00425-016-2591-6

[kiab111-B16] Chedgy RJ , Köllner TG, Constabel CP (2015) Functional characterization of two acyltransferases from *Populus trichocarpa* capable of synthesizing benzyl benzoate and salicyl benzoate, potential intermediates in salicinoid phenolic glycoside biosynthesis. Phytochemistry 113: 149–159 25561400 10.1016/j.phytochem.2014.10.018

[kiab111-B17] Colquhoun TA , Marciniak DM, Wedde AE, Kim JY, Schwieterman ML, Levin LA, Van Moerkercke A, Schuurink RC, Clark DG (2012) A peroxisomally localized acyl-activating enzyme is required for volatile benzenoid formation in a *Petunia* x *hybrida* cv ‘Mitchell Diploid’ flower. J Exp Bot 63: 4821–4833 22771854 10.1093/jxb/ers153 PMC3428004

[kiab111-B18] Danner H , Böckler GA, Irmisch S, Yuan JS, Chen F, Gershenzon J, Unsicker SB, Köllner TG (2011) Four terpene synthases produce major compounds of the gypsy moth feeding-induced volatile blend of *Populus trichocarpa*. Phytochemistry 72: 897–908 21492885 10.1016/j.phytochem.2011.03.014

[kiab111-B19] D'Auria JC (2006) Acyltransferases in plants: a good time to be BAHD. Curr Opin Plant Biol 9: 331–340 16616872 10.1016/j.pbi.2006.03.016

[kiab111-B20] Dudareva N , Klempien A, Muhlemann JK, Kaplan I (2013) Biosynthesis, function and metabolic engineering of plant volatile organic compounds. New Phytol 198: 16–32 23383981 10.1111/nph.12145

[kiab111-B21] Eberl F , Hammerbacher A, Gershenzon J, Unsicker SB (2018) Leaf rust infection reduces herbivore-induced volatile emission in black poplar and attracts a generalist herbivore. New Phytol 220: 760–772 28418581 10.1111/nph.14565

[kiab111-B22] Fabisch T , Gershenzon J, Unsicker SB (2019) Specificity of herbivore defense responses in a woody plant, black poplar (*Populus nigra*). J Chem Ecol 45: 162–177 30788656 10.1007/s10886-019-01050-y PMC6469625

[kiab111-B23] Feistel F , Paetz C, Lorenz S, Beran F, Kunert G, Schneider B (2017) *Idesia polycarpa* (Salicaceae) leaf constituents and their toxic effect on *Cerura vinula* and *Lymantria dispar* (Lepidoptera) larvae. Phytochemistry 143: 170–179 28822319 10.1016/j.phytochem.2017.08.008

[kiab111-B24] Feistel F , Paetz C, Lorenz S, Schneider B (2015) The absolute configuration of salicortin, HCH-salicortin and tremulacin from *Populus trichocarpa* x *deltoides* Beaupre. Molecules 20: 5566–5573 25830788 10.3390/molecules20045566 PMC6272461

[kiab111-B25] Fellenberg C , Corea O, Yan LH, Archinuk F, Piirtola EM, Gordon H, Reichelt M, Brandt W, Wulff J, Ehlting J, Constabel CP (2019) Discovery of salicyl benzoate UDP-glycosyltransferase, a central enzyme in poplar salicinoid phenolic glycoside biosynthesis. Plant J 102: 99–115 10.1111/tpj.14615 31736216

[kiab111-B26] Fürstenberg-Hägg J , Zagrobelny M, Bak S (2013) Plant defense against insect herbivores. Int J Mol Sci 14: 10242–10297 23681010 10.3390/ijms140510242 PMC3676838

[kiab111-B27] Gaid MM , Sircar D, Müller A, Beuerle T, Liu B, Ernst L, Hänsch R, Beerhues L (2012) Cinnamate:CoA ligase initiates the biosynthesis of a benzoate-derived xanthone phytoalexin in *Hypericum calycinum* cell cultures. Plant Physiol 160: 1267 22992510 10.1104/pp.112.204180 PMC3490583

[kiab111-B28] Gallage NJ , Hansen EH, Kannangara R, Olsen CE, Motawia MS, Jorgensen K, Holme I, Hebelstrup K, Grisoni M, Møller BL (2014) Vanillin formation from ferulic acid in *Vanilla planifolia* is catalysed by a single enzyme. Nat Commun 5: 4037 24941968 10.1038/ncomms5037 PMC4083428

[kiab111-B29] Gasson MJ , Kitamura Y, McLauchlan WR, Narbad A, Parr AJ, Parsons ELH, Payne J, Rhodes MJC, Walton NJ (1998) Metabolism of ferulic acid to vanillin: a bacterial gene of the enoyl-SCoA hydratase/isomerase superfamily encodes an enzyme for the hydration and cleavage of a hydroxycinnamic acid SCoA thioester. J Biol Chem 273: 4163–4170 9461612 10.1074/jbc.273.7.4163

[kiab111-B30] Gleadow RM , Møller BL (2014) Cyanogenic glycosides: synthesis, physiology, and phenotypic plasticity. Annu Rev Plant Biol 65: 155–185 24579992 10.1146/annurev-arplant-050213-040027

[kiab111-B31] Gross J , Podsiadlowski L, Hilker M (2002) Antimicrobial activity of exocrine glandular secretion of *Chrysomela* larvae. J Chem Ecol 28: 317–331 11925070 10.1023/a:1017934124650

[kiab111-B32] Günther J , Lackus ND, Schmidt A, Huber M, Stödler H-J, Reichelt M, Gershenzon J, Köllner TG (2019) Separate pathways contribute to the herbivore-induced formation of 2-phenylethanol in poplar. Plant Physiol 180: 767–782 30846485 10.1104/pp.19.00059 PMC6548255

[kiab111-B33] Heiska S , Tikkanen O-P, Rousi M, Julkunen-Tiitto R (2007) Bark salicylates and condensed tannins reduce vole browsing amongst cultivated dark-leaved willows (*Salix myrsinifolia*). Chemoecology 17: 245–253

[kiab111-B34] Irmisch S , Jiang Y, Chen F, Gershenzon J, Köllner TG (2014) Terpene synthases and their contribution to herbivore-induced volatile emission in western balsam poplar (*Populus trichocarpa*). BMC Plant Biol 14: 270 25303804 10.1186/s12870-014-0270-y PMC4197230

[kiab111-B35] Irmisch S , McCormick AC, Böckler GA, Schmidt A, Reichelt M, Schneider B, Block K, Schnitzler JP, Gershenzon J, Unsicker SB, et al (2013) Two herbivore-induced cytochrome P450 enzymes CYP79D6 and CYP79D7 catalyze the formation of volatile aldoximes involved in poplar defense. Plant Cell 25: 4737–4754 24220631 10.1105/tpc.113.118265 PMC3875747

[kiab111-B36] Jones P , Vogt T (2001) Glycosyltransferases in secondary plant metabolism: tranquilizers and stimulant controllers. Planta 213: 164–174 11469580 10.1007/s004250000492

[kiab111-B37] Kavanagh KL , Jornvall H, Persson B, Oppermann U (2008) Medium- and short-chain dehydrogenase/reductase gene and protein families: the SDR superfamily: functional and structural diversity within a family of metabolic and regulatory enzymes. Cell Mol Life Sci 65: 3895–3906 19011750 10.1007/s00018-008-8588-y PMC2792337

[kiab111-B38] Kelly MT , Curry JP (1991) The influence of phenolic compounds on the suitability of three *Salix* species as hosts for the willow beetle *Phratora vulgatissima*. Entomol Exp et Appl 61: 25–32

[kiab111-B39] Kessler D , Diezel C, Clark DG, Colquhoun TA, Baldwin IT (2013) Petunia flowers solve the defence/apparency dilemma of pollinator attraction by deploying complex floral blends. Ecol Lett 16: 299–306 23173705 10.1111/ele.12038

[kiab111-B40] Klempien A , Kaminaga Y, Qualley A, Nagegowda DA, Widhalm JR, Orlova I, Shasany AK, Taguchi G, Kish CM, Cooper BR, et al (2012) Contribution of CoA ligases to benzenoid biosynthesis in petunia flowers. Plant Cell 24: 2015–2030 22649270 10.1105/tpc.112.097519 PMC3442584

[kiab111-B41] Knudsen JT , Eriksson R, Gershenzon J, Ståhl B (2006) Diversity and distribution of floral scent. Bot Rev 72: 1

[kiab111-B42] Lackner S , Lackus ND, Paetz C, Köllner TG, Unsicker SB (2019) Aboveground phytochemical responses to belowground herbivory in poplar trees and the consequence for leaf herbivore preference. Plant Cell Environ 42: 3293–3307 31350910 10.1111/pce.13628

[kiab111-B43] Lackus ND , Müller A, Kröber TDU, Reichelt M, Schmidt A, Nakamura Y, Paetz C, Luck K, Lindroth RL, Constabel CP, et al (2020) The occurrence of sulfated salicinoids in poplar and their formation by sulfotransferase 1. Plant Physiol 183: 137–151 32098786 10.1104/pp.19.01447 PMC7210634

[kiab111-B44] Lee S , Kaminaga Y, Cooper B, Pichersky E, Dudareva N, Chapple C (2012) Benzoylation and sinapoylation of glucosinolate R-groups in Arabidopsis. Plant J 72: 411–422 22762247 10.1111/j.1365-313X.2012.05096.x

[kiab111-B45] Leple JC , Brasileiro ACM, Michel MF, Delmotte F, Jouanin L (1992) Transgenic poplars: expression of chimeric genes using four different constructs. Plant Cell Reports 11: 137–141 24213546 10.1007/BF00232166

[kiab111-B46] Levée V , Major I, Levasseur C, Tremblay L, MacKay J, Seguin A (2009) Expression profiling and functional analysis of *Populus* WRKY23 reveals a regulatory role in defense. New Phytol 184: 48–70 19674332 10.1111/j.1469-8137.2009.02955.x

[kiab111-B47] Lindroth RL , Peterson SS (1988) Effects of plant phenols of performance of southern armyworm larvae. Oecologia 75: 185–189 28310832 10.1007/BF00378595

[kiab111-B48] Lindroth RL , Scriber JM, Hsia MTS (1988) Chemical ecology of the tiger swallowtail: mediation of host use by phenolic glycosides. Ecology 69: 814–822

[kiab111-B49] Liu Q , Luo L, Zheng L (2018) Lignins: biosynthesis and biological functions in plants. Int J Mol Sci 19: 335 10.3390/ijms19020335 PMC5855557 29364145

[kiab111-B50] Long MC , Nagegowda DA, Kaminaga Y, Ho KK,, Kish CM, Schnepp J, Sherman D, Weiner H, Rhodes D, Dudareva N (2009) Involvement of snapdragon benzaldehyde dehydrogenase in benzoic acid biosynthesis. Plant J 59: 256–265 19292760 10.1111/j.1365-313X.2009.03864.x

[kiab111-B51] Maag D , Erb M, Köllner TG, Gershenzon J (2015) Defensive weapons and defense signals in plants: some metabolites serve both roles. Bioessays 37: 167–174 25389065 10.1002/bies.201400124

[kiab111-B52] MacDonald MJ , D'Cunha GB (2007) A modern view of phenylalanine ammonia lyase. Biochem Cell Biol 85: 273–282 17612622 10.1139/o07-018

[kiab111-B53] Malinowski J , Krzymowska M, Godoń K, Hennig J, Podstolski A (2007) A new catalytic activity from tobacco converting 2-coumaric acid to salicylic aldehyde. Physiol Plant 129: 461–471

[kiab111-B54] McCormick AC , Irmisch S, Reinecke A, Böckler GA, Veit D, Reichelt M, Hansson BS, Gershenzon J, Köllner TG, Unsicker SB (2014) Herbivore-induced volatile emission in black poplar: regulation and role in attracting herbivore enemies. Plant Cell Environ 37: 1909–1923 24471487 10.1111/pce.12287

[kiab111-B55] Meilan R , Ma C (2007) Poplar (*Populus spp.*). *In* Wang K, ed, Agrobacterium Protocols Volume 2. Humana Press, Totowa, NJ, pp 143–151

[kiab111-B56] Michalski C , Mohagheghi H, Nimtz M, Pasteels J, Ober D (2008) Salicyl alcohol oxidase of the chemical defense secretion of two chrysomelid leaf beetles: molecular and functional characterization of two new members of the glucose-methanol-choline oxidoreductase gene family. J Biol Chem 283: 19219–19228 18482980 10.1074/jbc.M802236200

[kiab111-B57] Mithöfer A , Boland W (2012) Plant defense against herbivores: chemical aspects. Annu Rev Plant Biol 63: 431–450 22404468 10.1146/annurev-arplant-042110-103854

[kiab111-B58] Muhlemann JK , Klempien A, Dudareva N (2014) Floral volatiles: from biosynthesis to function. Plant Cell Environ 37: 1936–1949 24588567 10.1111/pce.12314

[kiab111-B59] Negre F , Kolosova N, Knoll J, Kish CM, Dudareva N (2002) Novel S-adenosyl-l-methionine:salicylic acid carboxyl methyltransferase, an enzyme responsible for biosynthesis of methyl salicylate and methyl benzoate, is not involved in floral scent production in snapdragon flowers. Arch Biochem Biophys 406: 261–270 12361714 10.1016/s0003-9861(02)00458-7

[kiab111-B60] Orlova I , Marshall-Colon A,, Schnepp J, Wood B, Varbanova M, Fridman E, Blakeslee JJ, Peer WA, Murphy AS, Rhodes D, et al (2006) Reduction of benzenoid synthesis in petunia flowers reveals multiple pathways to benzoic acid and enhancement in auxin transport. Plant Cell 18: 3458–3475 17194766 10.1105/tpc.106.046227 PMC1785411

[kiab111-B61] Osier TL , Hwang S-Y, Lindroth RL (2000) Effects of phytochemical variation in quaking aspen *Populus tremuloides* clones on gypsy moth *Lymantria dispar* performance in the field and laboratory. Ecol Entomol 25: 197–207

[kiab111-B62] Pasteels JM , Braekman JC, Daloze D, Ottinger R (1982) Chemical defence in chrysomelid larvae and adults. Tetrahedron 38: 1891–1897

[kiab111-B63] Pasteels JM , Rowell-Rahier M, Braekman JC, Dupont A (1983) Salicin from host plant as precursor of salicylaldehyde in defensive secretion of *Chrysomeline* larvae. Physiol Entomol 8: 307–314

[kiab111-B64] Pichersky E , Lewinsohn E (2011) Convergent evolution in plant specialized metabolism. Annu Rev Plant Biol 62: 549–566 21275647 10.1146/annurev-arplant-042110-103814

[kiab111-B65] Qualley AV , Widhalm JR, Adebesin F, Kish CM, Dudareva N (2012) Completion of the core β-oxidative pathway of benzoic acid biosynthesis in plants. Proc Natl Acad Sci USA 109: 16383–16388 22988098 10.1073/pnas.1211001109 PMC3479573

[kiab111-B66] Ramírez-Carvajal GA, , Morse AM, Davis JM (2008) Transcript profiles of the cytokinin response regulator gene family in *Populus* imply diverse roles in plant development. New Phytol 177: 77–89 17944821 10.1111/j.1469-8137.2007.02240.x

[kiab111-B67] Reumann S (2004) Specification of the peroxisome targeting signals type 1 and type 2 of plant peroxisomes by bioinformatics analyses. Plant Physiol 135: 783–800 15208424 10.1104/pp.103.035584 PMC514115

[kiab111-B68] Robinson MD , McCarthy DJ, Smyth GK (2010) edgeR: a Bioconductor package for differential expression analysis of digital gene expression data. Bioinformatics 26: 139–140 19910308 10.1093/bioinformatics/btp616 PMC2796818

[kiab111-B69] Robinson MD , Smyth GK (2007) Small-sample estimation of negative binomial dispersion, with applications to SAGE data. Biostatistics 9: 321–332 17728317 10.1093/biostatistics/kxm030

[kiab111-B70] Ruuhola T , Julkunen-Tiitto R (2003) Trade-off between synthesis of salicylates and growth of micropropagated *Salix pentandra*. J Chem Ecol 29: 1565–1588 12921436 10.1023/a:1024266612585

[kiab111-B71] Saini SS , Teotia D, Gaid M, Sircar D (2019) A new enzymatic activity from elicitor-treated pear cell cultures converting *trans*-cinnamic acid to benzaldehyde. Physiol Plant 167: 64–74 30417393 10.1111/ppl.12871

[kiab111-B72] Sarkate A , Saini SS, Kumar P, Sharma AK, Sircar D (2018) Salicylaldehyde synthase activity from *Venturia inaequalis* elicitor-treated cell culture of apple. J Plant Physiol 221: 66–73 29247889 10.1016/j.jplph.2017.12.002

[kiab111-B73] Sas C , Müller F, Kappel C, Kent TV, Wright SI, Hilker M, Lenhard M (2016) Repeated inactivation of the first committed enzyme underlies the loss of benzaldehyde emission after the selfing transition in *Capsella*. Curr Biol 26: 3313–3319 27916528 10.1016/j.cub.2016.10.026

[kiab111-B74] Schiestl FP (2010) The evolution of floral scent and insect chemical communication. Ecol Lett 13: 643–656 20337694 10.1111/j.1461-0248.2010.01451.x

[kiab111-B75] Shi R , Sun YH, Li Q, Heber S, Sederoff R, Chiang VL (2010) Towards a systems approach for lignin biosynthesis in *Populus trichocarpa*: transcript abundance and specificity of the monolignol biosynthetic genes. Plant Cell Physiol 51: 144–163 19996151 10.1093/pcp/pcp175

[kiab111-B76] Shockey J , Browse J (2011) Genome-level and biochemical diversity of the acyl-activating enzyme superfamily in plants. Plant J 66: 143–160 21443629 10.1111/j.1365-313X.2011.04512.x

[kiab111-B77] Singh P , Preu L, Beuerle T, Kaufholdt D, Hänsch R, Beerhues L, Gaid M (2020) A promiscuous coenzyme A ligase provides benzoyl-coenzyme A for xanthone biosynthesis in *Hypericum*. Plant J 104: 1472–1490 33031578 10.1111/tpj.15012

[kiab111-B78] Tahvanainen J , Julkunen-Tiitto R, Kettunen J (1985) Phenolic glycosides govern the food selection pattern of willow feeding leaf beetles. Oecologia 67: 52–56 28309845 10.1007/BF00378451

[kiab111-B79] Tamura K , Stecher G, Peterson D, Filipski A, Kumar S (2013) MEGA6: Molecular Evolutionary Genetics Analysis version 6.0. Mol Biol Evol 30: 2725–2729 24132122 10.1093/molbev/mst197 PMC3840312

[kiab111-B80] Tuskan GA , DiFazio S, Jansson S, Bohlmann J, Grigoriev I, Hellsten U, Putnam N, Ralph S, Rombauts S, Salamov A, et al (2006) The genome of black cottonwood, *Populus trichocarpa* (Torr. & Gray). Science 313: 1596 16973872 10.1126/science.1128691

[kiab111-B81] Unsicker SB , Kunert G, Gershenzon J (2009) Protective perfumes: the role of vegetative volatiles in plant defense against herbivores. Curr Opin Plant Biol 12: 479–485 19467919 10.1016/j.pbi.2009.04.001

[kiab111-B82] Van Moerkercke A , Schauvinhold I, Pichersky E, Haring MA, Schuurink RC (2009) A plant thiolase involved in benzoic acid biosynthesis and volatile benzenoid production. Plant J 60: 292–302 19659733 10.1111/j.1365-313X.2009.03953.x

[kiab111-B83] Vogt T (2010) Phenylpropanoid biosynthesis. Mol Plant 3: 2–20 20035037 10.1093/mp/ssp106

[kiab111-B84] Wang HL , Chen J, Tian Q, Wang S, Xia X, Yin W (2014) Identification and validation of reference genes for *Populus euphratica* gene expression analysis during abiotic stresses by quantitative real-time PCR. Physiol Plant 152: 529–545 24720378 10.1111/ppl.12206

[kiab111-B85] Widhalm JR , Dudareva N (2015) A familiar ring to it: biosynthesis of plant benzoic acids. Mol Plant 8: 83–97 25578274 10.1016/j.molp.2014.12.001

[kiab111-B86] Xu M , Zhang B, Su X, Zhang S, Huang M (2011) Reference gene selection for quantitative real-time polymerase chain reaction in *Populus*. Anal Biochem 408: 337–339 20816740 10.1016/j.ab.2010.08.044

[kiab111-B87] Yang H , Barros-Rios J, Kourteva G, Rao X, Chen F, Shen H, Liu C, Podstolski A, Belanger F, Havkin-Frenkel D, et al (2017) A re-evaluation of the final step of vanillin biosynthesis in the orchid *Vanilla planifolia*. Phytochemistry 139: 33–46 28411481 10.1016/j.phytochem.2017.04.003

[kiab111-B88] Zenk MH (1967) Pathways of salicyl alcohol and salicin formation in *Salix purpurea* L. Phytochemistry 6: 245–252

