# Supplementary material for: A peroxisomal β-oxidative pathway contributes to the formation of C6–C1 aromatic volatiles in poplar
Source: Plant Physiol. 2021 Mar 16;186(2):891–909. doi: 10.1093/plphys/kiab111 (PMC8195509; doi:10.1093/plphys/kiab111)
Supplement: kiab111_Supplementary_Data [file kiab111_supplementary_data.pdf]

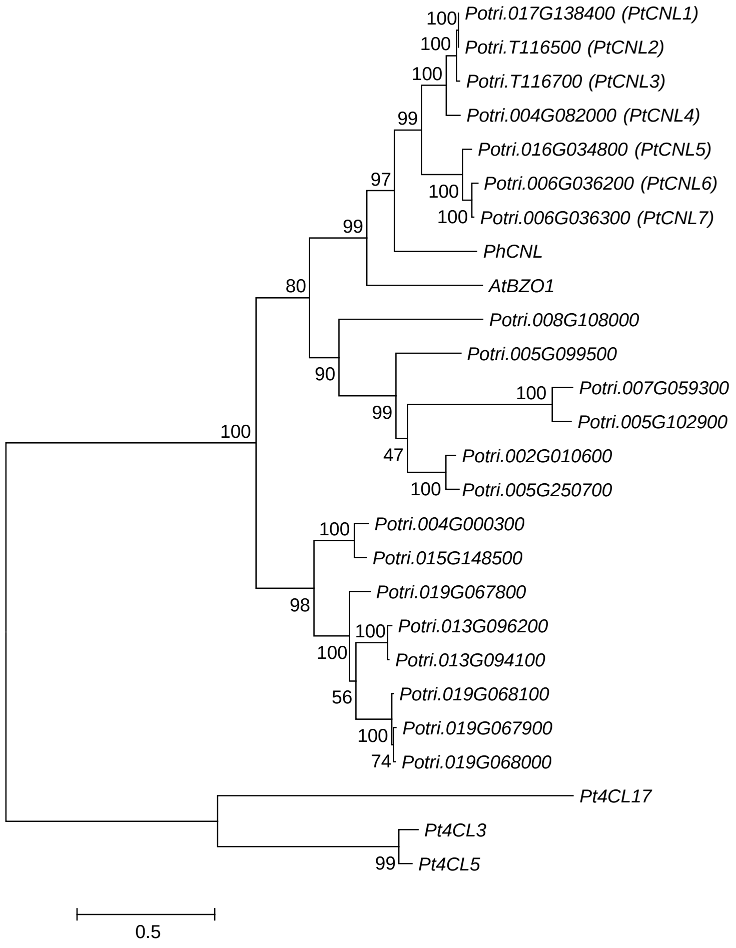

Supplemental Figure S1. Cladogram analysis of putative poplar *cinnamate-CoA ligase* (*CNL*) genes, characterized poplar *4-coumarate-CoA ligase* (*4CL*) genes, and characterized *cinnamate-CoA ligase* genes from other plants. The tree was inferred by using the Maximum Likelihood method based on the Tamura 3-parameter model implemented in MEGA6 (Tamura et al., 2013). Bootstrap values (n = 1000) are shown next to each node. The tree is drawn to scale, with branch lengths measured in the number of substitutions per site. Codon positions included were 1st+2nd+3rd+Noncoding. All positions with less than 80% site coverage were eliminated. *Pt4CL3*, EU603298 (Shi et al. 2010); *Pt4CL5*, EU603299 (Shi et al. 2010); *Pt4CL17*, EU603300 (Shi et al. 2010); *AtBZO1*, NM_105260 (Lee et al. 2012); *PhCNL*, JN120848 (Klempien et al. 2012).

```
                          10        20        30        40        50        60        70        80        90       100
                  ....|....|....|....|....|....|....|....|....|....|....|....|....|....|....|....|....|....|....|....|
PhCNL             MDELPKCGANYVPLTPLTFLTRAFKSYANRTSIIYAGARFTWEQTYKRCCRLASSLQSLNIVKNDVVSVLAPNVPATYEMHFAVPMAGAVLNTINTRLDP
AtBZO1            MDDLALCEANNVPLTPMTFLKRASECYPNRTSIIYGKTRFTWPQTYDRCCRLAASLISLNISKNDVVSVMAPNTPALYEMHFAVPMAGAVLNPINTRLDA
Potri.017G138400  MDQLLKCDANYVPLTPITFLKRANAVYANRTSVIYEGTRFTWSQTYERCCRLADSLRSLNVGKNDVVSVLAPNIPAVYEMHFAVPMAGAVLNTINIRLDA
Potri.T116700     MDQLLKCDANYVPLTPITFLKRANAVYANRTSVIYEGTRFTWSQTYERCCRLADSLRSLNVGKNDVVSVLAPNIPAVYEMHFAVPMAGAVLNTINTRLDA
Potri.004G082000  MDQLQKCDANYVPLTPVTFLKRASAVYANRTSVIYEGTRFTWRQTYERCCRLADSLRSLNVGKNDVVSVLAPNIPAVYEMHFAVPMAGAVLNTINTRLDA
Potri.016G034800  MDQLPKCDANYVPLTPINFLKRAAKVYGNRLSVVHERTHFTWQQTYERCLRLADSLRSFNIAKNDVVSVLAPNIPALYEMHFAVPMAGGVINAINTRLNP
Potri.006G036200  MDQLPKCDANYVPLTPINFLSRAAKVYGNRLSVVYEGIHFTWQQTYERCLRLADSLRYFNISKNDVVSVLAPNIPALYEMHFAVPMAGGVINAINTRLNP
Potri.006G036300  MDQLPKCDANYVPLTPINFLKRAAKVYGNRHSVVYEGIHFTWQQTYERCLCLADSLRSFDISKNDVVSVLAPNIPALYEMHFAVPMAGGVINAINTRLNP

                         110       120       130       140       150       160       170       180       190       200
                  ....|....|....|....|....|....|....|....|....|....|....|....|....|....|....|....|....|....|....|....|
PhCNL             MNIAIILKHSEAKLLFVDYEYLEKARKALELLMSTNFITAQNSKKISMPQVIL-IDDLYSPTRIQQQDQLEYEQLVHQGNPEYAPENI-----VDDEWDP
AtBZO1            TSIAAILRHAKPKILFLDRSFEALARESLHLLSSED-------SNLNLP-VIF-IHENDFPKR-ASFEELDYECLIQRGEP--TPSMVARMFRIQDEHDP
Potri.017G138400  KNIATILSHSGAKVFFVDYQYKELASKALSFLDGA------------VPSIIACIDDIDTPTG-VQFGQLEYEQLVQRGNPGYTGEL------VQDEWDP
Potri.T116700     RNIATILSHSGAKVFFVDYQYKELASKALSFLDGA------------VPSIIACIDDIDTPTG-VQFGQLEYEQLVQRGNPGYTDEL------VQDEWDP
Potri.004G082000  KNIATILIHSGAKVFFVDCQYKELADKALRFLEGP------------VPSIIASIDDVDTPTG-VRFGQFEYEQLVQRGNPGYTGEL------IQDEWDP
Potri.016G034800  NHVATILRHSEAKVFFVDYQFVQLARQALQIMMTSE----SISSKLVLPSVVL-IDDIESPTG-AIFGEWEYEQLVRKGNPGYIPYE------VQDEWDP
Potri.006G036200  NHVATILRHSEAKVFFVDYQFVQLARQALQILMASE----SNSSKLVLPSVVL-IDDIESPTG-AIFGEWEYEQLVRKGNPGYIPYE------VQDEWDP
Potri.006G036300  NHVATILRHSEAKVFFVDYQFVQLARQALQILMSSE----SISSKLVLPSVVL-IDDIESPTG-AIFGEWEYEQLVRKGNPGHIPFE------VQDEWDP

                         210       220       230       240       250       260       270       280       290       300
                  ....|....|....|....|....|....|....|....|....|....|....|....|....|....|....|....|....|....|....|....|
PhCNL             IVLNYTSGTTSEPKGVVYSHRGAFLSTLNTIMGWEMGTEPVYLWSLPMFHINGWTLTWGIAARGGTNVCIRNTTAQEIYSNITLHKVTHMCCAPTVFNIL
AtBZO1            ISLNYTSGTTADPKGVVISHRGAYLCTLSAIIGWEMGTCPVYLWTLPMFHCNGWTFTWGTAARGGTSVCMRHVTAPEIYKNIEMHNVTHMCCVPTVFNIL
Potri.017G138400  IALNYTSGTTSAPKGVVYSHRGAYLSSLSLILGWEMGNAPVYLWSLPMFHCNGWTFTWGVAARGGTNVCIRNTSAKDMYHNIAEHAVTHMCCAPIVFNIL
Potri.T116700     IALNYTSGTTSAPKGVVYSHRGAYLSSLSLILGWEMGNAPVYLWSLPMFHCNGWTFTWGVAARGGTNVCLRNTSAKDMYNNIAEHAVTHMCCAPIVFNIL
Potri.004G082000  IALNYTSGTTSAPKGVVYSHRGAYLSTLSLILGWEMGSAPVYLWSLPMFHCNGWTFTWGVAARGGTNVCIRNTSAKDMYQSIADHKVTHMCCAPIVFNIL
Potri.016G034800  IALNYTSGTTSEPKGVVYSHRGVFLGSLGIIIGWEMESEPVYLWSLPMFHCNGWTLAWGIAARGGTNVCLRNTTAKDMYRNIAQHKVTHMCCAPIVFNII
Potri.006G036200  IALNYTSGTTSEPKGVVYSHRSVFLGSLGEIIAWEMASEPVYMWSLPMFHANGWTLAWGIAARGGTNVCLRNTTAKDMYRNIAQHRVTHMCCAPIVFNIL
Potri.006G036300  IALNYTSGTTSEPKGVVYSHRGVFLGSLGVIIGWEMASEPVYMWSLPMFHCNGWTFAWGIAARGGTNVCLRNTTAKDMYRNIAQHRVTHMCCAPIVFNIL

                         310       320       330       340       350       360       370       380       390       400
                  ....|....|....|....|....|....|....|....|....|....|....|....|....|....|....|....|....|....|....|....|
PhCNL             LEAKPHERREITTPVQVMVGGAPPPTTLIGKIEELGFHVVHCYGITEAGGTTLVCEWQSEWNKLSREDQANLKARQGISVLALEDVDVKNSKTMQSVPHN
AtBZO1            LKGNSLDLSPRSGPVHVLTGGSPPPAALVKKVQRLGFQVMHAYGQTEATGPILFCEWQDEWNRLPENQQMELKARQGISILGLADVDVKNKETQKSAPRD
Potri.017G138400  LEARPHERREITSPVEILTGGAPPPASLLQDIERLGFHVTHAYGLTEATGPALVCEWQKKWNKLPQQDQAKLKARQGISILTLADADVKDLDTMVSVPRD
Potri.T116700     LEARPHERREITSPVEILTGGAPPPASLLQKIERLGFHVTHAYGLTEATGPALVCEWQKKWNKLPQQDQAKLKARQGISILTLADADVKDLDTMVSVPRD
Potri.004G082000  LEAKPHERREITSPVEILTGGAPPPASLLEKIESLGFHVTHAYGLTEATGPALVCEWQTKWNKLPQQDQSKLKARQGISILTLADADIKDLDTMASMPRD
Potri.016G034800  LEAKPDERREISSPVQILVGGAPPPASLLEKMKPLGFHVTHSYGLTEV-GPALVCEWQAKWNNLPSQDQSRIMARQGINTVALAHMDVKDLSTMISVPWD
Potri.006G036200  LEAKPEERREITSPVQILAGGAPPAASLLEKMKPLGFHVTHTYGLTEV-GPALVCEWQAKWNNLPSQDQSKIMARQGINTVALAHMDVKDLNTMIGVPWD
Potri.006G036300  LEAKPEERREIASPVQILVGGAPPPASLLEKMKPLGFHVTHSYGLTEV-GPALVCEWQAKWNNLPSQDQSKIMARQGINSVALAHMDVKDLNTMISVPRD

                         410       420       430       440       450       460       470       480       490       500
                  ....|....|....|....|....|....|....|....|....|....|....|....|....|....|....|....|....|....|....|....|
PhCNL             GKTMGEICLRGSSIMKGYFKNDKANSQVFKNGWFLTGDVAVIHQDGYLEIKDRCKDIIISGGENISSIEVENAILKHPSVIEAAVVAMPHPRWGETPCAF
AtBZO1            GKTMGEILIKGSSIMKGYLKNPKATFEAFKHGWLNTGDVGVIHPDGHVEIKDRSKDIIISGGENISSVEVENVLYKYPKVLETAVVAMPHPTWGETPCAF
Potri.017G138400  GKTMGEIVLRGSSIMKGYFKDPEATSKAFRNGWFATGDVGVIHPDGYLEIKDRSKDVIISGGENISSVELESVLYRHPRVLEAAVVAMPHPKWGESPCAF
Potri.T116700     GKTMGEIVLRGSSIMKGYFKDPEATSKAFRNGWLATGDVGVIHPDGYLEIKDRSKDVIISGGENISSVELESVLYRHPRVLEAAVVAMPHPRWGESPCAF
Potri.004G082000  GKTMGEIVLRGSSIMKGYFKDPEATSKAFRNGWFVTGDVGVIHPDGYLEIKDRSKDVIISGGENISSVELESVLYRHPRVLEAAVVAMPHPKWGESPCAF
Potri.016G034800  GKTMGEIVLRGSTVMKGYFKDPKATAKAFKNGWFATGDIGVIHPDGYLEIKDRSKDVIISGGENISSVELESVLYSHPRVLEAAVVAMPHPVWGESPCAF
Potri.006G036200  GKTMGEIVLKGSTVMKGYFKDPKATAKAFKNGWFATGDIGVIHPDGYLEIKDRSKDVIISGGENISSVELESVLYSHPRVLEAAVVAMPHPVWGESPCAF
Potri.006G036300  GKTMGEIVLKGSTVMKGYFKDPKATAKAFKNGWFATGDIGVIHPDGYLEIKDRSKDVIISGGENISSVELESVLYSHPRVLEAAVVAMPHPVWGESPCAF

                         510       520       530       540       550       560       570       580       590       600
                  ....|....|....|....|....|....|....|....|....|....|....|....|....|....|....|....|....|....|....|....|
PhCNL             VIKTKNPE-----------IKEADIIVHCKKELPGFMVPKHVQFLEELPKTGTGKVKKLQLREMAKSFGIFD--------NANQTSQILDL---P-----
AtBZO1            VVLEKSETTIKEDRVDKFQTRERNLIEYCRENLPHFMCPRKVVFLEELPKNGNGKILKPKLRDIAKGLVVED--------EINVIAKEVKR---PVGHFI
Potri.017G138400  ISVKKNSNGDTND------VKESDIIAYCKKNLPHFTVPKRVEFMAELPKTSTGKIQKFQLRALAQNFVVNEILPSKKITGHSQPSASGRVNTEVTEYAQ
Potri.T116700     ISVKKNSSGDTND------VKESDIIAYCKKNLPRFTVPKRVEFMAELPKTSTGKVLKIQLRALAQNFVV----------NA*-----------------
Potri.004G082000  ISVKKNSNGETND------VKESEIIAYCRKNLPRFMIPKRVEFMPELPKTSTGKIQKFQLREVAKNFAVNEKLPSKKPTGSSTPSVSGQVNTEVPGYAH
Potri.016G034800  LAIKKNSEGKSDD------VKEADIIAYCRKKLPHYMVPKKVEFMSELPKTSTGKVQKFQLRDLTRNFVVSESFPSKKTAQINAKTETQGHKAQPVRVPS
Potri.006G036200  LAIRKNSDGKSND------LKEADIIAYCKEKLPSYMIPKKVEFIPELPKNSTGKIQKFKLRDLARNFVVSETFPSKKSAQVNPNTELQGRQAQPARVAS
Potri.006G036300  LAIKKNSDGKSND------LKEADIIAYCRKKLPRYMVPKKVEFIPELPKTSTGKIQKFQLRDLARNFVVSETFPSKNTAQVNANTEMQGRQALPSRVAS

                         610
                  ....|....|..
PhCNL             --------ARI*
AtBZO1            --------SRI*
Potri.017G138400  GHEQVLALSRI*
Potri.T116700     ------------
Potri.004G082000  --EQVLAMSRI*
Potri.016G034800  --------SRI*
Potri.006G036200  --------SRI*
Potri.006G036300  --------SRI*
```

Supplemental Figure S2. Amino acid sequence comparison of putative *Populus trichocarpa* cinnamate-CoA ligases (CNL) Potri.017G138400 (PtCNL1), Potri.T116700 (PtCNL3), Potri.004G082000 (PtCNL4), Potri.016G034800 (PtCNL5), Potri.006G036200 (PtCNL6), and Potri.006G036300 (PtCNL7) with the characterized CNL from *Petunia hybrida* (Ph) and *Arabidopsis thaliana* (AtBZO1). The amino acid alignment was computed using the MUSCLE algorithm implemented in MEGA6 (Tamura et al., 2013) and visualized with the program BioEdit (http://www.mbio.ncsu.edu/bioedit/bioedit.html). Black boxes mark conserved residues and gray boxes mark similar amino acids. The peroxisomal targeting sequence I (PTS I) predicted by PSORT (https://www.psort.org/) is highlighted in red. The putative AMP-binding site as predicted by ProSite (https://prosite.expasy.org/scanprosite/) is highlighted in blue. AtBZO1, NP_176763 (Lee et al. 2012); PhCNL, AEO52693 (Klempien et al. 2012).

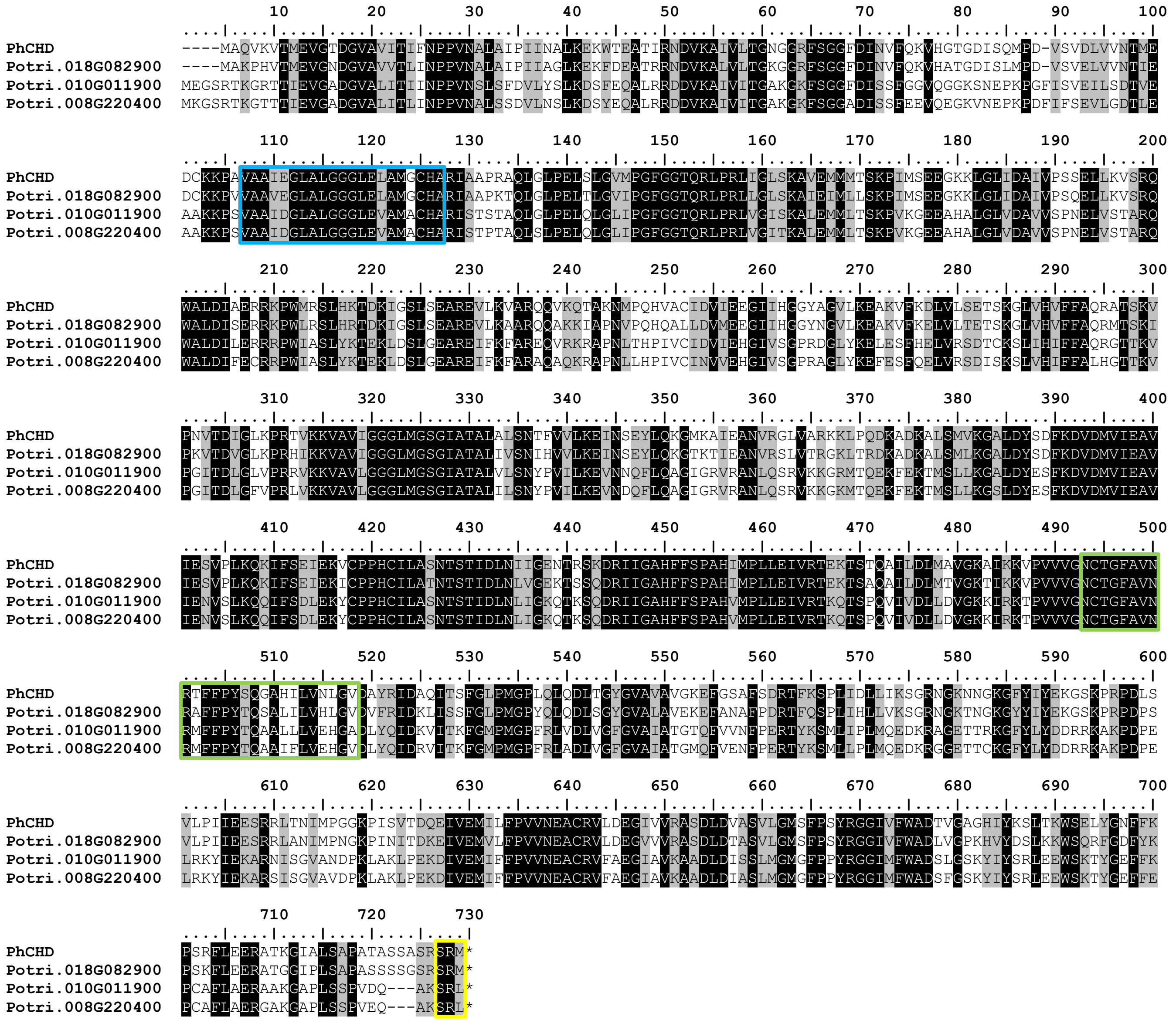

Supplemental Figure S3. Amino acid sequence comparison of putative *Populus trichocarpa* cinnamoyl-CoA hydratases/dehydrogenases (CHD) Potri.018G082900 (PtCHD1), Potri.010G011900 (PtCHD2), and Potri.008G220400 (PtCHD3) with the characterized CHD from *Petunia hybrida* (Ph). The amino acid alignment was computed using the MUSCLE algorithm implemented in MEGA6 (Tamura et al., 2013) and visualized with the program BioEdit (http://www.mbio.ncsu.edu/bioedit/bioedit.html). Black boxes mark conserved residues and gray boxes mark similar amino acids. The peroxisomal targeting sequence I (PTS I) predicted by PSORT (https://www.psort.org/) is highlighted in yellow. Putative conserved hydratase/isomerase and dehydrogenase regions predicted by ProSite (https://prosite.expasy.org/scanprosite/) are highlighted in blue and green, respectively. PhCHD, AFS41246 (Qualley et al. 2012).

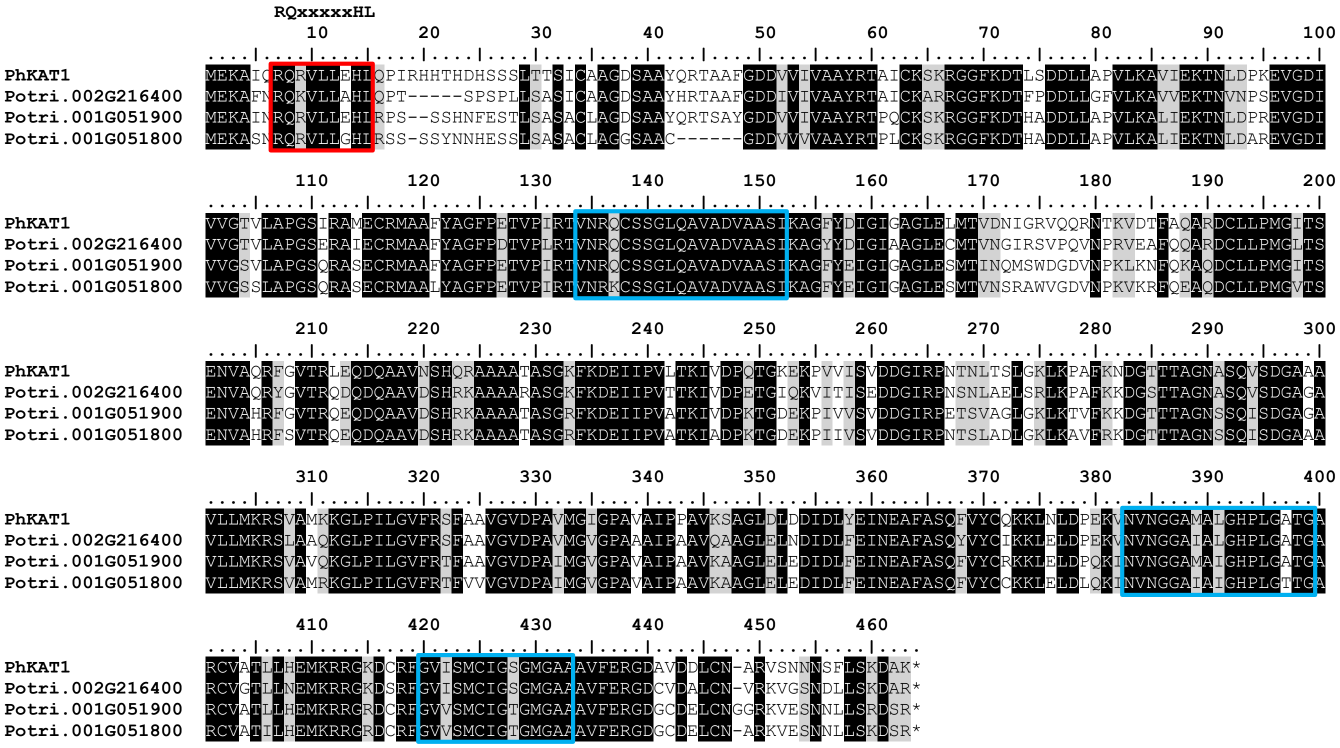

Supplemental Figure S4. Amino acid sequence comparison of putative *Populus trichocarpa* 3-ketoacyl-CoA thiolases (KAT) Potri.002G216400 (PtKAT1), Potri.001G051900 (PtKAT2), and Potri.001G051800 (PtKAT3) with the characterized KAT1 from *Petunia hybrida* (Ph). The amino acid alignment was computed using the MUSCLE algorithm implemented in MEGA6 (Tamura et al., 2013) and visualized with the program BioEdit (http://www.mbio.ncsu.edu/bioedit/bioedit.html). Black boxes mark conserved residues and gray boxes mark similar amino acids. The peroxisomal targeting sequence II (PTS II) as described by Reumann, 2004 is highlighted in red. Putative conserved regions involved in thiolase activity predicted by ProSite (https://prosite.expasy.org/scanprosite/) are highlighted in blue. PhKAT1, ACV70032 (Van Moerkercke et al. 2009).

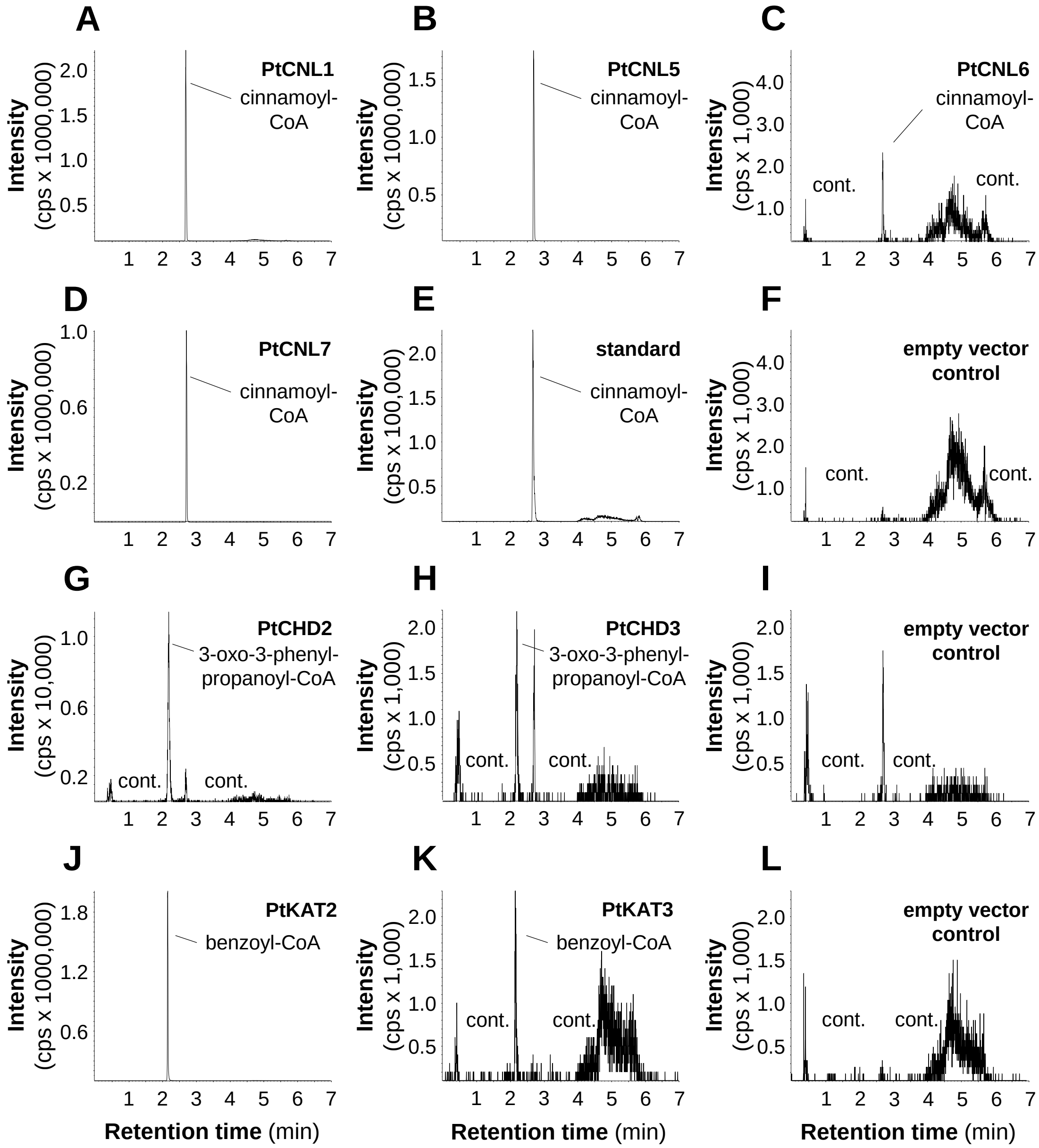

Supplemental Figure S5. Enzymatic activity of *Populus trichocarpa* cinnamate-CoA ligases (PtCNL), cinnamoyl-CoA hydratase/dehydrogenases (PtCHD), and 3-ketoacyl-CoA thiolases (PtKAT). Putative poplar *PtCNL*, *PtCHD*, and *PtKAT* genes were heterologously expressed in *Escherichia coli* as His_6-tag fusion proteins and recombinant proteins were purified using affinity chromatography. Purified proteins were incubated with the potential substrates *trans*-cinnamic acid (PtCNL), cinnamoyl-CoA (PtCHD), cinnamoyl-CoA (+ PtCHD1) (PtKAT) and the respective cosubstrates ATP, CoA (PtCNL); NAD^+ (PtCHD); CoA, NAD^+ (PtKAT). Reaction products were analyzed using liquid chromatography/tandem mass spectrometry (LC-MS/MS). cont., contamination; cps, counts per second.

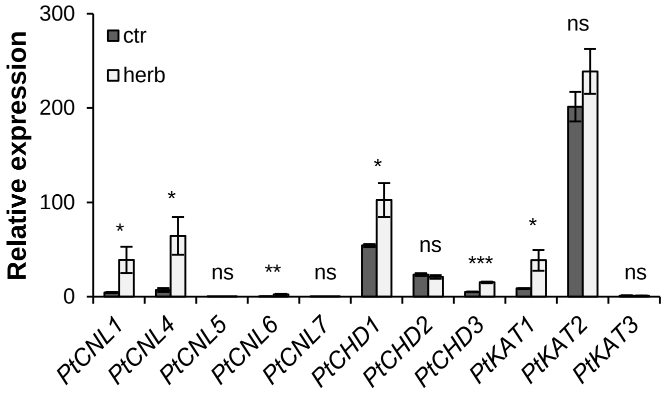

Supplemental Figure S6. Gene expression analysis of poplar *cinnamate-CoA ligases* (*CNL*), *cinnamoyl-CoA hydratases/dehydrogenases* (*CHD*), and *3-ketoacyl-CoA thiolases* (*KAT*). Transcript accumulation of *PtCNL1/4-7*, *PtCHD1-3*, and *PtKAT1-3* was measured in undamaged and *Chrysomela populi*-damaged *Populus trichocarpa* leaves using RT-qPCR with *tubulin* (Wang et al. 2014) as housekeeping gene. Means and SE are shown (n = 4). Asterisks indicate statistical significance as assessed by Student's *t*-test (ST) or Mann-Whitney Rank Sum Test (MW) (*P < 0.05; **P < 0.01; ***P < 0.001). *PtCNL1* (P = 0.029, T = 10.000, (MW)); *PtCNL4* (P = 0.029, t = -2.849, (ST)); *PtCNL5* (P = 0.067, t = -2.330, (ST)); *PtCNL6* (P = 0.002, t = -5.447, (ST)); *PtCNL7* (P = 0.141, t = -1.990, (ST)); *PtCHD1* (P = 0.029, T = 10.000, (MW)); *PtCHD2* (P = 0.272, t = 1.208, (ST)); *PtCHD3* (P < 0.001, t = -10.939, (ST)); *PtKAT1* (P = 0.029, T = 10.000, (MW)); *PtKAT2* (P = 0.235, t = -1.319, (ST)); *PtKAT3* (P = 0.367, t = 0.976, (ST)).

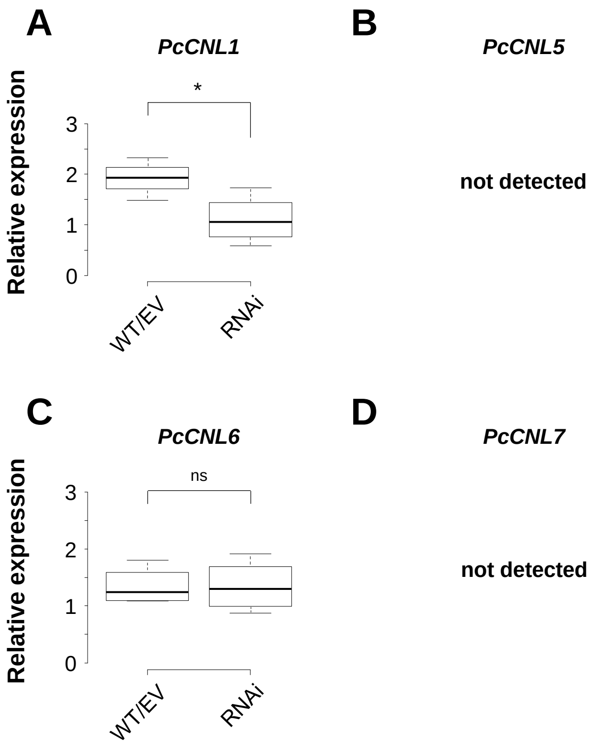

Supplemental Figure S7. RNAi-mediated knockdown of the *cinnamate-CoA ligases* (*CNL1* and *4*) in *Populus* x *canescens*. Transcript accumulation of *P.* x *canescens CNL1, CNL5, CNL6,* and *CNL7* in *Chrysomela populi*-damaged wild-type and transgenic *P.* x *canescens* trees are shown. Gene expression was measured using RT-qPCR with *ubiquitin* (Ramírez-Carvajal et al., 2007) as housekeeping gene. Medians ± quartiles and outliers are shown (n = 4 biological replicates). EV, empty vector; ns, not significant; WT, wild-type. Asterisks indicate statistical significance as assed by Student's *t*-test (ST) (**P* < 0.05). *PcCNL1* (*P* = 0.032, t = -2.779, (ST)); *PcCNL6* (*P* = 0.996, t = 0.00543, (ST)).

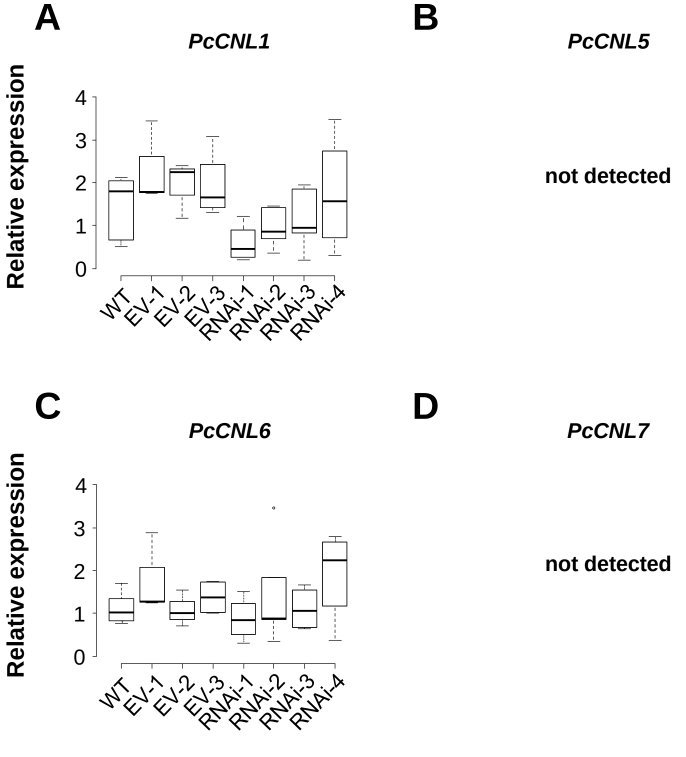

Supplemental Figure S8. RNAi-mediated knockdown of the *cinnamate-CoA ligases* (*CNL1* and *4)* in *Populus* x *canescens*. Transcript accumulation of *P.* x *canescens CNL1, CNL5, CNL6,* and *CNL7* in *Chrysomela populi*-damaged wild-type and transgenic *P.* x *canescens* trees are shown. Gene expression was measured using RT-qPCR with *ubiquitin* (Ramírez-Carvajal et al., 2007) as housekeeping gene. Medians ± quartiles and outliers are shown (n = 3-6 technical replicates). EV, empty vector; WT, wild type.

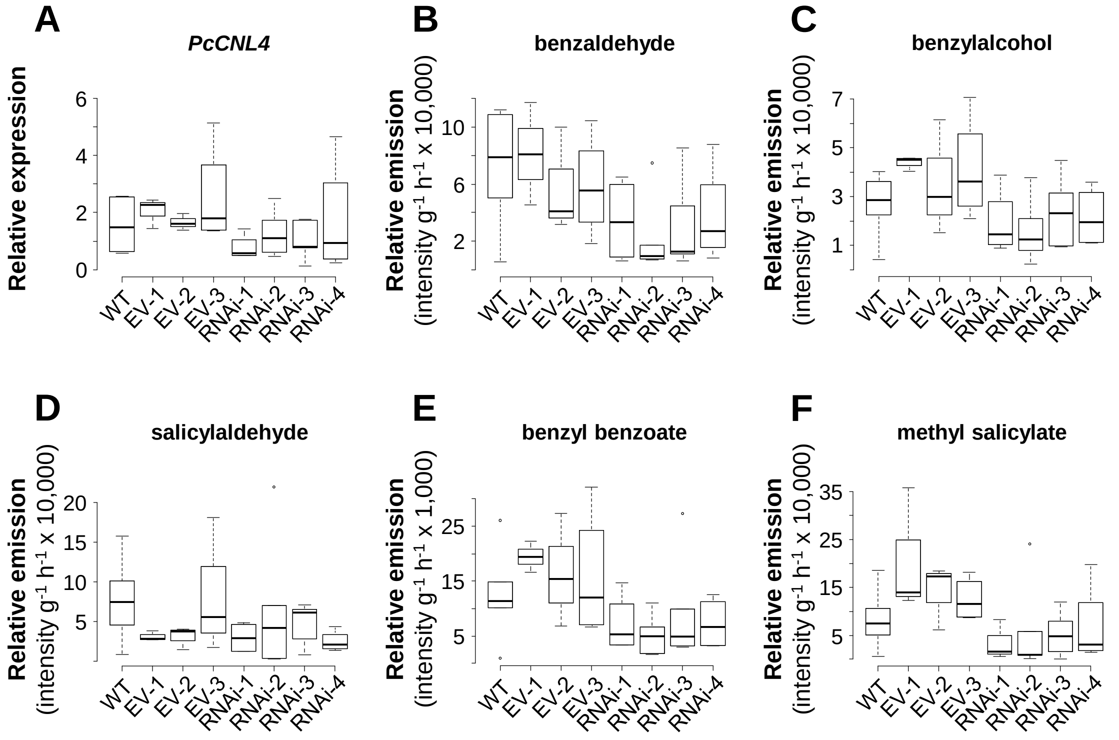

Supplemental Figure S9. Effect of RNAi-mediated knockdown of the *cinnamate-CoA ligases* (*CNL1* and *4)* in *Populus* x *canescens* on the emission of aromatic compounds. Transcript accumulation of *P.* x *canescens CNL4* (A), and the relative emission of benzaldehyde (B), benzylalcohol (C), salicylaldehyde (D), benzyl benzoate (E), and methyl salicylate (F) of *Chrysomela populi*-damaged wild-type and transgenic *P.* x *canescens* trees are shown. Gene expression was measured using RT-qPCR, with *ubiquitin* (Ramírez-Carvajal et al., 2007) as housekeeping gene. Volatiles were analyzed using GC-MS. Medians ± quartiles, and outliers are shown (n = 3-6 technical replicates). EV, empty vector; WT, wild type.

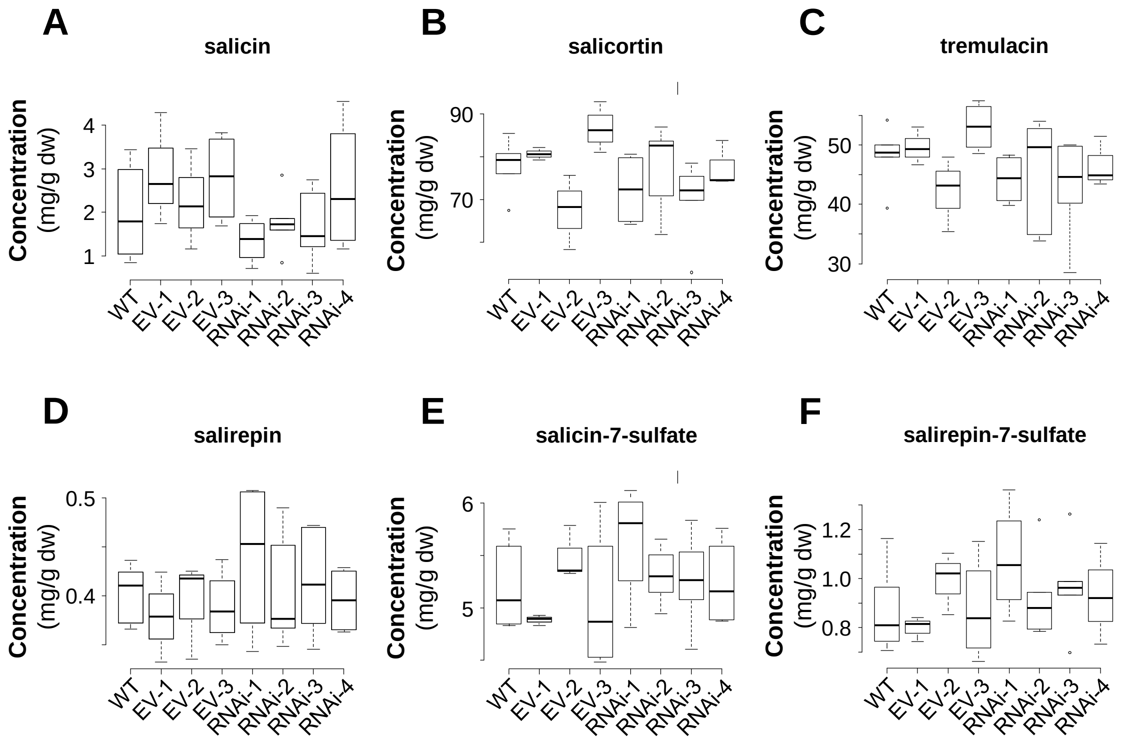

Supplemental Figure S10. Effect of RNAi-mediated knockdown of the *cinnamate-CoA ligases* (*CNL1* and *4)* in *Populus* x *canescens* on the formation of salicinoids. Concentration of salicin (A), salicortin (B), tremulacin (C), salirepin (D), salicin-7-sulfate (E), and salirepin-7-sulfate (F) in leaves of *Chrysomela populi*-damaged wild-type and transgenic *P.* x *canescens* trees are shown. Compounds were extracted with methanol from freeze-dried leaf material and analyzed using LC-MS/MS and HPLC-UV. Medians ± quartiles, and outliers are shown (n = 3-6 technical replicates). EV, empty vector; dw, dry weight; WT, wild type.

Supplemental Table S1. Volatiles (ng g^{-1} h^{-1} fw) emitted from undamaged (control) and *Chrysomela populi*-damaged (herbivory) *Populus trichocarpa* leaves. Compounds were collected for 6 h using a push-pull system and Poropak filter. Eluates were analyzed using GC-MS and quantified by GC-FID. Means and SE (n = 8) are given. Differences between treatments were analyzed by Student's *t*-test (ST) or Mann-Whitney Rank Sum Test (MW). fw, fresh weight, (*E*)-DMNT, (*E*)-4,8-dimethylnona-1,3,7-triene; (*E,E*)-TMTT, (*E*,*E*)-4,8,12-trimethyltrideca-1,3,7,11-tetraene. Compounds marked with # were identified by comparison of retention time and mass spectrum to those of authentic standards. Other compounds were identified by database comparisons.

compound	control	herbivory	*P*-value	t-value/*T-value*
Aromatics				
benzaldehyde#	0.28 ± 0.10	20.22 ± 9.07	<0.001	*36.000* (MW)
benzylalcohol#	2.13 ± 0.40	21.13 ± 3.59	<0.001	*36.000* (MW)
2-phenylethanol#/(*E*)-DMNT	0.00 ± 0.00	85.01 ± 10.63	<0.001	*36.000* (MW)
salicylaldehyde#	3.11 ± 1.17	20.23 ± 7.91	0.007	*43.000* (MW)
methyl salicylate#	0.62 ± 0.16	1.37 ± 0.27	0.031	-2.398 (ST)
salicylalcohol	0.00 ± 0.00	17.55 ± 4.03	<0.001	*36.000* (MW)
eugenol	0.46 ± 0.13	1.35 ± 0.24	0.006	-3.258 (ST)
isoamyl benzoate	0.00 ± 0.00	0.44 ± 0.06	<0.001	*36.000* (MW)
cis-3-hexenyl benzoate#	0.47 ± 0.11	5.37 ± 1.36	<0.001	*36.000* (MW)
hexenyl benzoate/(*E*,*E*)-TMTT	0.39 ± 0.19	14.58 ± 3.18	<0.001	*36.000* (MW)
benzyl benzoate#	0.91 ± 0.25	2.91 ± 0.92	0.036	-2.315 (ST)
Nitrogenous compounds				
(*E*)-3-methylbutyraldoxime	0.00 ± 0.00	39.19 ± 7.87	<0.001	*36.000* (MW)
(*Z*)-3-methylbutyraldoxime	0.00 ± 0.00	23.75 ± 3.55	<0.001	*36.000* (MW)
(*E*)-2-methylbutyraldoxime	0.00 ± 0.00	45.44 ± 7.10	<0.001	*36.000* (MW)
(*Z*)-2-methylbutyraldoxime	0.00 ± 0.00	12.03 ± 1.79	<0.001	*36.000* (MW)
benzyl cyanide	0.49 ± 0.16	50.71 ± 7.62	<0.001	*36.000* (MW)
(*E*)-phenylacetaldoxime	0.00 ± 0.00	5.16 ± 0.80	<0.001	*36.000* (MW)
(*Z*)-phenylacetaldoxime	0.00 ± 0.00	4.77 ± 0.83	<0.001	*36.000* (MW)
indole	0.15 ± 0.08	27.33 ± 4.27	<0.001	*36.000* (MW)
2-phenylnitroethane	0.00 ± 0.00	11.00 ± 1.84	<0.001	*36.000* (MW)
Green leaf volatiles				
1-hexanol	0.00 ± 0.00	22.55 ± 4.27	<0.001	*36.000* (MW)

Supplemental Table S1. continued

compound	control	herbivory	*P*-value	t-value/*T-value*
Monoterpenoids				
α-pinene[#]	8.97 ± 2.31	10.91 ± 2.18	0.442	*60.000* (MW)
sabinene	5.84 ± 1.43	9.77 ± 2.09	0.130	*53.000* (MW)
β-pinene[#]	2.79 ± 0.72	4.24 ± 0.91	0.105	*52.000* (MW)
p-cymene	1.72 ± 0.38	3.48 ± 0.49	0.012	-2.876 (ST)
limonene[#]	3.51 ± 1.08	5.54 ± 1.14	0.217	-1.294 (ST)
1,8-cineole	24.08 ± 6.06	58.75 ± 11.38	0.018	-2.690 (ST)
(*Z*)-β-ocimene	0.00 ± 0.00	19.79 ± 4.29	<0.001	*36.000* (MW)
(*E*)-β-ocimene[#]	6.66 ± 2.44	355.94 ± 86.77	<0.001	*36.000* (MW)
α-terpineol[#]	3.13 ± 0.81	9.01 ± 2.12	0.021	-2.589 (ST)
Sesquiterpenoids				
α-copaene	3.60 ± 1.10	5.98 ± 1.12	0.083	*51.000* (MW)
calarene	1.24 ± 0.35	2.16 ± 0.43	0.116	-1.676 (ST)
β-cubebene	1.23 ± 0.40	2.12 ± 0.40	0.141	-1.561 (ST)
α-amorphene	4.19 ± 1.18	7.62 ± 1.42	0.065	50.000 (MW)
(*Z,E*)-α-farnesene	2.14 ± 0.41	16.91 ± 2.64	<0.001	*36.000* (MW)
γ-cadinene	3.41 ± 0.96	6.02 ± 1.17	0.065	*50.000* (MW)
δ-cadinene	8.07 ± 2.34	14.90 ± 3.03	0.065	*50.000* (MW)
cadinol	1.86 ± 0.37	4.30 ± 0.81	0.016	-2.751 (ST)
(*E,E*)-α-farnesene	64.79 ± 15.02	1192.88 ± 192.07	<0.001	*36.000* (MW)

Supplemental Table S2. Content of non-polar benzenoid compounds (µg/g fresh weight) in undamaged (control) and *Chrysomela populi*-damaged (herbivory) *Populus trichocarpa* leaves. Compounds were extracted with hexane from fresh plant material and analyzed using GC-MS/GC-FID. Means and SE (n = 8) are given. Differences between treatments were analyzed by Student's *t*-test (ST) or Mann-Whitney Rank Sum Test (MW).

compound	control	herbivory	***P*-value**	**t-value/*T-value***
benzaldehyde	0.14 ± 0.01	0.18 ± 0.01	0.028	*47.000* (MW)
benzylalcohol	0.51 ± 0.10	0.80 ± 0.12	0.038	*48.000* (MW)
salicylaldehyde	18.49 ± 2.33	27.82 ± 3.07	0.030	-2.419 (ST)
benzyl benzoate	0.50 ± 0.08	1.53 ± 0.25	<0.001	*36.000* (MW)

Supplemental Table S3. Aromatic amino acid and aromatic carboxylic acid content (µg/g dry weight) in undamaged (control) and *Chrysomela populi*-damaged (herbivory) *Populus trichocarpa* leaves. Compounds were extracted with methanol from freeze-dried plant material and analyzed using LC-MS/MS. NA, not available; nd, not detected. Means and SE (n = 8) are given. Differences between treatments were analyzed by Student's *t*-test (ST) or Mann-Whitney Rank Sum Test (MW).

compound	control	herbivory	*P*-value	t-value/*T-value*
phenylalanine	19.80 ± 1.21	32.69 ± 2.29	<0.001	-4.969 (ST)
tyrosine	7.37 ± 0.53	8.60 ± 0.69	0.176	-1.425 (ST)
tryptophan	10.76 ± 1.32	71.67 ± 7.75	<0.001	*36.000* (MW)
trans-cinnamic acid	37.59 ± 5.19	38.79 ± 4.03	0.857	-0.813 (ST)
2-hydroxycinnamic acid	nd	nd	NA	NA
4-hydroxycinnamic acid	2.96 ± 0.24	5.00 ± 0.23	<0.001	-6.161 (ST)
caffeic acid	1.73 ± 0.10	3.40 ± 0.38	<0.001	*36.000* (MW)
ferulic acid	0.45 ± 0.02	0.71 ± 0.04	<0.001	-5.285 (ST)
gallic acid	1.29 ± 0.07	1.42 ± 0.07	0.205	-1.330 (ST)
benzoic acid	nd	nd	NA	NA
3-hydroxybenzoic acid	3.80 ± 0.29	4.02 ± 0.30	0.600	-0.536 (ST)
4-hydroxybenzoic acid	1.38 ± 0.08	3.30 ± 0.41	<0.001	*36.000* (MW)
2,3-dihydroxybenzoic acid	0.00 ± 0.00	0.56 ± 0.24	0.010	*44.000* (MW)
2,5-dihydroxybenzoic acid	0.00 ± 0.00	0.50 ± 0.18	<0.001	*36.000* (MW)

Supplemental Table S4. Phytohormone content (ng/g dry weight) in undamaged (control) and *Chrysomela populi*-damaged (herbivory) *Populus trichocarpa* leaves. Compounds were extracted with methanol from freeze-dried plant material and analyzed using LC-MS/MS. Means and SE (n = 8) are given. Differences between treatments were analyzed by Student's *t*-test (ST) or Mann-Whitney Rank Sum Test (MW).

**compound**	**control**	**herbivory**	***P*-value**	**t-value/*T-value***
salicylic acid	624.33 ± 118.65	969.23 ± 176.28	0.105	-1.735 (ST)
jasmonic acid	125.56 ± 22.90	855.31 ± 122.91	<0.001	*36.000* (MW)
abscisic acid	94.88 ± 6.40	130.78 ± 8.81	0.003	-3.524 (ST)
(-)-jasmonoyl-L-isoleucine	1.23 ± 0.22	15.23 ± 1.87	<0.001	*36.000* (MW)
(+)-7-*iso*-jasmonoyl-L-isoleucine	3.38 ± 0.57	72.25 ± 8.19	<0.001	*36.000* (MW)
12-oxophytodienoic acid	194.40 ± 30.57	503.30 ± 69.88	<0.001	-4.330 (ST)
12-hydroxyjasmonic acid	252.04 ± 64.33	6908.47 ± 1396.15	<0.001	*36.000* (MW)
12-hydroxyjasmonic acid-isoleucine	8.61 ± 1.50	213.18 ± 32.68	<0.001	*36.000* (MW)
12-carboxyjasmonic acid-isoleucine	0.00 ± 0.00	16.04 ± 2.27	<0.001	*36.000* (MW)

Supplemental Table S5. Incorporation of D_7-cinnamic acid (D_7-CA) into aromatic carboxylic acids in *Chrysomela populi*-damaged *Populus trichocarpa* leaves. Compounds were extracted with methanol from freeze-dried plant material and analyzed using LC-MS/MS. The amount of each compound, labeled and unlabeled, is given as the percentage of the total amount of that compound. nd, not detected. Means and SE (n = 3 - 4) are given.

compound	H_2O control (%)	D_7CA feeding (%)
cinnamic acid	100 ± 0.0	3.9 ± 1.6
D_7-cinnamic acid	nd	96.1 ± 1.6
2-hydroxycinnamic acid	nd	nd
D_6-2-hydroxycinnamic acid	nd	nd
4-hydroxycinnamic acid	100 ± 0.0	39.3 ± 19.4
D_6-4-hydroxycinnamic acid	nd	60.7 ± 19.4
caffeic acid	100 ± 0.0	74.2 ± 10.9
D_5-caffeic acid	nd	25.8 ± 10.9
ferulic acid	100 ± 0.0	78.7 ± 4.2
D_5-ferulic acid	nd	21.3 ± 4.2
benzoic acid (BA)	nd	nd
D_5-benzoic acid	nd	nd
salicylic acid	100 ± 0.0	95.3 ± 1.3
D_4-salicylic acid	nd	4.7 ± 1.3
3-hydroxy BA	nd	nd
D_4-3-hydroxy BA	nd	nd
4-hydroxy BA	nd	nd
D_4-4-hydroxy BA	nd	nd
2,3 dihydroxy BA	nd	nd
D_3-2,3 dihydroxy BA	nd	nd
2,5 dihydroxy BA	100 ± 0.0	94.5 ± 1.1
D_3-2,5 dihydroxy BA	nd	5.5 ± 1.1
gallic acid	100 ± 0.0	100 ± 0.0
D_2-gallic acid	nd	nd

Supplemental Table S6. Predicted subcellular localization of poplar cinnamate-CoA ligases (PtCNL). [1] https://rostlab.org/services/loctree3/; [2] http://www.cbs.dtu.dk/services/DeepLoc-1.0/, (Almagro Armenteros et al. 2017); [3] https://mendel.imp.ac.at/pts1/, (Neuberger et al. 2003a, Neuberger et al. 2003b); [4] http://ppp.gobics.de/, (Reumann et al. 2012); [5] http://www.cbs.dtu.dk/services/TargetP-2.0/, (Almagro Armenteros et al. 2019); [6] http://www.csbio.sjtu.edu.cn/bioinf/plant-multi/, (Chou, 2004; Chou and Shen, 2007, 2008, 2010; Shen and Chou, 2006).

**Prediction software**	**Predicted location**					
	**PtCNL1**	**PtCNL3**	**PtCNL4**	**PtCNL5**	**PtCNL6**	**PtCNL7**
**LocTree3[1]**	Peroxisome (Expected accuracy: 88%)	Peroxisome (Expected accuracy: 88%)	Peroxisome (Expected accuracy: 88%)	Peroxisome (Expected accuracy: 88%)	Peroxisome (Expected accuracy: 87%)	Peroxisome (Expected accuracy: 87%)
**DeepLoc-1.0[2]**	Peroxisome (Likelihood 0.60)	Peroxisome (Likelihood 0.52)	Peroxisome (Likelihood 0.57)	Peroxisome (Likelihood 0.57)	Peroxisome (Likelihood 0.56)	Peroxisome (Likelihood 0.58)
**PTS1 Predictor[3]**	PTS1 targeting (score 8.59)	No PTS1 targeting (score -40.00)	PTS1 targeting (score 6.49)	PTS1 targeting (score 13.27)	PTS1 targeting (score 13.79)	PTS1 targeting (score 13.88)
**PredPlantPTS1[4]**	PTS1 domain	No PTS1 domain	PTS1 domain	PTS1 domain	PTS1 domain	PTS1 domain
**TargetP-2.0[5]**	No mitochondrial transfer peptide/No chloroplast transfer peptide/No signal peptide					
**Plant-mPLoc[6]**	Peroxisome	Peroxisome	Peroxisome	Peroxisome	Peroxisome	Peroxisome

references:

Almagro Armenteros JJ, Salvatore M, Emanuelsson O, Winther O, von Heijne G, Elofsson A, Nielsen H (2019) Detecting sequence signals in targeting peptides using deep learning. Life Sci Alliance **2:** e201900429

Almagro Armenteros JJ, Sønderby CK, Sønderby SK, Nielsen H, Winther O (2017) DeepLoc: prediction of protein subcellular localization using deep learning. Bioinformatics **33:** 3387-3395

Chou K-C (2004) Using amphiphilic pseudo amino acid composition to predict enzyme subfamily classes. Bioinformatics **21:** 10-19

Chou K-C, Shen H-B (2007) Large-scale plant protein subcellular location prediction. J Cell Biochem **100:** 665-678

Chou K-C, Shen H-B (2008) Cell-PLoc: a package of Web servers for predicting subcellular localization of proteins in various organisms. Nat Protoc **3:** 153-162

Chou K-C, Shen H-B (2010) Plant-mPLoc: A top-down strategy to augment the power for predicting plant protein subcellular localization. PLOS ONE **5:** e11335

Neuberger G, Maurer-Stroh S, Eisenhaber B, Hartig A, Eisenhaber F (2003a) Motif refinement of the Peroxisomal Targeting Signal 1 and evaluation of taxon-specific differences. J Mol Biol **328:** 567-579

Neuberger G, Maurer-Stroh S, Eisenhaber B, Hartig A, Eisenhaber F (2003b) Prediction of Peroxisomal Targeting Signal 1 containing proteins from amino acid sequence. J Mol Biol **328:** 581-592

Reumann S, Buchwald D, Lingner T (2012) PredPlantPTS1: A web server for the prediction of plant peroxisomal proteins. Front Plant Sci **3:** 194

Shen H-B, Chou K-C (2006) Ensemble classifier for protein fold pattern recognition. Bioinformatics **22:** 1717-1722

Supplemental Table S7. Predicted subcellular localization of poplar cinnamoyl-CoA hydratases/dehydrogenases (PtCHD) and 3-ketoacyl-CoA thiolases (PtKAT). [1] https://rostlab.org/services/loctree3/; [2] http://www.cbs.dtu.dk/services/DeepLoc-1.0/, (Almagro Armenteros et al. 2017); [3] https://mendel.imp.ac.at/pts1/, (Neuberger et al. 2003a, Neuberger et al. 2003b); [4] http://ppp.gobics.de/, (Reumann et al. 2012); [5] http://www.cbs.dtu.dk/services/TargetP-2.0/, (Almagro Armenteros et al. 2019); [6] http://www.csbio.sjtu.edu.cn/bioinf/plant-multi/, (Chou, 2004; Chou and Shen, 2007, 2008, 2010; Shen and Chou, 2006).

**Prediction software**	**Predicted location**					
	**PtCHD1**	**PtCHD2**	**PtCHD3**	**PtKAT1**	**PtKAT2**	**PtKAT3**
**LocTree3[1]**	Peroxisome (Expected accuracy: 89%)	Peroxisome (Expected accuracy: 96%)	Peroxisome (Expected accuracy: 95%)	Peroxisome (Expected accuracy: 95%)	Peroxisome (Expected accuracy: 96%)	Peroxisome (Expected accuracy: 96%)
**DeepLoc-1.0[2]**	Peroxisome (Likelihood 0.98)	Peroxisome (Likelihood 0.98)	Peroxisome (Likelihood 0.98)	Peroxisome (Likelihood 0.61)	Peroxisome (Likelihood 0.59)	Peroxisome (Likelihood 0.60)
**PTS1 Predictor[3]**	PTS1 targeting (score 9.04)	PTS1 targeting (score 13.27)	PTS1 targeting (score 12.13)	No PTS1 targeting (score -42.79)	No PTS1 targeting (score -35.04)	No PTS1 targeting (score -34.84)
**PredPlantPTS1[4]**	PTS1 domain	PTS1 domain	PTS1 domain	No PTS1 domain	No PTS1 domain	No PTS1 domain
**TargetP-2.0[5]**	No mitochondrial transfer peptide/No chloroplast transfer peptide/No signal peptide					
**Plant-mPLoc[6]**	Peroxisome	Peroxisome	Peroxisome	Peroxisome	Peroxisome	Peroxisome

references:

Almagro Armenteros JJ, Salvatore M, Emanuelsson O, Winther O, von Heijne G, Elofsson A, Nielsen H (2019) Detecting sequence signals in targeting peptides using deep learning. Life Sci Alliance **2:** e201900429

Almagro Armenteros JJ, Sønderby CK, Sønderby SK, Nielsen H, Winther O (2017) DeepLoc: prediction of protein subcellular localization using deep learning. Bioinformatics **33:** 3387-3395

Chou K-C (2004) Using amphiphilic pseudo amino acid composition to predict enzyme subfamily classes. Bioinformatics **21:** 10-19

Chou K-C, Shen H-B (2007) Large-scale plant protein subcellular location prediction. J Cell Biochem **100:** 665-678

Chou K-C, Shen H-B (2008) Cell-PLoc: a package of Web servers for predicting subcellular localization of proteins in various organisms. Nat Protoc **3:** 153-162

Chou K-C, Shen H-B (2010) Plant-mPLoc: A top-down strategy to augment the power for predicting plant protein subcellular localization. PLOS ONE **5:** e11335

Neuberger G, Maurer-Stroh S, Eisenhaber B, Hartig A, Eisenhaber F (2003a) Motif refinement of the Peroxisomal Targeting Signal 1 and evaluation of taxon-specific differences. J Mol Biol **328:** 567-579

Neuberger G, Maurer-Stroh S, Eisenhaber B, Hartig A, Eisenhaber F (2003b) Prediction of Peroxisomal Targeting Signal 1 containing proteins from amino acid sequence. J Mol Biol **328:** 581-592

Reumann S, Buchwald D, Lingner T (2012) PredPlantPTS1: A web server for the prediction of plant peroxisomal proteins. Front Plant Sci **3:** 194

Shen H-B, Chou K-C (2006) Ensemble classifier for protein fold pattern recognition. Bioinformatics **22:** 1717-1722

Supplemental Table S8. Enzymatic activity of *Populus trichocarpa* cinnamate-CoA ligases (PtCNL) assayed with different substrates. Putative poplar PtCNL were heterologously expressed in *Escherichia coli* as His_6-tag fusion proteins and recombinant proteins were purified using affinity chromatography. Purified proteins were incubated with the potential substrates listed plus CoA and ATP. Reaction products were analyzed using liquid chromatography/tandem mass spectrometry (LC-MS/MS). Enzymatic activity is displayed as percentage of used substrate. Means and SE are given (n = 3).

substrate	PtCNL1	PtCNL4	PtCNL5	PtCNL6	PtCNL7
trans-cinnamic acid	98.5 ± 0.6	98.5 ± 0.7	51.0 ± 3.0	0.2 ± 0.2	41.1 ± 3.0
2-hydroxycinnamic acid	99.6 ± 0.3	98.1 ± 0.9	42.2 ± 1.0	1.6 ± 1.1	46.8 ± 3.5
4-hydroxycinnamic acid	42.7 ± 1.2	38.9 ± 1.9	56.2 ± 0.4	1.4 ± 1.4	75.0 ± 3.7
caffeic acid	11.0 ± 0.7	8.0 ± 0.7	16.9 ± 1.0	0.2 ± 0.1	4.7 ± 0.5
ferulic acid	8.1 ± 0.6	7.0 ± 1.0	21.4 ± 0.5	2.0 ± 0.7	10.1 ± 0.6
benzoic acid	1.8 ± 0.4	3.1 ± 1.2	21.0 ± 1.2	5.0 ± 1.8	7.5 ± 0.5

Supplemental Table S9. Enzymatic activity of *Populus trichocarpa* cinnamoyl-CoA hydratases/dehydrogenases (PtCHD) assayed with different substrates. Putative poplar PtCHD were heterologously expressed in *Escherichia coli* as His_6-tag fusion proteins and recombinant proteins were purified using affinity chromatography. Purified proteins were incubated with its potential substrates listed and NAD^+. Reaction products were analyzed using liquid chromatography/tandem mass spectrometry (LC-MS/MS). Enzymatic activity is displayed as percentage of used substrate. Means and SE are given (n = 3).

substrate	PtCHD1	PtCHD2	PtCHD3
cinnamoyl-CoA	78.2 ± 1.7	8.5 ± 0.9	2.0 ± 1.0
2-hydroxycinnamoyl-CoA	98.1 ± 0.1	6.9 ± 0.4	33.6 ± 0.6
4-hydroxycinnamoyl-CoA	97.2 ± 0.1	2.5 ± 0.7	1.8 ± 1.2

Supplemental Table S10. Enzymatic activity of *Populus trichocarpa* 3-ketoacyl-CoA thiolases (PtKAT) assayed with different substrates. Putative poplar PtKAT were heterologously expressed in *Escherichia coli* as His_6-tag fusion proteins and recombinant proteins were purified using affinity chromatography. Purified proteins were incubated in combined assays with PtCHD1, its potential substrates listed, and the cosubstrates CoA and NAD^+. Reaction products were analyzed using liquid chromatography/tandem mass spectrometry (LC-MS/MS). Enzymatic activity is displayed as intensity of the produced acetyl-CoA. Means and SE are given (n = 3). nd, not detected.

substrate	PtKAT1	PtKAT2	PtKAT3
cinnamoyl-CoA + PtCHD1	3.51E+06 ± 5.56E+04	3.61E+06 ± 7.12E+04	8.52E+03 ± 2.66e+02
2-hydroxycinnamoyl-CoA + PtCHD1	nd	nd	nd
4-hydroxycinnamoyl-CoA + PtCHD1	2.65E+06 ± 5.29E+04	2.59E+06 ± 5.97E+04	nd

Supplemental Table S11. Enzymatic activity of *Populus trichocarpa* 3-ketoacyl-CoA thiolases (PtKAT) assayed with different substrates. Putative poplar PtKAT were heterologously expressed in *Escherichia coli* as His_6-tag fusion proteins and recombinant proteins were purified using affinity chromatography. Purified proteins were incubated in combined assays with PtCHD1, its potential substrates listed, and the cosubstrates CoA and NAD^+. Reaction products were analyzed using liquid chromatography/tandem mass spectrometry (LC-MS/MS). Enzymatic activity is displayed as intensity of the produced aromatic CoA ester product. Means and SE are given (n = 3). nd, not detected.

substrate	PtKAT1	PtKAT2	PtKAT3
cinnamoyl-CoA + PtCHD1	2.50E+06 ± 2.91E+04	2.56E+06 ± 6.39E+04	4.45E+03 ± 3.17E+02
2-hydroxycinnamoyl-CoA + PtCHD1	nd	nd	nd
4-hydroxycinnamoyl-CoA + PtCHD1	1.34E+05 ± 9.56E+02	1.32E+05 ± 2.27E+03	nd

Supplemental Table S12. Volatiles (ng g^{-1} h^{-1} fresh weight) emitted from *Chrysomela populi*-damaged *Populus* x *canescens* leaves of *cinnamate-CoA ligase (CNL1* and *4)* knockdown (RNAi), empty vector (EV), and wild-type (WT) trees. Compounds were collected for 6 h using a push-pull system and Poropak filter. Eluates were analyzed using GC-MS and quantified by GC-FID. Means and SE (n = 4 biological replicates) are given. Differences between WT/EV and RNAi knockdown lines were analyzed by Student's *t*-test (ST) or Mann-Whitney Rank Sum Test (MW).

compound	WT/EV	RNAi	*P*-value	t-value/*T-value*
Nitrogenous compounds				
benzyl cyanide	11.17 ± 1.59	6.67 ± 1.39	0.082	-2.087 (ST)
indole	11.89 ± 2.21	7.76 ± 1.65	0.185	-1.499 (ST)
Green leaf volatiles				
cis-3-hexenyl acetate	19.71 ± 3.72	12.51 ± 1.98	0.139	-1.706 (ST)
Monoterpenoids				
(*E*)-β-ocimene	41.27 ± 5.57	20.01 ± 3.41	0.017	-3.258 (ST)
Sesquiterpenoids				
germacrene D	92.08 ± 8.39	81.56 ± 10.65	0.467	-0.776 (ST)
(*E*)-β-caryophyllene	20.70 ± 2.07	14.45 ± 2.04	0.075	-2.149 (ST)
(*E,E*)-α-farnesene	86.51 ± 8.77	47.20 ± 4.16	0.029	*10.000* (MW)
unidentified sesquiterpene 1	1.74 ± 0.10	1.61 ± 0.26	0.650	-0.477 (ST)
unidentified sesquiterpene 2	1.14 ± 0.04	0.94 ± 0.10	0.103	-1.920 (ST)
unidentified sesquiterpene 3	3.58 ± 0.31	2.62 ± 0.35	0.084	-2.067 (ST)

Supplemental Table S13. Volatiles (ng g^{-1} h^{-1} fresh weight) emitted from *Chrysomela populi*-damaged *Populus* x *canescens* leaves of *cinnamate-CoA ligase (CNL1* and *4)* knockdown (RNAi), empty vector (EV), and wild-type (WT) trees. Compounds were collected for 6 h using a push-pull system and Poropak filter. Eluates were analyzed using GC-MS and quantified by GC-FID. Means and SE (n = 3-6 technical replicates) are given.

**line**	**benzyl cyanide**	**indole**	***cis*-3-hexenyl acetate**	**(*E*)-β-ocimene**	**germacrene D**
**WT**	12.16 ± 4.38	9.00 ± 2.60	26.86 ± 11.72	28.34 ± 6.48	84.82 ± 17.31
**EV-1**	14.43 ± 4.33	17.90 ± 0.54	23.98 ± 11.73	46.28 ± 15.47	105.64 ± 4.38
**EV-2**	11.25 ± 5.00	12.50 ± 4.97	10.03 ± 4.41	36.62 ± 13.55	106.09 ± 21.04
**EV-3**	6.84 ± 1.16	8.17 ± 0.78	17.96 ± 3.95	53.84 ± 12.88	71.76 ± 9.73
**RNAi-1**	10.70 ± 4.18	12.53 ± 4.84	9.26 ± 1.93	16.28 ± 3.32	75.87 ± 5.23
**RNAi-2**	5.54 ± 3.79	5.08 ± 3.21	8.99 ± 3.96	14.87 ± 6.35	69.37 ± 28.52
**RNAi-3**	6.52 ± 2.15	7.26 ± 2.35	16.63 ± 8.04	29.90 ± 10.54	113.08 ± 37.19
**RNAi-4**	4.28 ± 0.84	6.17 ± 0.37	15.18 ± 6.73	18.99 ± 3.50	67.91 ± 12.73

Supplemental Table S13. continued

line	(*E*)-β-caryophyllene	(*E,E*)-α-farnesene	unidentified sesquiterpene 1	unidentified sesquiterpene 2	unidentified sesquiterpene 3
**WT**	16.63 ± 3.37	69.44 ± 17.17	1.69 ± 0.31	1.11 ± 0.19	2.89 ± 0.57
**EV-1**	24.55 ± 5.21	106.21 ± 11.71	1.88 ± 0.15	1.21 ± 0.11	4.33 ± 0.95
**EV-2**	17.66 ± 3.11	74.25 ± 24.73	1.92 ± 0.44	1.20 ± 0.25	3.30 ± 0.56
**EV-3**	23.95 ± 5.35	96.16 ± 9.46	1.48 ± 0.18	1.05 ± 0.13	3.81 ± 0.87
**RNAi-1**	10.46 ± 2.07	37.95 ± 15.12	1.37 ± 0.12	0.77 ± 0.06	1.96 ± 0.37
**RNAi-2**	11.81 ± 4.55	43.68 ± 32.23	1.25 ± 0.47	0.81 ± 0.26	2.19 ± 0.87
**RNAi-3**	19.37 ± 7.36	57.32 ± 20.66	2.38 ± 1.03	1.20 ± 0.36	3.49 ± 1.28
**RNAi-4**	16.18 ± 5.84	49.87 ± 27.86	1.44 ± 0.16	0.97 ± 0.03	2.83 ± 0.98

Supplemental Table S14. Content of non-polar benzenoid compounds (µg/g fresh weight) in *Chrysomela populi*-damaged *Populus* x *canescens* leaves of *cinnamate-CoA ligase (CNL1* and *4)* knockdown (RNAi), empty vector (EV), and wild-type (WT) trees. Compounds were extracted with hexane from fresh plant material and analyzed using GC-MS/GC-FID. NA, not available; nd, not detected. Means and SE (n = 4 biological replicates) are given. Differences between WT/EV and RNAi knockdown lines were analyzed by Student's *t*-test (ST).

compound	WT/EV	RNAi	*P*-value	t-value
**benzaldehyde**	0.87 ± 0.18	0.55 ± 0.08	0.154	-1.633 (ST)
**benzylalcohol**	0.25 ± 0.04	0.17 ± 0.03	0.196	-1.456 (ST)
**salicylaldehyde**	30.15 ± 3.19	24.18 ± 2.63	0.199	-1.444 (ST)
**benzyl benzoate**	0.56 ± 0.06	0.35 ± 0.05	0.037	-2.670 (ST)

Supplemental Table S15. Content of non-polar benzenoid compounds (µg/g fresh weight) in *Chrysomela populi*-damaged *Populus* x *canescens* leaves of *cinnamate-CoA ligase (CNL1* and *4)* knockdown (RNAi), empty vector (EV), and wild-type (WT) trees. Compounds were extracted with hexane from fresh plant material and analyzed using GC-MS/GC-FID. NA, not available; nd, not detected. Means and SE (n = 3-6 technical replicates) are given.

line	benzaldehyde	benzylalcohol	salicylaldehyde	benzyl benzoate
**WT**	0.70 ± 0.16	0.22 ± 0.06	24.84 ± 7.43	0.43 ± 0.08
**EV-1**	0.94 ± 0.09	0.23 ± 0.03	28.77 ± 2.55	0.68 ± 0.15
**EV-2**	0.51 ± 0.08	0.18 ± 0.04	27.59 ± 7.79	0.48 ± 0.12
**EV-3**	1.32 ± 0.33	0.36 ± 0.10	39.41 ± 9.89	0.66 ± 0.09
**RNAi-1**	0.37 ± 0.10	0.11 ± 0.03	19.55 ± 4.37	0.23 ± 0.05
**RNAi-2**	0.51 ± 0.11	0.15 ± 0.02	23.41 ± 3.49	0.36 ± 0.10
**RNAi-3**	0.58 ± 0.13	0.17 ± 0.06	22.07 ± 5.36	0.35 ± 0.11
**RNAi-4**	0.75 ± 0.23	0.27 ± 0.09	31.69 ± 7.88	0.47 ± 0.15

Supplemental Table S16. Benzenoid carboxylic acid content (µg/g dry weight) in *Chrysomela populi*-damaged *Populus* x *canescens* leaves of *cinnamate-CoA ligase (CNL1* and *4)* knockdown (RNAi), empty vector (EV), and wild-type (WT) trees. Compounds were extracted with methanol from freeze-dried plant material and analyzed using LC-MS/MS. BA, benzoic acid; NA, not available; nd, not detected; nq, not quantified due to trace amounts. Means and SE (n = 4 biological replicates) are given. Differences between WT/EV and RNAi knockdown lines were analyzed by Student's *t*-test (ST).

compound	WT/EV	RNAi	*P*-value	t-value
**BA**	nq	nq	NA	NA
**salicylic acid**	nq	nq	NA	NA
**3-hydroxy BA**	nd	nd	NA	NA
**4-hydroxy BA**	2.30 ± 0.31	1.69 ± 0.25	0.175	-1.538 (ST)
**2,3- dihydroxy BA**	1.71 ± 0.28	1.29 ± 0.21	0.28	-1.187 (ST)
**2,5- dihydroxy BA**	nd	nd	NA	NA
**gallic acid**	nd	nd	NA	NA

Supplemental Table S17. Benzenoid carboxylic acid content (µg/g dry weight) in *Chrysomela populi*-damaged *Populus* x *canescens* leaves of *cinnamate-CoA ligase (CNL1* and *4)* knockdown (RNAi), empty vector (EV), and wild-type (WT) trees. Compounds were extracted with methanol from freeze-dried plant material and analyzed using LC-MS/MS. BA, benzoic acid; NA, not available; nd, not detected; nq, not quantified due to trace amounts. Means and SE (n = 3-6 technical replicates) are given.

line	BA	salicylic acid	3-hydroxy BA	4-hydroxy BA	2,3-dihydroxy BA	2,5-dihydroxy BA	gallic acid
**WT**	nq	nq	nd	1.95 ± 0.41	1.31 ± 0.28	nd	nd
**EV-1**	nq	nq	nd	2.66 ± 0.43	2.02 ± 0.36	nd	nd
**EV-2**	nq	nq	nd	1.62 ± 0.56	1.16 ± 0.24	nd	nd
**EV-3**	nq	nq	nd	2.97 ± 0.40	2.35 ± 0.43	nd	nd
**RNAi-1**	nq	nq	nd	1.19 ± 0.28	0.86 ± 0.16	nd	nd
**RNAi-2**	nq	nq	nd	1.55 ± 0.59	1.25 ± 0.43	nd	nd
**RNAi-3**	nq	nq	nd	1.62 ± 0.37	1.16 ± 0.28	nd	nd
**RNAi-4**	nq	nq	nd	2.38 ± 0.80	1.88 ± 0.57	nd	nd

Supplemental Table S18. Phenylpropanoid carboxylic acid content (µg/g dry weight) in *Chrysomela populi*-damaged *Populus* x *canescens* leaves of *cinnamate-CoA ligase (CNL1* and *4)* knockdown (RNAi), empty vector (EV), and wild-type (WT) trees. Compounds were extracted with methanol from freeze-dried plant material and analyzed using LC-MS/MS. NA, not available; nd, not detected. Means and SE (n = 4 biological replicates) are given. Differences between WT/EV and RNAi knockdown lines were analyzed by Student's *t*-test (ST).

compound	WT/EV	RNAi	*P*-value	t-value
**cinnamic acid**	4.56 ± 0.37	5.02 ± 0.40	0.431	0.845 (ST)
**2-hydroxy cinnamic acid**	1.56 ± 0.04	1.39 ± 0.03	0.015	-3.378 (ST)
**4-hydroxy cinnamic acid**	1.35 ± 0.18	1.31 ± 0.17	0.901	-0.130 (ST)
**caffeic acid**	2.39 ± 0.33	1.70 ± 0.28	0.158	-1.613 (ST)
**ferulic acid**	nd	nd	NA	NA

Supplemental Table S19. Phenylpropanoid carboxylic acid content (µg/g dry weight) in *Chrysomela populi*-damaged *Populus* x *canescens* leaves of *cinnamate-CoA ligase (CNL1* and *4)* knockdown (RNAi), empty vector (EV), and wild-type (WT) trees. Compounds were extracted with methanol from freeze-dried plant material and analyzed using LC-MS/MS. NA, not available; nd, not detected. Means and SE (n = 3-6 technical replicates) are given.

line	cinnamic acid	2-hydroxy cinnamic acid	4-hydroxy cinnamic acid	caffeic acid	ferulic acid
**WT**	4.70 ± 0.99	1.60 ± 0.08	0.98 ± 0.13	1.73 ± 0.41	nd
**EV-1**	4.47 ± 0.17	1.63 ± 0.14	1.44 ± 0.19	2.90 ± 0.54	nd
**EV-2**	5.43 ± 1.53	1.45 ± 0.15	1.14 ± 0.27	1.93 ± 0.24	nd
**EV-3**	3.65 ± 0.66	1.56 ± 0.07	1.82 ± 0.24	3.01 ± 0.43	nd
**RNAi-1**	4.43 ± 0.62	1.34 ± 0.16	1.19 ± 0.38	1.08 ± 0.29	nd
**RNAi-2**	4.23 ± 0.40	1.47 ± 0.09	1.07 ± 0.33	1.45 ± 0.53	nd
**RNAi-3**	5.75 ± 0.56	1.34 ± 0.13	1.17 ± 0.28	1.87 ± 0.41	nd
**RNAi-4**	5.68 ± 1.09	1.41 ± 0.19	1.82 ± 0.56	2.38 ± 0.70	nd

Supplemental Table S20. HPLC gradients used for separation and analysis of metabolites.

gradient	time (min)	solvent A (%)	solvent B (%)
gradient A	0.00	95.0	5.0
	0.50	95.0	5.0
	6.00	62.6	37.4
	6.01	20.0	80.0
	7.50	0.0	100.0
	9.50	0.0	100.0
	9.52	95.0	5.0
	12.00	95.0	5.0
gradient B	0.00	90.0	10.0
	0.50	90.0	10.0
	4.00	10.0	90.0
	4.02	0.0	100.0
	4.50	0.0	100.0
	4.51	90.0	10.0
	7.00	90.0	10.0
gradient C	0.00	95.0	5.0
	0.50	95.0	5.0
	9.50	42.0	58.0
	9.52	0.0	100.0
	11.00	0.0	100.0
	11.10	95.0	5.0
	14.00	95.0	5.0
gradient D	0.00	90.0	10.0
	3.00	60.0	40.0
	3.01	0.0	100.0
	4.50	0.0	100.0
	4.51	90.0	10.0
	7.00	90.0	10.0
gradient E	0.00	98.0	2.0
	1.00	98.0	2.0
	4.00	60.0	40.0
	4.01	0.0	100.0
	5.50	0.0	100.0
	5.51	98.0	2.0
	8.00	98.0	2.0

Supplemental Table S21. Parameters used for LC-MS/MS analysis. Details of the HPLC gradients are given in Supplemental Table S20. CE, collision energy; DP, declustering potential; Q1, quadrupole 1; Q3, quadrupole 3.

compound	Q1 (m/z)	Q3 (m/z)	DP (V)	CE (V)	mode	HPLC gradient
phenylalanine	164	147	-30	-18	neg	A
tyrosine	180	163	-30	-18	neg	A
tryptophan	202.9	186	-30	-19	neg	A
trans-cinnamic acid	147	102.8	-30	-16	neg	A/B
2-hydroxycinnamic acid	163	118.9	-30	-20	neg	A/B
4-hydroxycinnamic acid	163	118.9	-30	-20	neg	A/B
caffeic acid	179	134.9	-30	-22	neg	A/B
ferulic acid	193.1	133.9	-30	-22	neg	A/B
gallic acid	169	125	-30	-18	neg	A/B
benzoic acid	121	121	-30	-5	neg	A/B
salicylic acid	137	93	-30	-22	neg	A/B
3-hydroxybenzoic acid	137	93	-30	-16	neg	A/B
4-hydroxybenzoic acid	137	93	-30	-20	neg	A/B
2,3-dihydroxybenzoic acid	153	108/109	-30	-28	neg	A/B
2,5-dihydroxybenzoic acid	153	108	-30	-28	neg	A/B
salicin	331	122.8	-60	-18	neg	A
salicin-7-sulfate	365	97	-60	-25	neg	A
salirepin	301	139	-60	-18	neg	A
salirepin-7-sulfate	381	97	-60	-55	neg	A
salicortin	422.8	123.1	-60	-7,5	neg	A
homaloside D	543	139	-60	-30	neg	A
tremulacin/6-*O*‘-benzoylsalicortin	527.1	123.2	-60	-34	neg	A
D_7-cinnamic acid	154	110	-60	-16	neg	A
D_6-2-hydroxycinnamic acid	169	125	-60	-20	neg	A
D_6-4-hydroxycinnamic acid	169	125	-60	-20	neg	A
D_5-caffeic acid	184	140	-60	-22	neg	A
D_5-ferulic acid	198	139	-60	-22	neg	A
D_5-benzoic acid	126	126	-60	-16	neg	A
D_4-salicylic acid	141	97	-60	-22	neg	A
D_4-3-hydroxybenzoic acid	141	97	-60	-16	neg	A
D_4-4-hydroxybenzoic acid	141	97	-60	-20	neg	A
D_3-2,3-dihydroxybenzoic acid	156	112	-60	-28	neg	A
D_3-2,5-dihydroxybenzoic acid	156	111	-60	-28	neg	A
D_2-gallic acid	171	127	-60	-18	neg	A
D_4-salicin	335	127	-60	-18	neg	A
D_4-salicin-7-sulfate	369	97	-60	-25	neg	A
D_3-salirepin	304	142	-60	-18	neg	A
D_3-salirepin-7-sulfate	384	97	-60	-55	neg	A
D_8-salicortin	431	127	-60	-30	neg	A
D_{12}-homaloside D	555	143	-60	-30	neg	A
D_{13}-tremulacin/6-*O*‘-benzoylsalicortin	540	127	-60	-34	neg	A

Supplemental Table S21. continued

compound	Q1 (m/z)	Q3 (m/z)	DP (V)	CE (V)	mode	HPLC gradient
salicylic acid	136.9	93	-20	-22	neg	C
jasmonic acid	209.1	59	-20	-24	neg	C
abscisic acid	263	153.2	-20	-22	neg	C
(-)-jasmonoyl-L-isoleucine	322.2	130.1	-50	-30	neg	C
(+)-7-*iso*-jasmonoyl-L-isoleucine	322.2	130.1	-50	-30	neg	C
12-oxophytodienoic acid	290.9	165.1	-20	-24	neg	C
12-hydroxyjasmonic acid	225.1	59	-20	-24	neg	C
12-hydroxyjasmonic acid-isoleucine	338.1	130.1	-50	-30	neg	C
12-carboxyjasmonic acid-isoleucine	352.1	130.1	-50	-30	neg	C
D_4-salicylic acid	140.9	97	-20	-22	neg	C
D_6-abscisic acid	269	159.2	-20	-22	neg	C
D_6-jasmonic acid	215	59	-20	-24	neg	C
D_6-jasmonic-acid isoleucine	328.2	130.1	-50	-30	neg	C
cinnamoyl-CoA	896.2	79.0	-30	-110	neg	D
hydroxyl-cinnamoyl-CoA	912.2	79.0	-30	-110	neg	D
caffeoyl-CoA	928.3	79.0	-30	-110	neg	D
feruloyl-CoA	942.2	79.0	-30	-110	neg	D
benzoyl-CoA	870.2	79.0	-30	-110	neg	D
3-oxo-3-phenylpropanoyl-CoA	912.3	79.0	-30	-110	neg	D
3-oxo-3-(2-hydroxy)-phenylpropanoyl-CoA	928.2	79	-30	-110	neg	D
3-oxo-3-(4-hydroxy)-phenylpropanoyl-CoA	928.2	79.0	-30	-110	neg	D
3-oxo-3-(3,4-dihydroxy)-phenylpropanoyl-CoA	944.2	79.0	-30	-110	neg	D
3-oxo-3-(4-hydroxy-3-methoxy)-phenylpropanoyl-CoA	958.2	79.0	-30	-110	neg	D
acetyl-CoA	808.2	79	-30	-110	neg	E

Supplemental Table S22. Oligonucleotides used for the amplification of full-length *Populus trichocarpa* (Pt) *cinnamate-CoA ligases* (*CNL*), *cinnamoyl-CoA hydratases/dehydrogenases* (*CHD*), and *3-ketoacyl-CoA thiolases* (*KAT*). *, forward (fwd) oligonucleotide used for the amplification of *Potri.017G138400* (*PtCNL1*) and *Potri.T116500* (*PtCNL3*), #, reverse (rev) oligonucleotide used for the amplification of *Potri.006G036200* (*PtCNL6*) and *Potri.006G036300* (*PtCNL7*).

oligonucleotides	gene	sequence 5‘ – 3‘	usage
PtCNL1fwd*	*Potri.017G138400*	CACCATGGATCAACTACTAAAATGC	cloning/pET100/D-TOPO®
PtCNL4fwd	*Potri.004G082000*	CACCATGGATCAACTACAAAAATGT	cloning/pET100/D-TOPO®
PtCNL5fwd	*Potri.016G034800*	CACCATGGATCAACTTCCAAAATGT	cloning/pET100/D-TOPO®
PtCNL6fwd	*Potri.006G036200*	CACCATGGATCAGCTTCCAAAGTGT	cloning/pET100/D-TOPO®
PtCNL7fwd	*Potri.006G036300*	CACCATGGATCAGCTTCCAAAATGT	cloning/pET100/D-TOPO®
PtCNL1rev	*Potri.017G138400*	TCAAAGACGAGATAGAGCAAG	cloning/pET100/D-TOPO®
PtCNL3rev	*Potri.T116500*	TCATGCATTGACAACAAAATT	cloning/pET100/D-TOPO®
PtCNL4rev	*Potri.004G082000*	TCAAAGACGAGACATAGCAAG	cloning/pET100/D-TOPO®
PtCNL5rev	*Potri.016G034800*	TCAAAGACGAGAGGAAGGAAC	cloning/pET100/D-TOPO®
PtCNL6rev#	*Potri.006G036200*	TCAAAGACGAGACGAAGCAAC	cloning/pET100/D-TOPO®
PtCHD1fwd	*Potri.018G082900*	CACCATGGCTAAGCCTCACGTCACC	cloning/pET100/D-TOPO®
PtCHD2fwd	*Potri.010G011900*	CACCATGGAAGGAAGCAGGACTAAG	cloning/pET100/D-TOPO®
PtCHD3fwd	*Potri.008G220100*	CACCATGAAAGGAAGCAGGACCAAG	cloning/pET100/D-TOPO®
PtCHD1rev	*Potri.018G082900*	CTACATGCGTGACCTTGACCC	cloning/pET100/D-TOPO®
PtCHD2rev	*Potri.010G011900*	TTACAGCCGAGACTTTGCTTGG	cloning/pET100/D-TOPO®
PtCHD3rev	*Potri.008G220100*	TTACAGCCGAGACTTCGCTTGC	cloning/pET100/D-TOPO®
PtKAT1fwd	*Potri.002G216400*	CACCATGGAGAAGGCATTTAACAGA	cloning/pET100/D-TOPO®
PtKAT2fwd	*Potri.001G051900*	CACCATGGAGAAAGCAATCAACAGG	cloning/pET100/D-TOPO®
PtKAT3fwd	*Potri.001G051800*	CACCATGGAGAAAGCAAGCAACAGG	cloning/pET100/D-TOPO®
PtKAT1rev	*Potri.002G216400*	CTAACGAGCATCTTTTGACAG	cloning/pET100/D-TOPO®
PtKAT2rev	*Potri.001G051900*	CTATCGTGAATCCCTGGATAA	cloning/pET100/D-TOPO®
PtKAT3rev	*Potri.001G051800*	CTATCTTGAATCCTTGGATAA	cloning/pET100/D-TOPO®

Supplemental Table S23. Oligonucleotides used for gene expression analysis of *Populus trichocarpa* (Pt) *cinnamate-CoA ligases* (*CNL*), *cinnamoyl-CoA hydratases/dehydrogenases* (*CHD*), and *3-ketoacyl-CoA thiolases* (*KAT*) by RT-qPCR.

oligonucleotides	gene	sequence 5‘ – 3‘	usage
PtCNL1fwd	*Potri.017G138400*	CTGATATTATCGCTTACTGC	RT-qPCR
PtCNL3fwd	*Potri.T116500*	CGAAATCACTTCCCCGGTTG	RT-qPCR
PtCNL4fwd	*Potri.004G082000*	CCACCAGCCTCATTGCTTGA	RT-qPCR
PtCNL5fwd	*Potri.016G034800*	TTGCTCAGCACAAGGTGACT	RT-qPCR
PtCNL6fwd	*Potri.006G036200*	CAAACCAGAGGAGCGTAGGG	RT-qPCR
PtCNL7fwd	*Potri.006G036300*	GGAAAAAGCTTCCCCGTTATATGG	RT-qPCR
PtCNL1rev	*Potri.017G138400*	TTGCTTGGCAATATCTCATTG	RT-qPCR
PtCNL3rev	*Potri.T116500*	TCCTGCTGTGGAAGCTTGTT	RT-qPCR
PtCNL4rev	*Potri.004G082000*	ACTGATCCTGCTGTGGAAGC	RT-qPCR
PtCNL5rev	*Potri.016G034800*	GGTGCACCACCGACAAGTAT	RT-qPCR
PtCNL6rev	*Potri.006G036200*	AACCTCCGTGAGGCCATAAG	RT-qPCR
PtCNL7rev	*Potri.006G036300*	CTTGCATTTCTGTATTGGCATTTAC	RT-qPCR
PtCHD1fwd	*Potri.018G082900*	ATCTCAGAGAGGCGCAAACC	RT-qPCR
PtCHD2fwd	*Potri.010G011900*	CGAGCCCCCAATCTAACACA	RT-qPCR
PtCHD3fwd	*Potri.008G220100*	CAAGTTTGCTAGAGCACAAGC	RT-qPCR
PtCHD1rev	*Potri.018G082900*	CTTGATGCTGCGGCACATTT	RT-qPCR
PtCHD2rev	*Potri.010G011900*	AGGTGTCAGAACGCACAAGT	RT-qPCR
PtCHD3rev	*Potri.008G220100*	GTGGACCAAGCTCTTGCTGA	RT-qPCR
PtKAT1fwd	*Potri.002G216400*	AGTGCATGACAGTGAACGGC	RT-qPCR
PtKAT2fwd	*Potri.001G051900*	CAAGAAGTTGGAGCTTGATCCA	RT-qPCR
PtKAT3fwd	*Potri.001G051800*	GGATTCGCCCAAACACATCGT	RT-qPCR
PtKAT1rev	*Potri.002G216400*	CGCCATAACGCTGAGCAACA	RT-qPCR
PtKAT2rev	*Potri.001G051900*	ACATTGACACCACCCCGAAG	RT-qPCR
PtKAT3rev	*Potri.001G051800*	GATGGGTAACCCTTTGCGCAT	RT-qPCR

Supplemental Table S24. Expression levels of potential housekeeping genes in undamaged (control) and *Chrysomela populi-damaged* (herbivory) *Populus trichocarpa* leaves. The ΔCq means (n = 4) and the standard deviations (STDEV) are shown. *UBQ, ubiquitin; TUB, tubulin; HIS, histone superfamily protein H3; EF1α, elongation factor 1 alpha*; and *actin* (Ramírez-Carvajal et al., 2008; Xu et al., 2011; Wang et al., 2014).

**treatment**	***UBQ***	***TUB***	***HIS***	***EF1-α***	***Actin***
control	19.50	23.65	20.92	22.17	22.54
herbivory	19.55	23.69	20.99	22.29	22.43
STDEV	0.04	0.03	0.04	0.08	0.07
